# Associations of Military-Related Traumatic Brain Injury With New-Onset Mental Health Conditions and Suicide Risk

**DOI:** 10.1001/jamanetworkopen.2023.26296

**Published:** 2023-07-31

**Authors:** Lisa A. Brenner, Jeri E. Forster, Jaimie L. Gradus, Trisha A. Hostetter, Claire A. Hoffmire, Colin G. Walsh, Mary Jo Larson, Kelly A. Stearns-Yoder, Rachel Sayko Adams

**Affiliations:** 1 VHA Rocky Mountain Mental Illness Research Education and Clinical Center, Aurora, Colorado; 2 University of Colorado, Anschutz Medical Campus, Aurora; 3 Department of Epidemiology, Boston University School of Public Health, Boston, Massachusetts; 4 Departments of Biomedical Informatics, Medicine, and Psychiatry, Vanderbilt University Medical Center, Durham, North Carolina; 5 Institute for Behavioral Health, The Heller School for Social Policy and Management, Brandeis University, Waltham, Massachusetts; 6 Department of Health Law, Policy and Management, Boston University School of Public Health, Boston, Massachusetts

## Abstract

**Importance:**

Research to identify the direct and indirect associations of military-related traumatic brain injury (TBI) with suicide has been complicated by a range of data-related challenges.

**Objective:**

To identify differences in rates of new-onset mental health conditions (ie, anxiety, mood, posttraumatic stress, adjustment, alcohol use, and substance use disorders) among soldiers with and without a history of military-related TBI and to explore the direct and indirect (through new-onset mental health disorders) associations of TBI with suicide.

**Design, Setting, and Participants:**

This retrospective cohort study used data from the Substance Use and Psychological Injury Combat Study (SUPIC) database. Demographic, military, and health data from the Department of Defense within SUPIC were compiled and linked with National Death Index records to identify deaths by suicide. Participants included US Army soldiers who returned from an Afghanistan or Iraq deployment. Data were analyzed from September to December 2022.

**Exposures:**

Military-related TBI.

**Main Outcomes and Measures:**

The outcome of interest was suicide. Secondary outcomes were incidence of new-onset mental health conditions. Mediation analyses consisted of accelerated failure time (AFT) models in conjunction with the product of coefficients method. The 6 new-onset mental health diagnosis categories and the 2 or more categories variable were each considered separately as potential mediators; therefore, a total of 14 models plus the overall AFT model estimating the total effect associated with TBI in suicide risk were fit.

**Results:**

The study included 860 892 soldiers (320 539 soldiers [37.2%] aged 18-24 at end of index deployment; 766 454 [89.0%] male), with 108 785 soldiers (12.6%) with at least 1 documented TBI on their military health record. Larger increases in mental health diagnoses were observed for all conditions from before to after documented TBI, compared with the matched dates for those without a history of TBI, with increases observed for mood (67.7% vs 37.5%) and substance use (100% vs 14.5%). Time-to-suicide direct effect estimates for soldiers with a history of TBI were similar across mediators. For example, considering new-onset adjustment disorders, time-to-suicide was 16.7% faster (deceleration factor, 0.833; 95% CI, 0.756-0.912) than for soldiers without a history of TBI. Indirect effect estimates of associations with TBI were substantial and varied across mediators. The largest indirect effect estimate was observed through the association with new-onset substance use disorder, with a time to suicide 63.8% faster (deceleration factor, 0.372; 95% CI, 0.322-0.433) for soldiers with a history of TBI.

**Conclusions and Relevance:**

In this longitudinal cohort study of soldiers, rates of new-onset mental health conditions were higher among individuals with a history of TBI compared with those without. Moreover, risk for suicide was both directly and indirectly associated with history of TBI. These findings suggest that increased efforts are needed to conceptualize the accumulation of risk associated with multiple military-related exposures and identify evidence-based interventions that address mechanisms associated with frequently co-occurring conditions.

## Key Points

**Question:**

Is military-related traumatic brain injury (TBI) associated with increased incidence of new-onset mental health conditions and suicide risk?

**Findings:**

In this cohort study including 860 892 soldiers, individuals with a history of military-identified TBI had significantly higher rates of new-onset mental health conditions than those without TBI. Increased risk for suicide was associated indirectly (through new-onset mental health diagnoses) and directly with history of TBI.

**Meaning:**

These findings suggest that conceptualizing exposures (physical, psychological) as events that accumulate over an individual’s lifetime and increase risk for negative outcomes (eg, suicide) may assist in identifying mechanisms underlying frequently co-occurring conditions, as well as evidence-based interventions.

## Teaser

This cohort study examines rates of new-onset mental health conditions among soldiers with and without a history of military-related traumatic brain injury and explores direct and indirect associations of traumatic brain injury with suicide risk.

## Introduction

Whereas traumatic brain injury (TBI) and mental health disorders, including posttraumatic stress disorder (PTSD), are frequently discussed as conditions related to military service in the wars in Afghanistan and Iraq, research to date has primarily focused on the parsing of associations of conditions, individually or in combination, with outcomes of interest (eg, suicide^1^). For example, a study by Fisher et al^2^ used group comparisons to identify prevalence of lifetime suicidal ideation, current suicidal ideation, and lifetime suicidal behavior among healthy military and civilian individuals and individuals with mild TBI (mTBI), PTSD, or both conditions and found the highest frequencies of these outcomes among those with both mTBI and PTSD. Moreover, Skopp and colleagues^1^ conducted a case-control study of active members of the US Armed Forces (2001-2009) and found that increased odds of death by suicide were associated with mood disorders, partner relationship problems, and family circumstance problems, but not mTBI, alcohol dependence, or PTSD. Interestingly, these parsing efforts persisted despite research efforts conducted early in the conflicts that suggested that military personnel with a history of TBI had increased risk for developing mental health conditions (eg, PTSD^3^).

Although researchers, health care practitioners, and those with a history of these frequently co-occurring conditions have long been aware of the complicated relationships among TBI, mental health conditions, and negative outcomes, clinical and data-related challenges have created significant research roadblocks, which in turn have impeded advancement of evidenced-based care. For example, overlapping symptoms among individuals with TBI and mental health conditions^4^ have contributed to challenges with differential diagnosis and subsequent documentation. In addition, data-related challenges have been noted in terms of identifying suicide-related outcomes using electronic medical record data,^5^ as well as TBI case ascertainment using *International Classification of Diseases, Ninth Revision *(*ICD-9*) and *International Statistical Classification of Diseases and Related Health Problems, Tenth Revision *(*ICD-10*) codes.^6^

Furthermore, research in this area has often been conducted with no or limited attention to the timing of TBI and mental health diagnoses. This methodological omission is crucial, as mental health diagnoses may be confounders, mediators, and/or modifiers of the association between TBI and suicide. Investigators’ assumptions about the form of these associations should dictate both the timeframe of variable measurement (eg, confounders occur before exposure, mediators occur between exposure and outcome) and the analytic approach (eg, statistical control vs mediation analyses). Accordingly, it has been documented, using PTSD, depression, and suicide as examples, that inappropriate adjustment for important comorbid mental health diagnoses can create bias rather than control it.^7,8^

As such, analyses aimed at identifying associations among TBI, mental health conditions, and suicide would ideally be conducted with large longitudinal data sets that allow for identification of preexisting mental health conditions, an index TBI event, post-TBI new-onset mental health conditions, and death by suicide using the criterion standard National Death Index (NDI) data.^9^ Toward this end, we have evaluated rates of new-onset psychiatric conditions among soldiers with and without a history of TBI during military service, whether history of TBI was associated with increased risk for suicide, and whether new-onset psychiatric conditions while in military service mediate the association between TBI and suicide.

## Methods

This cohort study was approved by all necessary institutional review boards, and as the study was retrospective in nature, a waiver of consent was granted. This study is reported following the Strengthening the Reporting of Observational Studies in Epidemiology (STROBE) reporting guideline for cohort studies.

### Data Sources

Brandeis researchers developed Substance Use and Psychological Injury Combat Study (SUPIC), a longitudinal database that includes soldiers, including US Army Active Duty, Army National Guard, and Army Reserve members returning from an Operation Enduring Freedom, Operation Iraqi Freedom, or Operation New Dawn deployment between fiscal years (FY) 2008 and 2014.^10^ Soldiers were longitudinally followed-up from the end of their first deployment during the study window (ie, index deployment). Given that the study window begins in FY 2008, some soldiers may have deployed prior to the index deployment, and some may have deployed after this index deployment. Deployment data were obtained from the Contingency Tracking System. Demographic characteristics were drawn from the Department of Defense’s (DOD) Defense Enrollment Eligibility Records System. *ICD-9* and *ICD-10* codes from encounter data were obtained from the DOD’s Military Health System (MHS) and are inclusive of outpatient and inpatient direct and purchased care.

Suicide and all cause death data were drawn from the NDI,^9^ the criterion standard for capturing cause and date of death. Data were obtained from the Department of Veterans Affairs DOD Mortality Data Repository^11^ through December 31, 2018.

The analytic cohort was constructed from the original SUPIC cohort of 865 640 soldiers.^12^ All original records, excluding 141 without a usable Social Security Number, were searched in the NDI data and then merged with the SUPIC data. Any record in which death occurred prior to the end of the index deployment was removed (1123 records). Other inclusion criteria included presence of data for military component at the end of index deployment, index deployment length 30 days up to 5 years, and for records with a match in the Veterans Health Administration medical record data, we required Social Security Number, date of birth, and sex assigned in the medical record consistency. These final 3 criteria resulted in the removal of 3446 records for a base SUPIC suicide risk cohort of 860 930 records (99.5% of original SUPIC file; for more information regarding the SUPIC suicide risk cohort, see Adams et al^12^). For this analysis, the exposed group consisted of 108 785 persons with a history of military-related TBI. Soldiers without a history of MHS-documented TBI were matched to an individual with a history of TBI using categories of FY of return and years of MHS data available prior to the index deployment. The match date was used solely to determine timing of mental health diagnoses in the group without an MHS-documented history of TBI. Thirty-eight soldiers without a history of TBI were removed because they could not be matched using this process, resulting in a final analytic cohort of 860 892 individuals. Additional details regarding the matching process are provided in the eMethods in Supplement 1.

### Measures

#### Demographics

SUPIC data files captured age at the end of the index deployment, sex assigned in the medical record, race and ethnicity, military-related information (eg, rank), and FY of return from index deployment. Race and ethnicity are captured in the Military Heath System Data Repository based on self-report by military members and were collapsed by the DOD to create the following racial and ethnic groups: American Indian or Alaska Native; Asian or Pacific Islander; Black, non-Hispanic; White, non-Hispanic; Hispanic; other (ie, military member-reported other for race and other or unknown for ethnicity); or unknown (ie, missing data for both race and ethnicity). Race and ethnicity were included to increase understanding regarding the study population.

#### History of TBI Noted in the MHS

History of TBI (both deployment- and nondeployment-related) noted in the MHS was determined based on criteria outlined in Adams et al^6^ (ie, the Rocky Mountain Mental Illness, Research, Education and Clinical Center TBI code set plus precise DOD-unique codes). Qualifying *ICD-9* and *ICD-10* codes are provided in eTable 1 in Supplement 1. The first documented qualifying TBI diagnosis within all available MHS encounter data, including data available prior to the end of the index deployment, was taken as the index TBI, and the associated date was used in all further calculations. In-theater data (during deployment) were not available; however, we captured all diagnoses that were recorded in the available MHS data and all soldiers in the cohort received care in the MHS following return from the index deployment.

#### Mental Health Diagnoses

Mental health diagnoses were grouped into 6 categories: anxiety, mood, adjustment, alcohol use, substance use (excluding alcohol and tobacco), and posttraumatic stress disorders. Qualifying *ICD-9* and *ICD-10* codes by classification are presented in eTable 2 in Supplement 1. Any qualifying mental health diagnosis that was documented on the day of or before the qualifying TBI date (or match date for those without a history of TBI) was considered a pre-TBI or pre–match date mental health diagnosis. Any qualifying mental health diagnosis that was documented after the qualifying TBI date (or match date) was considered a post-TBI or post–match date diagnosis. To qualify as a new-onset mental health diagnosis, we required a post-TBI or post–match date mental health diagnosis and no documented diagnosis within the same mental health category before the TBI or match date. Lastly, a variable for 2 or more new-onset mental health categories was created.

#### Death by Suicide

Death by suicide was determined by identifying Mortality Data Repository NDI records. Suicide deaths included *ICD-10* codes X60-X84 and Y87.0 as the underlying cause of death.

### Statistical Analysis

The number and percentage of soldiers with pre-, post-, and new-onset post-TBI or match date mental health diagnoses were calculated. Mediation analyses consisted of accelerated failure time (AFT) models in conjunction with the product of coefficients method.^13^ Observations were censored at date of death due to causes other than suicide or at the end of the study timeframe, December 31, 2018. AFT model distributions for survival time (Weibull, exponential, lognormal, logistic, log-logistic, and Gaussian) were compared using Akaike Information Criteria within the model that included history of TBI (yes or no), age category (18-24, 25-29, 30-34, 35-39, and ≥40 years), sex assigned in the medical record, race and ethnicity (American Indian or Alaska Native, Asian or Pacific Islander; Black, non-Hispanic; White, non-Hispanic; Hispanic; and other or unknown) and FY of return from index deployment (2008-2009, 2010-2011, or 2012-2014). The log-logistic distribution had the lowest Akaike Information Criteria and was used for all further AFT models.

The need to control for mental health diagnoses that occurred prior to TBI was then examined. The base log-logistic AFT model was run with the addition of each mental health diagnosis category, for a total of 6 test models. The percentage change in the estimated ratio of the expected survival times (TBI vs no TBI) between the base model and each model that included the diagnosis categories was calculated to assess for potential confounding. All models resulted in a less than 10% change (range, 2.2%-9.1%) and as such, preexisting mental health conditions were not considered confounders in the models that followed.^14^

The product of coefficients (*a* × *b*) method requires 2 models be fit, 1 that estimates the association of TBI with new-onset mental health diagnosis (coefficient *a*), including age, sex assigned in the medical record, race and ethnicity, and FY of return from index deployment as covariates, and 1 that estimates the association of TBI with suicide while controlling for the mediator (direct effect) and the association of the mediator with suicide (coefficient *b*) with inclusion of the mediator and the same covariates. Given that the prevalence of new-onset mental health diagnoses is not rare (>10%), Poisson regression with robust error variance^15^ was used to estimate coefficient *a*. The base log-logistic AFT model was fit with the addition of the mediator of interest to estimate coefficient *b* and the direct association of TBI with suicide. Additional details regarding the models used for mediation are provided in the eMethods in Supplement 1. The 6 new-onset mental health diagnosis categories and the 2 or more categories variable, were each considered separately as potential mediators; therefore, a total of 14 models plus the overall AFT model estimating the total effect associated with TBI in suicide risk were fit. The point estimates reported are from models fit to the original analytic data set, including the estimate of the indirect effect associated with TBI mediated through the new-onset mental health category, defined as *a* × *b*. The 95% CIs for the total and direct effect estimates associated with TBI, the TBI relative risk for new-onset mental health conditions, and the associations of the mediator with suicide were similarly estimated from these models. The 95% CIs for the indirect effect estimates were calculated using 1000 bootstrap data sets for each new-onset mental health category (*N* equal to the original data set and observations sampled from the original data set with replacement). For each data set, an AFT model with the mediator and covariates was fit and a Poisson regression was fit including covariates. The product of coefficients was calculated for each set of models, resulting in 1000 indirect effect estimates, from which the 2.5% and 97.5% estimates were taken as the lower and upper bounds of the 95% CIs for the indirect effect estimates. All estimates were exponentiated to obtain interpretable results. For all models, we present estimates with associated 95% CIs in accordance with guidance by Perneger^16^ and Rothman,^17^ to allow readers to judge clinical and statistical significance. All analyses were run in either SAS version 9.4 (SAS Institute) or R version 4.1.1 (R Project for Statistical Computing). Data were analyzed from September to December 2022.

## Results

Among the study sample of 860 892 soldiers (320 539 soldiers [37.2%] aged 18-24 years at end of index deployment; 766 454 [89.0%] male), 108 785 soldiers (12.6%) had a history of TBI. Demographic, military, and TBI-related characteristics are provided in Table 1. The cohort included 7916 American Indian or Alaska Native soldiers (0.9%), 68 698 Asian or Pacific Islander soldiers (8.0%), 143 344 Black non-Hispanic soldiers (16.7%), 91 360 Hispanic soldiers (10.6%), and 539 411 White non-Hispanic soldiers (62.7%). Among soldiers with a history of TBI, 458 (0.4%) died by suicide, compared with 2237 soldiers (0.3%) without a history of TBI.

**Table 1.** Demographic and Military Characteristics

Characteristic	Individuals, No. (%)		
	Overall (N = 860 892)	History of TBI (n = 108 785)	No history of TBI (n = 752 107)
Age category at end of index deployment, y			
18-24	320 539 (37.2)	40 932 (37.6)	279 607 (37.2)
25-29	217 269 (25.2)	28 342 (26.1)	188 927 (25.1)
30-34	117 581 (13.7)	16 295 (15.0)	101 286 (13.5)
35-39	91 999 (10.7)	12 197 (11.2)	79 802 (10.6)
≥40	113 504 (13.2)	11 019 (10.1)	102 485 (13.6)
Sex assigned in the medical record			
Male	766 454 (89.0)	100 766 (92.6)	665 688 (88.5)
Female	94 438 (11.0)	8019 (7.4)	86 419 (11.5)
Race and ethnicity			
American Indian or Alaskan Native	7916 (0.9)	1195 (1.1)	6721 (0.9)
Asian or Pacific Islander	68 698 (8.0)	10 768 (9.9)	57 930 (7.7)
Black non-Hispanic	143 344 (16.7)	15 847 (14.6)	127 497 (17.0)
Hispanic	91 360 (10.6)	12 804 (11.8)	78 556 (10.4)
White non-Hispanic	539 411 (62.7)	66 787 (61.4)	472 624 (62.8)
Other^a^	7838 (0.9)	1159 (1.1)	6679 (0.9)
Unknown or missing^b^	2325 (0.3)	225 (0.2)	2100 (0.3)
Fiscal year of return from index deployment			
2008-2009	316 420 (36.8)	47 383 (43.6)	269 037 (35.8)
2010-2011	326 101 (37.9)	41 579 (38.2)	284 522 (37.8)
2012-2014	218 371 (25.4)	19 823 (18.2)	198 548 (26.4)
Rank group			
Junior enlisted (E1-E4)	413 451 (48.0)	51 260 (47.1)	362 191 (48.2)
Senior enlisted (E5-E9) or warrant officer	339 195 (39.4)	48 861 (44.9)	290 334 (38.6)
Officer	108 241 (12.6)	8663 (8.0)	99 578 (13.2)
Missing	5 (<0.1)	1 (<0.1)	4 (<0.1)
Index deployment group			
First deployers	598 307 (69.5)	65 780 (60.5)	532 527 (70.8)
≥2 Deployers	262 585 (30.5)	43 005 (39.5)	219 580 (29.2)

Abbreviation: TBI, traumatic brain injury.

^a^ Includes individuals reported other for race and other or unknown for ethnicity.

^b^ Includes individuals who selected unknown in race and ethnicity and individuals without race and ethnicity data.

### Mental Health Diagnoses Relative to TBI

While mental health diagnoses before TBI or match dates were not found to confound the association between TBI and suicide, they were higher for soldiers with a history of TBI, compared with those without a history of TBI (Table 2). Additionally, higher increases in rates of mental health diagnoses from before to after TBI or match dates were observed for soldiers with TBI. When examining mood disorder diagnoses, soldiers with TBI had an increase from 24 460 soldiers (22.5%) before the TBI to 40 997 soldiers (37.7%) following the TBI (a 67.7% increase) compared with an increase from 62 363 soldiers (8.3%) to 85 731 soldiers (11.4%) among those without TBI (a 37.5% increase). Soldiers with a history of TBI had a 31.9% increase in alcohol use disorders, while soldiers without a history of TBI had only a 10.3% increase (Table 2). The largest disparity was observed for substance use disorders, in which soldiers with a history of TBI had a 100% increase compared with a 14.5% increase among soldiers without a history of TBI (Table 2). These differences were primarily due to markedly higher rates of new-onset mental health diagnoses among those with TBI. For example, new-onset anxiety occurred for 27 882 soldiers (25.6%) with TBI, compared with only 73 786 soldiers (9.8%) without TBI.

**Table 2.** Mental Health Diagnosis Category by TBI Status

Diagnosis category	History of TBI (n = 108 785)				No history of TBI (n = 752 107)			
	No. (%)		Before vs after change, %	New-onset after TBI, No. (%)	No. (%)		Before vs after change, %	New onset after match date, No. (%)
	Before TBI	After TBI			Before match date	After match date		
Anxiety	25 775 (23.7)	45 046 (41.4)	74.8	27 882 (25.6)	55 613 (7.4)	90 231 (12.0)	62.4	73 786 (9.8)
Mood	24 460 (22.5)	40 997 (37.7)	67.7	24 326 (22.4)	62 363 (8.3)	85 731 (11.4)	37.5	66 631 (8.9)
PTSD	22 592 (20.8)	44 204 (40.6)	95.6	26 044 (23.9)	30 320 (4.0)	57 723 (7.7)	90.3	48 347 (6.4)
Adjustment	33 144 (30.5)	45 526 (41.9)	37.3	25 960 (23.9)	85 757 (11.4)	106 275 (14.1)	23.9	83 128 (11.1)
Alcohol use	14 035 (12.9)	18 518 (17.0)	31.9	11 402 (10.5)	37 884 (5.0)	41 808 (5.6)	10.3	34 279 (4.6)
Substance use	5295 (4.9)	10 616 (9.8)	100	8392 (7.7)	17 567 (2.3)	20 131 (2.7)	14.5	17 847 (2.4)

Abbreviations: PTSD, posttraumatic stress disorder; TBI, traumatic brain injury.

### Mental Health Mediation

For the total association of TBI with suicide, the time to suicide for those with a history of TBI was 21.3% faster (deceleration factor, 0.787; 95% CI, 0.715-0.866) than for those without a history of TBI, after accounting for age, sex assigned in the medical record, race and ethnicity, and FY of return from index deployment (Table 3). The direct effect estimate of TBI on suicide ranged from a time to suicide for soldiers with TBI 8.5% faster (deceleration factor, 0.915; 95% CI, 0.829-1.010) than those without a TBI for the 2 or more mental health diagnoses category model, to a time to suicide for soldiers with TBI 16.7% faster (deceleration factor, 0.833; 95% CI, 0.756-0.918) than those without a TBI for the adjustment disorder model. The largest indirect effect estimate of TBI on suicide was observed for the substance use model, such that for soldiers with TBI, the time to suicide was 62.8% faster (deceleration factor, 0.372; 95% CI, 0.322-0.433) through the occurrence of a new-onset substance use disorder, compared with soldiers without TBI. Indirect effect estimates were of similar magnitude for alcohol use disorders, PTSD, mood disorders, and 2 or more mental health condition categories, while there was a smaller indirect effect estimate for anxiety and adjustment disorders (Table 3).

**Table 3.** Mediation Model Results for the Association of TBI With Suicide

New onset mental health category (mediator)	Estimate (95% CI)			
	Direct effect deceleration factor^a^	TBI relative risk for mental health category^b^	Mediator deceleration factor^a^	Indirect effect deceleration factor^a^
Anxiety	0.834 (0.756-0.920)	2.61 (2.58-2.64)	0.725 (0.656-0.802)	0.735 (0.670-0.814)
Mood	0.874 (0.792-0.964)	2.52 (2.49-2.58)	0.540 (0.490-0.596)	0.566 (0.518-0.622)
PTSD	0.863 (0.781-0.953)	3.63 (3.58-3.68)	0.641 (0.574-0.716)	0.563 (0.485-0.653)
Adjustment	0.833 (0.756-0.918)	2.14 (2.11-2.17)	0.686 (0.623-0.755)	0.750 (0.700-0.810)
Alcohol	0.852 (0.773-0.938)	2.19 (2.15-2.24)	0.418 (0.374-0.467)	0.504 (0.460-0.551)
Substance	0.848 (0.769-0.935)	3.10 (3.02-3.18)	0.417 (0.364-0.478)	0.372 (0.322-0.433)
≥2 Categories	0.915 (0.829-1.01)	2.69 (2.66-2.72)	0.538 (0.492-0.588)	0.541 (0.495-0.591)

Abbreviations: PTSD, posttraumatic stress disorder; TBI, traumatic brain injury.

^a^ Point estimates were taken from the accelerated failure time models including TBI and the mediator of interest, and controlling for age category (18-24, 25-29, 30-34, 35-39, and ≥40 years), race and ethnicity (American Indian or Alaskan Native, Asian American or Pacific Islander, Black non-Hispanic, White non-Hispanic, Hispanic, and other or unknown), sex assigned in the medical record, and fiscal year of return from index deployment (2008-2009, 2010-2011, 2012-2014).

^b^ Estimated from Poisson models, including TBI and controlling for the same covariates as the accelerated failure time models.

## Discussion

Results from this cohort study support the assertion that among military personnel, death by suicide is both directly and indirectly (through new-onset mental health conditions) associated with a history of TBI. Most previous work, including by members of this team,^18^ has primarily focused on highlighting the unique association between TBI and suicide, without consideration of whether mental health conditions were confounders, mediators, or both. However, the finding that all models evaluating direct (TBI to suicide) and indirect (TBI through new-onset mental health diagnoses to suicide) associations were significant for single-category new-onset diagnoses, highlights the importance of rethinking methodological strategies being used to increase understanding regarding complex associations of exposures with proximal and distal outcomes of interest. For example, in a 2022 article, Miller and colleagues^19^ presented findings from a matched case-control design and found that psychiatric disorders mediated less than 30% of the association between TBI and suicidal ideation or attempt.

Our findings when considering 2 or more mental health categories were not statistically significant, and compared with single-category mental health models, the decrease in magnitude of the direct effect estimate was slight and the indirect effect estimate was similar. These findings are consistent with previous work in which associations between TBI and specific mental health diagnoses vary.^20,21,22^ Moreover, to date, limited work has been conducted among individuals with a history of TBI regarding specific mental health diagnoses and risk for death by suicide. Increased understanding regarding these associations may provide additional insights regarding mechanisms (eg, inflammation) underlying this increased risk and lead to strategies for intervention or prevention.^23^

Also important are our findings regarding the differential rate of new-onset mental health conditions identified among soldiers with a documented history of TBI. Across diagnoses, the frequencies of new-onset mental health diagnoses were more than double among the group with TBI compared with soldiers without this documented history of injury. These findings are consistent with literature among military members,^3^ veterans, and civilians.^24^

Regarding substance use disorders, we found that the largest indirect effect of TBI on suicide was through new-onset substance use disorders, followed by alcohol use disorders, compared with soldiers without TBI. These findings are consistent with studies with military, veteran, and civilian populations that have found that individuals with TBI are at increased risk for substance use disorders and negative outcomes.^25^ In particular, we highlight timely work regarding the opioid epidemic, disproportionate receipt of prescription opioids among individuals with TBI, and associated adverse consequences.^26,27^

Although research has been conducted regarding the enduring health outcomes associated with TBIs sustained during the recent conflicts in Iraq and Afghanistan^28^ (eg, persistent postconcussive symptoms, even after adjusting for mental health conditions), less work to date has been focused on how new-onset conditions after TBI are associated with more distal physical and mental health outcomes. In 2009, Brenner et al^29,30^ theorized that outcomes associated with multiple exposures (eg, history of TBI, PTSD) should be conceptualized cumulatively (ie, burden of adversity hypothesis). Findings from this study support adopting a life-course approach when exploring the association of even mild TBI with long-term health outcomes.^29^ Further support for this hypothesis is provided by Brenner and colleagues^31^ in semistructured interviews among Operation Enduring Freedom and Operation Iraqi Freedom Army personnel to elicit information about soldiers’ experiences regarding exposure to physical trauma and emotionally distressing events. Findings from these interviews suggested that “boundaries between events that resulted in (physical/emotional) injury and subsequent symptoms were often fluid, with symptoms more traditionally associated with mTBI or PTSD being attributed to either or both conditions.”^31^ Brenner et al^31^ also noted that soldiers consistently highlighted the compounding effects of experiences and symptoms over 1 or more deployments.

### Limitations

This study has some limitations. First, although access to pre-TBI and post-TBI data within the MHS allowed for exploration of direct and indirect associations, these data, as well as history of all TBIs, are certainly incomplete. Differential diagnosis in terms of health conditions sustained by soldiers who served in combat has been challenging, in part secondary to overlapping symptoms. Additionally, analyses were conducted using diagnoses from electronic medical records and thus only included diagnosed conditions, which are likely to be underestimates of true prevalence. In addition, the likelihood of individuals having 1 or more diagnoses (TBI and/or mental health conditions) may have been impacted by the amount of care received. Moreover, while our method for assessing potential confounding due to pre-TBI mental health diagnoses did not indicate that these variables were confounders in our data, it is possible that these variables will act as confounders of the TBI and suicide association in other samples and data sources. Furthermore, although beyond the scope of the current effort, increased understanding regarding the associations between TBI and mental health history could be obtained by studying alternate cohorts of interest (eg, individuals with a history of physical trauma). That is, it will be important to replicate our results in other samples to further examine the varying potential complex associations among these variables.

## Conclusions

This cohort study found high rates of new-onset mental health diagnoses among soldiers with a history of military-related TBI, as well as such injuries being directly and indirectly associated with suicide risk. These findings support adopting methodological strategies aimed at evaluating risk over an individual’s lifetime, with a focus on how events and conditions accumulate both proximally and distally. In addition, efforts to identify evidence-based interventions that address mechanisms associated with frequently co-occurring conditions (ie, TBI and mental health disorders) are needed.

## Notes

Supplement 1.
eMethods.
**eTable 1.** TBI *ICD-9* and *ICD-10* Codes **eTable 2.** Mental Health *ICD-9* and *ICD-10* Codes and Classifications

Supplement 2.
Data Sharing Statement
