# Supplementary material for: Associations of Military-Related Traumatic Brain Injury With New-Onset Mental Health Conditions and Suicide Risk
Source: JAMA Netw Open. 2023 Jul 31;6(7):e2326296. doi: 10.1001/jamanetworkopen.2023.26296 (PMC10391302; doi:10.1001/jamanetworkopen.2023.26296)
Supplement: Supplement 1. — eMethods. eTable 1. TBI ICD-9 and ICD-10 Codes eTable 2. Mental Health ICD-9 and ICD-10 Codes and Classifications [file jamanetwopen-e2326296-s001.pdf]

Supplemental Online Content

Brenner LA, Forster JE, Gradus JL, et al. Associations of military-related traumatic brain injury with new-onset mental health conditions and suicide risk. *JAMA Netw Open*. 2023;6(7):e2326296. doi:10.1001/jamanetworkopen.2023.26296

eMethods.
eTable 1. TBI *ICD-9* and *ICD-10* Codes
eTable 2. Mental Health *ICD-9* and *ICD-10* Codes and Classifications

This supplemental material has been provided by the authors to give readers additional information about their work.

© 2023 Brenner LA et al. *JAMA Network Open.*

eMethods

Matching Process

To determine pre-, post-, and new onset mental health diagnoses for all soldiers, those without a history of traumatic brain injury (TBI) had to be assigned a date such that mental health diagnoses documented prior to this date would be considered pre-, those documented after would be considered post-, and those documented after, without any documentation prior, would be considered new onset. We used the following procedure to assign a match date to those without TBI. First, there was variability in the amount of available Military Health System data prior to the end of index deployment across soldiers, and these data were only available dating back to 2005. This availability is likely to be associated with the probability of identifying TBI and/or the first documentation of mental health conditions. As the first step in matching, we categorized availability of pre-deployment data into the following: 0, 1-30, 31-60, 61-90, 91-180, 181-365, 366-730, 731-1095 and >1095 days of available pre-deployment data for each soldier.

Additionally, soldiers were placed into three categories based on the Fiscal Year (FY) of their return from index deployment: 2008-2009, 2010-2011, 2012-2014, as this is related to the amount of time they were followed for our outcome of interest (i.e., suicide, available through 2018). Soldiers without a history of TBI were then matched to an individual with a history of TBI within each FY return by days of available data stratum (total of 9 x 3=27 strata). Within each stratum, the number of soldiers without TBI was always greater than the number with TBI and as such, we determined how many of those without TBI would need to be matched per each individual with TBI, and they were subsequently randomly assigned. Once the matches were made, the individual without a history of TBI was then assigned a match date based on the number of days between the TBI diagnosis date and the return from index deployment date for the matched individual with a history of TBI. For example, if the non-TBI soldier was matched to a soldier with a history of TBI and their TBI diagnosis occurred 4 days after their return from index deployment, the non-TBI soldier would be assigned a match date that was 4 days after the non-TBI soldier's return from index deployment. Thirty-eight soldiers without a history of TBI were removed because they could not be matched using this process, resulting in a final analytic cohort of 860,892.

© 2023 Brenner LA et al. *JAMA Network Open.*

Mediation Models
The following Directed Acyclic Graph (DAG) illustrates the assumed models.

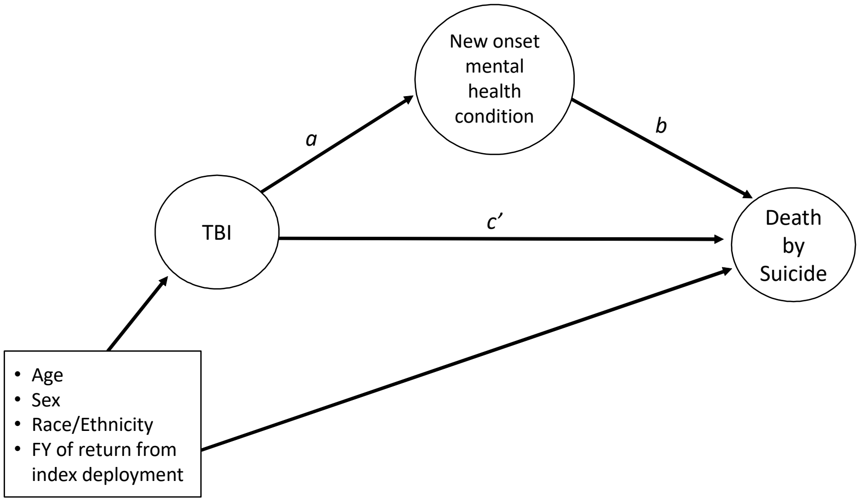

In this DAG, TBI is the **exposure**, each new onset mental health condition is the **mediator** (one set of models for each of the six set of conditions), time to death by suicide is the **outcome** and there are 4 **covariates**: age (18-24, 25-29, 30-34, 35-39, 40+), sex assigned in the medical record, race/ethnicity (American Indian/Alaskan Native (AIAN), Asian American or Pacific Islander (AAPI), Black non-Hispanic (BNH), White non-Hispanic, Hispanic, Other/Unknown), and FY of return from index deployment categorized as FY2008-09, FY2010-11, and FY2012-14. The direct effect of TBI on suicide is represented by the arrow leading directly from TBI to death by suicide (*c'*), and the indirect effect of TBI on suicide, mediated by new onset mental health conditions, is represented by the arrow leading from TBI to new onset mental health condition and the subsequent arrow leading to death by suicide (the product of coefficients, *a***b*). As the covariates are associated with both TBI and death by suicide, arrows extend from these to both the exposure and the outcome. Two models per new onset mental health condition category were fit to determine both the direct and indirect effect of TBI on death by suicide. The first is a Poisson regression with robust error variance that estimates the effect of TBI on new onset mental health conditions (coefficient *a*; arrow leading from TBI to new onset mental health condition) and includes the covariates indicated in the DAG. The model is as described in Zou, 2004.[1] This model can be written as:

(1) $\log(\text{new onset mental health condition}) = \beta_0 + \beta_1(\text{TBI}) + \beta_2(\text{covariate 1}) + \ldots + \beta_{13}(\text{covariate 12})$.

In this model a Poisson distribution is assumed for the data, but they are distributed binomially, and as such this model uses a sandwich estimator for the variance, which provides the necessary correction.[1] The risk of a new onset mental health condition for those with TBI relative to those without TBI (relative risk) is estimated as $\exp(\hat{\beta}_1)$, which is the *a* coefficient in the DAG above.

The second model fit is an Accelerated Failure Time (AFT; time to event) model that estimates the relative speed at which the event (death by suicide) occurs for an individual with one set of characteristics relative to an individual with another set of characteristics. These are parametric

© 2023 Brenner LA et al. *JAMA Network Open.*

models that assume a distribution for the error term and the event times. The AFT model with the best fit to the data was a log-logistic model. This model can be written as:

(2) $T = \exp\{\beta_0 + \beta_1(\text{TBI}) + \beta_2\,(\text{new onset mental health condition}) + \beta_3(\text{covariate 1}) + \ldots + \beta_{14}\,(\text{covariate 12}) + \sigma\varepsilon\}$

where T is the event time variable and σ is a scale parameter for ε, such that the survival times follow a log-logistic distribution. The ε term is a random variable (error term) that follows a logistic distribution. The *b* coefficient for the DAG above is estimated as $\exp(\hat{\beta}_2)$ from this model, i.e., the estimated effect of the mediator on suicide. This model also gives the direct effect of TBI on suicide and is estimated as $\exp(\hat{\beta}_1)$. All point estimates reported are from models (1) and (2) above that were fit to the analytic dataset, as are all 95% confidence intervals (CIs) except for those associated with the indirect effect as we are taking the product of coefficients from two separate models. As such, the 95% CIs for the indirect effects were calculated using 1000 bootstrap datasets for each new onset mental health condition (N equal to the original dataset and observations sampled from the original dataset with replacement). For each dataset, an AFT model with the mediator and covariates was fit and a Poisson regression was fit including covariates. The product of coefficients was calculated for each set of models resulting in 1000 indirect effect estimates from which the 2.5% and 97.5% estimates were taken as the lower and upper bounds of the 95% CI for the indirect effect.

Supplemental References

1. Zou G. A modified poisson regression approach to prospective studies with binary data. *Am J Epidemiol*. Apr 1 2004;159(7):702-6. doi:10.1093/aje/kwh090

© 2023 Brenner LA et al. *JAMA Network Open.*

**eTable 1:** TBI *ICD-9* and *ICD-10* Codes		
**ICD Code**	**Code Type**	**Description**
310.2	ICD9	Postconcussion syndrome
800.02	ICD9	Closed fracture of vault of skull without mention of intracranial injury, with brief [less than one hour] loss of consciousness
800.03	ICD9	Closed fracture of vault of skull without mention of intracranial injury, with moderate [1-24 hours] loss of consciousness
800.04	ICD9	Closed fracture of vault of skull without mention of intracranial injury, with prolonged [more than 24 hours] loss of consciousness and return to pre-existing conscious level
800.05	ICD9	Closed fracture of vault of skull without mention of intracranial injury, with prolonged [more than 24 hours] loss of consciousness, without return to pre-existing conscious level
800.06	ICD9	Closed fracture of vault of skull without mention of intracranial injury, with loss of consciousness of unspecified duration
800.09	ICD9	Closed fracture of vault of skull without mention of intracranial injury, with concussion, unspecified
800.1	ICD9	Closed fracture of vault of skull with cerebral laceration and contusion
800.10	ICD9	Closed fracture of vault of skull with cerebral laceration and contusion, unspecified state of consciousness
800.11	ICD9	Closed fracture of vault of skull with cerebral laceration and contusion, with no loss of consciousness
800.12	ICD9	Closed fracture of vault of skull with cerebral laceration and contusion, with brief [less than one hour] loss of consciousness
800.13	ICD9	Closed fracture of vault of skull with cerebral laceration and contusion, with moderate [1-24 hours] loss of consciousness
800.14	ICD9	Closed fracture of vault of skull with cerebral laceration and contusion, with prolonged [more than 24 hours] loss of consciousness and return to pre-existing conscious level
800.15	ICD9	Closed fracture of vault of skull with cerebral laceration and contusion, with prolonged [more than 24 hours] loss of consciousness, without return to pre-existing conscious level
800.16	ICD9	Closed fracture of vault of skull with cerebral laceration and contusion, with loss of consciousness of unspecified duration
800.19	ICD9	Closed fracture of vault of skull with cerebral laceration and contusion, with concussion, unspecified
800.2	ICD9	Closed fracture of vault of skull with subarachnoid subdural and extradural hemorrhage
800.20	ICD9	Closed fracture of vault of skull with subarachnoid, subdural, and extradural hemorrhage, unspecified state of consciousness
800.21	ICD9	Closed fracture of vault of skull with subarachnoid, subdural, and extradural hemorrhage, with no loss of consciousness
800.22	ICD9	Closed fracture of vault of skull with subarachnoid, subdural, and extradural hemorrhage, with brief [less than one hour] loss of consciousness
800.23	ICD9	Closed fracture of vault of skull with subarachnoid, subdural, and extradural hemorrhage, with moderate [1-24 hours] loss of consciousness

© 2023 Brenner LA et al. *JAMA Network Open.*

800.24	ICD9	Closed fracture of vault of skull with subarachnoid, subdural, and extradural hemorrhage, with prolonged [more than 24 hours] loss of consciousness and return to pre-existing conscious level
800.25	ICD9	Closed fracture of vault of skull with subarachnoid, subdural, and extradural hemorrhage, with prolonged [more than 24 hours] loss of consciousness, without return to pre-existing conscious level
800.26	ICD9	Closed fracture of vault of skull with subarachnoid, subdural, and extradural hemorrhage, with loss of consciousness of unspecified duration
800.29	ICD9	Closed fracture of vault of skull with subarachnoid, subdural, and extradural hemorrhage, with concussion, unspecified
800.3	ICD9	Closed fracture of vault of skull with other and unspecified intracranial hemorrhage
800.30	ICD9	Closed fracture of vault of skull with other and unspecified intracranial hemorrhage, unspecified state of consciousness
800.31	ICD9	Closed fracture of vault of skull with other and unspecified intracranial hemorrhage, with no loss of consciousness
800.32	ICD9	Closed fracture of vault of skull with other and unspecified intracranial hemorrhage, with brief [less than one hour] loss of consciousness
800.33	ICD9	Closed fracture of vault of skull with other and unspecified intracranial hemorrhage, with moderate [1-24 hours] loss of consciousness
800.34	ICD9	Closed fracture of vault of skull with other and unspecified intracranial hemorrhage, with prolonged [more than 24 hours] loss of consciousness and return to pre-existing conscious level
800.35	ICD9	Closed fracture of vault of skull with other and unspecified intracranial hemorrhage, with prolonged [more than 24 hours] loss of consciousness, without return to pre-existing conscious level
800.36	ICD9	Closed fracture of vault of skull with other and unspecified intracranial hemorrhage, with loss of consciousness of unspecified duration
800.39	ICD9	Closed fracture of vault of skull with other and unspecified intracranial hemorrhage, with concussion, unspecified
800.4	ICD9	Closed fracture of vault of skull with intracranial injury of other and unspecified nature
800.40	ICD9	Closed fracture of vault of skull with intracranial injury of other and unspecified nature, unspecified state of consciousness
800.41	ICD9	Closed fracture of vault of skull with intracranial injury of other and unspecified nature, with no loss of consciousness
800.42	ICD9	Closed fracture of vault of skull with intracranial injury of other and unspecified nature, with brief [less than one hour] loss of consciousness
800.43	ICD9	Closed fracture of vault of skull with intracranial injury of other and unspecified nature, with moderate [1-24 hours] loss of consciousness
800.44	ICD9	Closed fracture of vault of skull with intracranial injury of other and unspecified nature, with prolonged [more than 24 hours] loss of consciousness and return to pre-existing conscious level
800.45	ICD9	Closed fracture of vault of skull with intracranial injury of other and unspecified nature, with prolonged [more than 24 hours] loss of consciousness, without return to pre-existing conscious level

© 2023 Brenner LA et al. *JAMA Network Open.*

800.46	ICD9	Closed fracture of vault of skull with intracranial injury of other and unspecified nature, with loss of consciousness of unspecified duration
800.49	ICD9	Closed fracture of vault of skull with intracranial injury of other and unspecified nature, with concussion, unspecified
800.52	ICD9	Open fracture of vault of skull without mention of intracranial injury, with brief [less than one hour] loss of consciousness
800.53	ICD9	Open fracture of vault of skull without mention of intracranial injury, with moderate [1-24 hours] loss of consciousness
800.54	ICD9	Open fracture of vault of skull without mention of intracranial injury, with prolonged [more than 24 hours] loss of consciousness and return to pre-existing conscious level
800.55	ICD9	Open fracture of vault of skull without mention of intracranial injury, with prolonged [more than 24 hours] loss of consciousness, without return to pre-existing conscious level
800.56	ICD9	Open fracture of vault of skull without mention of intracranial injury, with loss of consciousness of unspecified duration
800.59	ICD9	Open fracture of vault of skull without mention of intracranial injury, with concussion, unspecified
800.6	ICD9	Open fracture of vault of skull with cerebral laceration and contusion
800.60	ICD9	Open fracture of vault of skull with cerebral laceration and contusion, unspecified state of consciousness
800.61	ICD9	Open fracture of vault of skull with cerebral laceration and contusion, with no loss of consciousness
800.62	ICD9	Open fracture of vault of skull with cerebral laceration and contusion, with brief [less than one hour] loss of consciousness
800.63	ICD9	Open fracture of vault of skull with cerebral laceration and contusion, with moderate [1-24 hours] loss of consciousness
800.64	ICD9	Open fracture of vault of skull with cerebral laceration and contusion, with prolonged [more than 24 hours] loss of consciousness and return to pre-existing conscious level
800.65	ICD9	Open fracture of vault of skull with cerebral laceration and contusion, with prolonged [more than 24 hours] loss of consciousness, without return to pre-existing conscious level
800.66	ICD9	Open fracture of vault of skull with cerebral laceration and contusion, with loss of consciousness of unspecified duration
800.69	ICD9	Open fracture of vault of skull with cerebral laceration and contusion, with concussion, unspecified
800.7	ICD9	Open fracture of vault of skull with subarachnoid subdural and extradural hemorrhage
800.70	ICD9	Open fracture of vault of skull with subarachnoid, subdural, and extradural hemorrhage, unspecified state of consciousness
800.71	ICD9	Open fracture of vault of skull with subarachnoid, subdural, and extradural hemorrhage, with no loss of consciousness
800.72	ICD9	Open fracture of vault of skull with subarachnoid, subdural, and extradural hemorrhage, with brief [less than one hour] loss of consciousness
800.73	ICD9	Open fracture of vault of skull with subarachnoid, subdural, and extradural hemorrhage, with moderate [1-24 hours] loss of consciousness

© 2023 Brenner LA et al. *JAMA Network Open.*

800.74	ICD9	Open fracture of vault of skull with subarachnoid, subdural, and extradural hemorrhage, with prolonged [more than 24 hours] loss of consciousness and return to pre-existing conscious level
800.75	ICD9	Open fracture of vault of skull with subarachnoid, subdural, and extradural hemorrhage, with prolonged [more than 24 hours] loss of consciousness, without return to pre-existing conscious level
800.76	ICD9	Open fracture of vault of skull with subarachnoid, subdural, and extradural hemorrhage, with loss of consciousness of unspecified duration
800.79	ICD9	Open fracture of vault of skull with subarachnoid, subdural, and extradural hemorrhage, with concussion, unspecified
800.8	ICD9	Open fracture of vault of skull with other and unspecified intracranial hemorrhage
800.80	ICD9	Open fracture of vault of skull with other and unspecified intracranial hemorrhage, unspecified state of consciousness
800.81	ICD9	Open fracture of vault of skull with other and unspecified intracranial hemorrhage, with no loss of consciousness
800.82	ICD9	Open fracture of vault of skull with other and unspecified intracranial hemorrhage, with brief [less than one hour] loss of consciousness
800.83	ICD9	Open fracture of vault of skull with other and unspecified intracranial hemorrhage, with moderate [1-24 hours] loss of consciousness
800.84	ICD9	Open fracture of vault of skull with other and unspecified intracranial hemorrhage, with prolonged [more than 24 hours] loss of consciousness and return to pre-existing conscious level
800.85	ICD9	Open fracture of vault of skull with other and unspecified intracranial hemorrhage, with prolonged [more than 24 hours] loss of consciousness, without return to pre-existing conscious level
800.86	ICD9	Open fracture of vault of skull with other and unspecified intracranial hemorrhage, with loss of consciousness of unspecified duration
800.89	ICD9	Open fracture of vault of skull with other and unspecified intracranial hemorrhage, with concussion, unspecified
800.9	ICD9	Open fracture of vault of skull with intracranial injury of other and unspecified nature
800.90	ICD9	Open fracture of vault of skull with intracranial injury of other and unspecified nature, unspecified state of consciousness
800.91	ICD9	Open fracture of vault of skull with intracranial injury of other and unspecified nature, with no loss of consciousness
800.92	ICD9	Open fracture of vault of skull with intracranial injury of other and unspecified nature, with brief [less than one hour] loss of consciousness
800.93	ICD9	Open fracture of vault of skull with intracranial injury of other and unspecified nature, with moderate [1-24 hours] loss of consciousness
800.94	ICD9	Open fracture of vault of skull with intracranial injury of other and unspecified nature, with prolonged [more than 24 hours] loss of consciousness and return to pre-existing conscious level
800.95	ICD9	Open fracture of vault of skull with intracranial injury of other and unspecified nature, with prolonged [more than 24 hours] loss of consciousness, without return to pre-existing conscious level
800.96	ICD9	Open fracture of vault of skull with intracranial injury of other and unspecified nature, with loss of consciousness of unspecified duration

© 2023 Brenner LA et al. *JAMA Network Open.*

800.99	ICD9	Open fracture of vault of skull with intracranial injury of other and unspecified nature, with concussion, unspecified
801.02	ICD9	Closed fracture of base of skull without mention of intra cranial injury, with brief [less than one hour] loss of consciousness
801.03	ICD9	Closed fracture of base of skull without mention of intra cranial injury, with moderate [1-24 hours] loss of consciousness
801.04	ICD9	Closed fracture of base of skull without mention of intra cranial injury, with prolonged [more than 24 hours] loss of consciousness and return to pre-existing conscious level
801.05	ICD9	Closed fracture of base of skull without mention of intra cranial injury, with prolonged [more than 24 hours] loss of consciousness, without return to pre-existing conscious level
801.06	ICD9	Closed fracture of base of skull without mention of intra cranial injury, with loss of consciousness of unspecified duration
801.09	ICD9	Closed fracture of base of skull without mention of intra cranial injury, with concussion, unspecified
801.1	ICD9	Closed fracture of base of skull with cerebral laceration and contusion
801.10	ICD9	Closed fracture of base of skull with cerebral laceration and contusion, unspecified state of consciousness
801.11	ICD9	Closed fracture of base of skull with cerebral laceration and contusion, with no loss of consciousness
801.12	ICD9	Closed fracture of base of skull with cerebral laceration and contusion, with brief [less than one hour] loss of consciousness
801.13	ICD9	Closed fracture of base of skull with cerebral laceration and contusion, with moderate [1-24 hours] loss of consciousness
801.14	ICD9	Closed fracture of base of skull with cerebral laceration and contusion, with prolonged [more than 24 hours] loss of consciousness and return to pre-existing conscious level
801.15	ICD9	Closed fracture of base of skull with cerebral laceration and contusion, with prolonged [more than 24 hours] loss of consciousness, without return to pre-existing conscious level
801.16	ICD9	Closed fracture of base of skull with cerebral laceration and contusion, with loss of consciousness of unspecified duration
801.19	ICD9	Closed fracture of base of skull with cerebral laceration and contusion, with concussion, unspecified
801.2	ICD9	Closed fracture of base of skull with subarachnoid subdural and extradural hemorrhage
801.20	ICD9	Closed fracture of base of skull with subarachnoid, subdural, and extradural hemorrhage, unspecified state of consciousness
801.21	ICD9	Closed fracture of base of skull with subarachnoid, subdural, and extradural hemorrhage, with no loss of consciousness
801.22	ICD9	Closed fracture of base of skull with subarachnoid, subdural, and extradural hemorrhage, with brief [less than one hour] loss of consciousness
801.23	ICD9	Closed fracture of base of skull with subarachnoid, subdural, and extradural hemorrhage, with moderate [1-24 hours] loss of consciousness
801.24	ICD9	Closed fracture of base of skull with subarachnoid, subdural, and extradural hemorrhage, with prolonged [more than 24 hours] loss of consciousness and return to pre-existing conscious level

© 2023 Brenner LA et al. *JAMA Network Open.*

801.25	ICD9	Closed fracture of base of skull with subarachnoid, subdural, and extradural hemorrhage, with prolonged [more than 24 hours] loss of consciousness, without return to pre-existing conscious level
801.26	ICD9	Closed fracture of base of skull with subarachnoid, subdural, and extradural hemorrhage, with loss of consciousness of unspecified duration
801.29	ICD9	Closed fracture of base of skull with subarachnoid, subdural, and extradural hemorrhage, with concussion, unspecified
801.3	ICD9	Closed fracture of base of skull with other and unspecified intracranial hemorrhage
801.30	ICD9	Closed fracture of base of skull with other and unspecified intracranial hemorrhage, unspecified state of consciousness
801.31	ICD9	Closed fracture of base of skull with other and unspecified intracranial hemorrhage, with no loss of consciousness
801.32	ICD9	Closed fracture of base of skull with other and unspecified intracranial hemorrhage, with brief [less than one hour] loss of consciousness
801.33	ICD9	Closed fracture of base of skull with other and unspecified intracranial hemorrhage, with moderate [1-24 hours] loss of consciousness
801.34	ICD9	Closed fracture of base of skull with other and unspecified intracranial hemorrhage, with prolonged [more than 24 hours] loss of consciousness and return to pre-existing conscious level
801.35	ICD9	Closed fracture of base of skull with other and unspecified intracranial hemorrhage, with prolonged [more than 24 hours] loss of consciousness, without return to pre-existing conscious level
801.36	ICD9	Closed fracture of base of skull with other and unspecified intracranial hemorrhage, with loss of consciousness of unspecified duration
801.39	ICD9	Closed fracture of base of skull with other and unspecified intracranial hemorrhage, with concussion, unspecified
801.4	ICD9	Closed fracture of base of skull with intracranial injury of other and unspecified nature
801.40	ICD9	Closed fracture of base of skull with intracranial injury of other and unspecified nature, unspecified state of consciousness
801.41	ICD9	Closed fracture of base of skull with intracranial injury of other and unspecified nature, with no loss of consciousness
801.42	ICD9	Closed fracture of base of skull with intracranial injury of other and unspecified nature, with brief [less than one hour] loss of consciousness
801.43	ICD9	Closed fracture of base of skull with intracranial injury of other and unspecified nature, with moderate [1-24 hours] loss of consciousness
801.44	ICD9	Closed fracture of base of skull with intracranial injury of other and unspecified nature, with prolonged [more than 24 hours) loss of consciousness and return to pre-existing conscious level
801.45	ICD9	Closed fracture of base of skull with intracranial injury of other and unspecified nature, with prolonged [more than 24 hours] loss of consciousness, without return to pre-existing conscious level
801.46	ICD9	Closed fracture of base of skull with intracranial injury of other and unspecified nature, with loss of consciousness of unspecified duration

© 2023 Brenner LA et al. *JAMA Network Open.*

801.49	ICD9	Closed fracture of base of skull with intracranial injury of other and unspecified nature, with concussion, unspecified
801.52	ICD9	Open fracture of base of skull without mention of intracranial injury, with brief [less than one hour] loss of consciousness
801.53	ICD9	Open fracture of base of skull without mention of intracranial injury, with moderate [1-24 hours] loss of consciousness
801.54	ICD9	Open fracture of base of skull without mention of intracranial injury, with prolonged [more than 24 hours] loss of consciousness and return to pre-existing conscious level
801.55	ICD9	Open fracture of base of skull without mention of intracranial injury, with prolonged [more than 24 hours] loss of consciousness, without return to pre-existing conscious level
801.56	ICD9	Open fracture of base of skull without mention of intracranial injury, with loss of consciousness of unspecified duration
801.59	ICD9	Open fracture of base of skull without mention of intracranial injury, with concussion, unspecified
801.6	ICD9	Open fracture of base of skull with cerebral laceration and contusion
801.60	ICD9	Open fracture of base of skull with cerebral laceration and contusion, unspecified state of consciousness
801.61	ICD9	Open fracture of base of skull with cerebral laceration and contusion, with no loss of consciousness
801.62	ICD9	Open fracture of base of skull with cerebral laceration and contusion, with brief [less than one hour] loss of consciousness
801.63	ICD9	Open fracture of base of skull with cerebral laceration and contusion, with moderate [1-24 hours] loss of consciousness
801.64	ICD9	Open fracture of base of skull with cerebral laceration and contusion, with prolonged [more than 24 hours] loss of consciousness and return to pre-existing conscious level
801.65	ICD9	Open fracture of base of skull with cerebral laceration and contusion, with prolonged [more than 24 hours] loss of consciousness, without return to pre-existing conscious level
801.66	ICD9	Open fracture of base of skull with cerebral laceration and contusion, with loss of consciousness of unspecified duration
801.69	ICD9	Open fracture of base of skull with cerebral laceration and contusion, with concussion, unspecified
801.7	ICD9	Open fracture of base of skull with subarachnoid subdural and extradural hemorrhage
801.70	ICD9	Open fracture of base of skull with subarachnoid, subdural, and extradural hemorrhage, unspecified state of consciousness
801.71	ICD9	Open fracture of base of skull with subarachnoid, subdural, and extradural hemorrhage, with no loss of consciousness
801.72	ICD9	Open fracture of base of skull with subarachnoid, subdural, and extradural hemorrhage, with brief [less than one hour] loss of consciousness
801.73	ICD9	Open fracture of base of skull with subarachnoid, subdural, and extradural hemorrhage, with moderate [1-24 hours] loss of consciousness
801.74	ICD9	Open fracture of base of skull with subarachnoid, subdural, and extradural hemorrhage, with prolonged [more than 24 hours] loss of consciousness and return to pre-existing conscious level

© 2023 Brenner LA et al. *JAMA Network Open.*

801.75	ICD9	Open fracture of base of skull with subarachnoid, subdural, and extradural hemorrhage, with prolonged [more than 24 hours] loss of consciousness, without return to pre-existing conscious level
801.76	ICD9	Open fracture of base of skull with subarachnoid, subdural, and extradural hemorrhage, with loss of consciousness of unspecified duration
801.79	ICD9	Open fracture of base of skull with subarachnoid, subdural, and extradural hemorrhage, with concussion, unspecified
801.8	ICD9	Open fracture of base of skull with other and unspecified intracranial hemorrhage
801.80	ICD9	Open fracture of base of skull with other and unspecified intracranial hemorrhage, unspecified state of consciousness
801.81	ICD9	Open fracture of base of skull with other and unspecified intracranial hemorrhage, with no loss of consciousness
801.82	ICD9	Open fracture of base of skull with other and unspecified intracranial hemorrhage, with brief [less than one hour] loss of consciousness
801.83	ICD9	Open fracture of base of skull with other and unspecified intracranial hemorrhage, with moderate [1-24 hours] loss of consciousness
801.84	ICD9	Open fracture of base of skull with other and unspecified intracranial hemorrhage, with prolonged [more than 24 hours] loss of consciousness and return to pre-existing conscious level
801.85	ICD9	Open fracture of base of skull with other and unspecified intracranial hemorrhage, with prolonged [more than 24 hours] loss of consciousness, without return to pre-existing conscious level
801.86	ICD9	Open fracture of base of skull with other and unspecified intracranial hemorrhage, with loss of consciousness of unspecified duration
801.89	ICD9	Open fracture of base of skull with other and unspecified intracranial hemorrhage, with concussion, unspecified
801.9	ICD9	Open fracture of base of skull with intracranial injury of other and unspecified nature
801.90	ICD9	Open fracture of base of skull with intracranial injury of other and unspecified nature, unspecified state of consciousness
801.91	ICD9	Open fracture of base of skull with intracranial injury of other and unspecified nature, with no loss of consciousness
801.92	ICD9	Open fracture of base of skull with intracranial injury of other and unspecified nature, with brief [less than one hour] loss of consciousness
801.93	ICD9	Open fracture of base of skull with intracranial injury of other and unspecified nature, with moderate [1-24 hours] loss of consciousness
801.94	ICD9	Open fracture of base of skull with intracranial injury of other and unspecified nature, with prolonged [more than 24 hours] loss of consciousness and return to pre-existing conscious level
801.95	ICD9	Open fracture of base of skull with intracranial injury of other and unspecified nature, with prolonged [more than 24 hours] loss of consciousness, without return to pre-existing conscious level
801.96	ICD9	Open fracture of base of skull with intracranial injury of other and unspecified nature, with loss of consciousness of unspecified duration
801.99	ICD9	Open fracture of base of skull with intracranial injury of other and unspecified nature, with concussion, unspecified

© 2023 Brenner LA et al. *JAMA Network Open.*

803.02	ICD9	Other closed skull fracture without mention of intracranial injury, with brief [less than one hour] loss of consciousness
803.03	ICD9	Other closed skull fracture without mention of intracranial injury, with moderate [1-24 hours] loss of consciousness
803.04	ICD9	Other closed skull fracture without mention of intracranial injury, with prolonged [more than 24 hours] loss of consciousness and return to pre-existing conscious level
803.05	ICD9	Other closed skull fracture without mention of intracranial injury, with prolonged [more than 24 hours] loss of consciousness, without return to pre-existing conscious level
803.06	ICD9	Other closed skull fracture without mention of intracranial injury, with loss of consciousness of unspecified duration
803.09	ICD9	Other closed skull fracture without mention of intracranial injury, with concussion, unspecified
803.1	ICD9	Other closed skull fracture with cerebral laceration and contusion
803.10	ICD9	Other closed skull fracture with cerebral laceration and contusion, unspecified state of consciousness
803.11	ICD9	Other closed skull fracture with cerebral laceration and contusion, with no loss of consciousness
803.12	ICD9	Other closed skull fracture with cerebral laceration and contusion, with brief [less than one hour] loss of consciousness
803.13	ICD9	Other closed skull fracture with cerebral laceration and contusion, with moderate [1-24 hours] loss of consciousness
803.14	ICD9	Other closed skull fracture with cerebral laceration and contusion, with prolonged [more than 24 hours] loss of consciousness and return to pre-existing conscious level
803.15	ICD9	Other closed skull fracture with cerebral laceration and contusion, with prolonged [more than 24 hours] loss of consciousness, without return to pre-existing conscious level
803.16	ICD9	Other closed skull fracture with cerebral laceration and contusion, with loss of consciousness of unspecified duration
803.19	ICD9	Other closed skull fracture with cerebral laceration and contusion, with concussion, unspecified
803.2	ICD9	Other closed skull fracture with subarachnoid subdural and extradural hemorrhage
803.20	ICD9	Other closed skull fracture with subarachnoid, subdural, and extradural hemorrhage, unspecified state of consciousness
803.21	ICD9	Other closed skull fracture with subarachnoid, subdural, and extradural hemorrhage, with no loss of consciousness
803.22	ICD9	Other closed skull fracture with subarachnoid, subdural, and extradural hemorrhage, with brief [less than one hour] loss of consciousness
803.23	ICD9	Other closed skull fracture with subarachnoid, subdural, and extradural hemorrhage, with moderate [1-24 hours] loss of consciousness
803.24	ICD9	Other closed skull fracture with subarachnoid, subdural, and extradural hemorrhage, with prolonged [more than 24 hours] loss of consciousness and return to pre-existing conscious level
803.25	ICD9	Other closed skull fracture with subarachnoid, subdural, and extradural hemorrhage, with prolonged [more than 24 hours] loss of consciousness, without return to pre-existing conscious level

© 2023 Brenner LA et al. *JAMA Network Open.*

803.26	ICD9	Other closed skull fracture with subarachnoid, subdural, and extradural hemorrhage, with loss of consciousness of unspecified duration
803.29	ICD9	Other closed skull fracture with subarachnoid, subdural, and extradural hemorrhage, with concussion, unspecified
803.3	ICD9	Closed skull fracture with other and unspecified intracranial hemorrhage
803.30	ICD9	Other closed skull fracture with other and unspecified intracranial hemorrhage, unspecified state of unconsciousness
803.31	ICD9	Other closed skull fracture with other and unspecified intracranial hemorrhage, with no loss of consciousness
803.32	ICD9	Other closed skull fracture with other and unspecified intracranial hemorrhage, with brief [less than one hour] loss of consciousness
803.33	ICD9	Other closed skull fracture with other and unspecified intracranial hemorrhage, with moderate [1-24 hours] loss of consciousness
803.34	ICD9	Other closed skull fracture with other and unspecified intracranial hemorrhage, with prolonged [more than 24 hours] loss of consciousness and return to pre-existing conscious level
803.35	ICD9	Other closed skull fracture with other and unspecified intracranial hemorrhage, with prolonged [more than 24 hours] loss of consciousness, without return to pre-existing conscious level
803.36	ICD9	Other closed skull fracture with other and unspecified intracranial hemorrhage, with loss of consciousness of unspecified duration
803.39	ICD9	Other closed skull fracture with other and unspecified intracranial hemorrhage, with concussion, unspecified
803.4	ICD9	Other closed skull fracture with intracranial injury of other and unspecified nature
803.40	ICD9	Other closed skull fracture with intracranial injury of other and unspecified nature, unspecified state of consciousness
803.41	ICD9	Other closed skull fracture with intracranial injury of other and unspecified nature, with no loss of consciousness
803.42	ICD9	Other closed skull fracture with intracranial injury of other and unspecified nature, with brief [less than one hour] loss of consciousness
803.43	ICD9	Other closed skull fracture with intracranial injury of other and unspecified nature, with moderate [1-24 hours] loss of consciousness
803.44	ICD9	Other closed skull fracture with intracranial injury of other and unspecified nature, with prolonged [more than 24 hours] loss of consciousness and return to pre-existing conscious level
803.45	ICD9	Other closed skull fracture with intracranial injury of other and unspecified nature, with prolonged [more than 24 hours] loss of consciousness, without return to pre-existing conscious level
803.46	ICD9	Other closed skull fracture with intracranial injury of other and unspecified nature, with loss of consciousness of unspecified duration
803.49	ICD9	Other closed skull fracture with intracranial injury of other and unspecified nature, with concussion, unspecified
803.52	ICD9	Other open skull fracture without mention of intracranial injury, with brief [less than one hour] loss of consciousness
803.53	ICD9	Other open skull fracture without mention of intracranial injury, with moderate [1-24 hours] loss of consciousness

© 2023 Brenner LA et al. *JAMA Network Open.*

803.54	ICD9	Other open skull fracture without mention of intracranial injury, with prolonged [more than 24 hours] loss of consciousness and return to pre-existing conscious level
803.55	ICD9	Other open skull fracture without mention of intracranial injury, with prolonged [more than 24 hours] loss of consciousness, without return to pre-existing conscious level
803.56	ICD9	Other open skull fracture without mention of intracranial injury, with loss of consciousness of unspecified duration
803.59	ICD9	Other open skull fracture without mention of intracranial injury, with concussion, unspecified
803.6	ICD9	Other open skull fracture with cerebral laceration and contusion
803.60	ICD9	Other open skull fracture with cerebral laceration and contusion, unspecified state of consciousness
803.61	ICD9	Other open skull fracture with cerebral laceration and contusion, with no loss of consciousness
803.62	ICD9	Other open skull fracture with cerebral laceration and contusion, with brief [less than one hour] loss of consciousness
803.63	ICD9	Other open skull fracture with cerebral laceration and contusion, with moderate [1-24 hours] loss of consciousness
803.64	ICD9	Other open skull fracture with cerebral laceration and contusion, with prolonged [more than 24 hours] loss of consciousness and return to pre-existing conscious level
803.65	ICD9	Other open skull fracture with cerebral laceration and contusion, with prolonged [more than 24 hours] loss of consciousness, without return to pre-existing conscious level
803.66	ICD9	Other open skull fracture with cerebral laceration and contusion, with loss of consciousness of unspecified duration
803.69	ICD9	Other open skull fracture with cerebral laceration and contusion, with concussion, unspecified
803.7	ICD9	Other open skull fracture with subarachnoid subdural and extradural hemorrhage
803.70	ICD9	Other open skull fracture with subarachnoid, subdural, and extradural hemorrhage, unspecified state of consciousness
803.71	ICD9	Other open skull fracture with subarachnoid, subdural, and extradural hemorrhage, with no loss of consciousness
803.72	ICD9	Other open skull fracture with subarachnoid, subdural, and extradural hemorrhage, with brief [less than one hour] loss of consciousness
803.73	ICD9	Other open skull fracture with subarachnoid, subdural, and extradural hemorrhage, with moderate [1-24 hours] loss of consciousness
803.74	ICD9	Other open skull fracture with subarachnoid, subdural, and extradural hemorrhage, with prolonged [more than 24 hours] loss of consciousness and return to pre-existing conscious level
803.75	ICD9	Other open skull fracture with subarachnoid, subdural, and extradural hemorrhage, with prolonged [more than 24 hours] loss of consciousness, without return to pre-existing conscious level
803.76	ICD9	Other open skull fracture with subarachnoid, subdural, and extradural hemorrhage, with loss of consciousness of unspecified duration
803.79	ICD9	Other open skull fracture with subarachnoid, subdural, and extradural hemorrhage, with concussion, unspecified

© 2023 Brenner LA et al. *JAMA Network Open.*

803.8	ICD9	Other open skull fracture with other and unspecified intracranial hemorrhage
803.80	ICD9	Other open skull fracture with other and unspecified intracranial hemorrhage, unspecified state of consciousness
803.81	ICD9	Other open skull fracture with other and unspecified intracranial hemorrhage, with no loss of consciousness
803.82	ICD9	Other open skull fracture with other and unspecified intracranial hemorrhage, with brief [less than one hour] loss of consciousness
803.83	ICD9	Other open skull fracture with other and unspecified intracranial hemorrhage, with moderate [1-24 hours] loss of consciousness
803.84	ICD9	Other open skull fracture with other and unspecified intracranial hemorrhage, with prolonged [more than 24 hours] loss of consciousness and return to pre-existing conscious level
803.85	ICD9	Other open skull fracture with other and unspecified intracranial hemorrhage, with prolonged [more than 24 hours] loss of consciousness, without return to pre-existing conscious level
803.86	ICD9	Other open skull fracture with other and unspecified intracranial hemorrhage, with loss of consciousness of unspecified duration
803.89	ICD9	Other open skull fracture with other and unspecified intracranial hemorrhage, with concussion, unspecified
803.9	ICD9	Other open skull fracture with intracranial injury of other and unspecified nature
803.90	ICD9	Other open skull fracture with intracranial injury of other and unspecified nature, unspecified state of consciousness
803.91	ICD9	Other open skull fracture with intracranial injury of other and unspecified nature, with no loss of consciousness
803.92	ICD9	Other open skull fracture with intracranial injury of other and unspecified nature, with brief [less than one hour] loss of consciousness
803.93	ICD9	Other open skull fracture with intracranial injury of other and unspecified nature, with moderate [1-24 hours] loss of consciousness
803.94	ICD9	Other open skull fracture with intracranial injury of other and unspecified nature, with prolonged [more than 24 hours] loss of consciousness and return to pre-existing conscious level
803.95	ICD9	Other open skull fracture with intracranial injury of other and unspecified nature, with prolonged [more than 24 hours] loss of consciousness, without return to pre-existing conscious level
803.96	ICD9	Other open skull fracture with intracranial injury of other and unspecified nature, with loss of consciousness of unspecified duration
803.99	ICD9	Other open skull fracture with intracranial injury of other and unspecified nature, with concussion, unspecified
804.02	ICD9	Closed fractures involving skull or face with other bones, without mention of intracranial injury, with brief [less than one hour] loss of consciousness
804.03	ICD9	Closed fractures involving skull or face with other bones, without mention of intracranial injury, with moderate [1-24 hours] loss of consciousness
804.04	ICD9	Closed fractures involving skull or face with other bones, without mention or intracranial injury, with prolonged [more than 24 hours] loss of consciousness and return to pre-existing conscious level
804.05	ICD9	Closed fractures involving skull of face with other bones, without mention of intracranial injury, with prolonged [more than 24 hours] loss of consciousness, without return to pre-existing conscious level

© 2023 Brenner LA et al. *JAMA Network Open.*

804.06	ICD9	Closed fractures involving skull of face with other bones, without mention of intracranial injury, with loss of consciousness of unspecified duration
804.09	ICD9	Closed fractures involving skull of face with other bones, without mention of intracranial injury, with concussion, unspecified
804.1	ICD9	Closed fractures involving skull or face with other bones with cerebral laceration and contusion
804.10	ICD9	Closed fractures involving skull or face with other bones, with cerebral laceration and contusion, unspecified state of consciousness
804.11	ICD9	Closed fractures involving skull or face with other bones, with cerebral laceration and contusion, with no loss of consciousness
804.12	ICD9	Closed fractures involving skull or face with other bones, with cerebral laceration and contusion, with brief [less than one hour] loss of consciousness
804.13	ICD9	Closed fractures involving skull or face with other bones, with cerebral laceration and contusion, with moderate [1-24 hours] loss of consciousness
804.14	ICD9	Closed fractures involving skull or face with other bones, with cerebral laceration and contusion, with prolonged [more than 24 hours] loss of consciousness and return to pre-existing conscious level
804.15	ICD9	Closed fractures involving skull or face with other bones, with cerebral laceration and contusion, with prolonged [more than 24 hours] loss of consciousness, without return to pre-existing conscious level
804.16	ICD9	Closed fractures involving skull or face with other bones, with cerebral laceration and contusion, with loss of consciousness of unspecified duration
804.19	ICD9	Closed fractures involving skull or face with other bones, with cerebral laceration and contusion, with concussion, unspecified
804.2	ICD9	Closed fractures involving skull or face with other bones with subarachnoid subdural and extradural hemorrhage
804.20	ICD9	Closed fractures involving skull or face with other bones with subarachnoid, subdural, and extradural hemorrhage, unspecified state of consciousness
804.21	ICD9	Closed fractures involving skull or face with other bones with subarachnoid, subdural, and extradural hemorrhage, with no loss of consciousness
804.22	ICD9	Closed fractures involving skull or face with other bones with subarachnoid, subdural, and extradural hemorrhage, with brief [less than one hour] loss of consciousness
804.23	ICD9	Closed fractures involving skull or face with other bones with subarachnoid, subdural, and extradural hemorrhage, with moderate [1-24 hours] loss of consciousness
804.24	ICD9	Closed fractures involving skull or face with other bones with subarachnoid, subdural, and extradural hemorrhage, with prolonged [more than 24 hours] loss of consciousness and return to pre-existing conscious level
804.25	ICD9	Closed fractures involving skull or face with other bones with subarachnoid, subdural, and extradural hemorrhage, with prolonged [more than 24 hours] loss of consciousness, without return to pre-existing conscious level
804.26	ICD9	Closed fractures involving skull or face with other bones with subarachnoid, subdural, and extradural hemorrhage, with loss of consciousness of unspecified duration

© 2023 Brenner LA et al. *JAMA Network Open.*

804.29	ICD9	Closed fractures involving skull or face with other bones with subarachnoid, subdural, and extradural hemorrhage, with concussion, unspecified
804.3	ICD9	Closed fractures involving skull or face with other bones with other and unspecified intracranial hemorrhage
804.30	ICD9	Closed fractures involving skull or face with other bones, with other and unspecified intracranial hemorrhage, unspecified state of consciousness
804.31	ICD9	Closed fractures involving skull or face with other bones, with other and unspecified intracranial hemorrhage, with no loss of consciousness
804.32	ICD9	Closed fractures involving skull or face with other bones, with other and unspecified intracranial hemorrhage, with brief [less than one hour] loss of consciousness
804.33	ICD9	Closed fractures involving skull or face with other bones, with other and unspecified intracranial hemorrhage, with moderate [1-24 hours] loss of consciousness
804.34	ICD9	Closed fractures involving skull or face with other bones, with other and unspecified intracranial hemorrhage, with prolonged [more than 24 hours] loss of consciousness and return to pre- existing conscious level
804.35	ICD9	Closed fractures involving skull or face with other bones, with other and unspecified intracranial hemorrhage, with prolonged [more than 24 hours] loss of consciousness, without return to pre-existing conscious level
804.36	ICD9	Closed fractures involving skull or face with other bones, with other and unspecified intracranial hemorrhage, with loss of consciousness of unspecified duration
804.39	ICD9	Closed fractures involving skull or face with other bones, with other and unspecified intracranial hemorrhage, with concussion, unspecified
804.4	ICD9	Closed fractures involving skull or face with other bones with intracranial injury of other and unspecified nature
804.40	ICD9	Closed fractures involving skull or face with other bones, with intracranial injury of other and unspecified nature, unspecified state of consciousness
804.41	ICD9	Closed fractures involving skull or face with other bones, with intracranial injury of other and unspecified nature, with no loss of consciousness
804.42	ICD9	Closed fractures involving skull or face with other bones, with intracranial injury of other and unspecified nature, with brief [less than one hour] loss of consciousness
804.43	ICD9	Closed fractures involving skull or face with other bones, with intracranial injury of other and unspecified nature, with moderate [1-24 hours] loss of consciousness
804.44	ICD9	Closed fractures involving skull or face with other bones, with intracranial injury of other and unspecified nature, with prolonged [more than 24 hours] loss of consciousness and return to pre-existing conscious level
804.45	ICD9	Closed fractures involving skull or face with other bones, with intracranial injury of other and unspecified nature, with prolonged [more than 24 hours] loss of consciousness, without return to pre-existing conscious level
804.46	ICD9	Closed fractures involving skull or face with other bones, with intracranial injury of other and unspecified nature, with loss of consciousness of unspecified duration
804.49	ICD9	Closed fractures involving skull or face with other bones, with intracranial injury of other and unspecified nature, with concussion, unspecified
804.52	ICD9	Open fractures involving skull or face with other bones, without mention of intracranial injury, with brief [less than one hour] loss of consciousness

© 2023 Brenner LA et al. *JAMA Network Open.*

804.53	ICD9	Open fractures involving skull or face with other bones, without mention of intracranial injury, with moderate [1-24 hours] loss of consciousness
804.54	ICD9	Open fractures involving skull or face with other bones, without mention of intracranial injury, with prolonged [more than 24 hours] loss of consciousness and return to pre-existing conscious level
804.55	ICD9	Open fractures involving skull or face with other bones, without mention of intracranial injury, with prolonged [more than 24 hours] loss of consciousness, without return to pre-existing conscious level
804.56	ICD9	Open fractures involving skull or face with other bones, without mention of intracranial injury, with loss of consciousness of unspecified duration
804.59	ICD9	Open fractures involving skull or face with other bones, without mention of intracranial injury, with concussion, unspecified
804.6	ICD9	Open fractures involving skull or face with other bones with cerebral laceration and contusion
804.60	ICD9	Open fractures involving skull or face with other bones, with cerebral laceration and contusion, unspecified state of consciousness
804.61	ICD9	Open fractures involving skull or face with other bones, with cerebral laceration and contusion, with no loss of consciousness
804.62	ICD9	Open fractures involving skull or face with other bones, with cerebral laceration and contusion, with brief [less than one hour] loss of consciousness
804.63	ICD9	Open fractures involving skull or face with other bones, with cerebral laceration and contusion, with moderate [1-24 hours] loss of consciousness
804.64	ICD9	Open fractures involving skull or face with other bones, with cerebral laceration and contusion, with prolonged [more than 24 hours] loss of consciousness and return to pre-existing conscious level
804.65	ICD9	Open fractures involving skull or face with other bones, with cerebral laceration and contusion, with prolonged [more than 24 hours] loss of consciousness, without return to pre-existing conscious level
804.66	ICD9	Open fractures involving skull or face with other bones, with cerebral laceration and contusion, with loss of consciousness of unspecified duration
804.69	ICD9	Open fractures involving skull or face with other bones, with cerebral laceration and contusion, with concussion, unspecified
804.7	ICD9	Open fractures involving skull or face with other bones with subarachnoid subdural and extradural hemorrhage
804.70	ICD9	Open fractures involving skull or face with other bones with subarachnoid, subdural, and extradural hemorrhage, unspecified state of consciousness
804.71	ICD9	Open fractures involving skull or face with other bones with subarachnoid, subdural, and extradural hemorrhage, with no loss of consciousness
804.72	ICD9	Open fractures involving skull or face with other bones with subarachnoid, subdural, and extradural hemorrhage, with brief [less than one hour] loss of consciousness
804.73	ICD9	Open fractures involving skull or face with other bones with subarachnoid, subdural, and extradural hemorrhage, with moderate [1-24 hours] loss of consciousness
804.74	ICD9	Open fractures involving skull or face with other bones with subarachnoid, subdural, and extradural hemorrhage, with prolonged [more than 24 hours] loss of consciousness and return to pre-existing conscious level

© 2023 Brenner LA et al. *JAMA Network Open.*

804.75	ICD9	Open fractures involving skull or face with other bones with subarachnoid, subdural, and extradural hemorrhage, with prolonged [more than 24 hours] loss of consciousness, without return to pre-existing conscious level
804.76	ICD9	Open fractures involving skull or face with other bones with subarachnoid, subdural, and extradural hemorrhage, with loss of consciousness of unspecified duration
804.79	ICD9	Open fractures involving skull or face with other bones with subarachnoid, subdural, and extradural hemorrhage, with concussion, unspecified
804.8	ICD9	Open fractures involving skull or face with other bones with other and unspecified intracranial hemorrhage
804.80	ICD9	Open fractures involving skull or face with other bones, with other and unspecified intracranial hemorrhage, unspecified state of consciousness
804.81	ICD9	Open fractures involving skull or face with other bones, with other and unspecified intracranial hemorrhage, with no loss of consciousness
804.82	ICD9	Open fractures involving skull or face with other bones, with other and unspecified intracranial hemorrhage, with brief [less than one hour] loss of consciousness
804.83	ICD9	Open fractures involving skull or face with other bones, with other and unspecified intracranial hemorrhage, with moderate [1-24 hours] loss of consciousness
804.84	ICD9	Open fractures involving skull or face with other bones, with other and unspecified intracranial hemorrhage, with prolonged [more than 24 hours] loss of consciousness and return to pre-existing conscious level
804.85	ICD9	Open fractures involving skull or face with other bones, with other and unspecified intracranial hemorrhage, with prolonged [more than 24 hours] loss consciousness, without return to pre-existing conscious level
804.86	ICD9	Open fractures involving skull or face with other bones, with other and unspecified intracranial hemorrhage, with loss of consciousness of unspecified duration
804.89	ICD9	Open fractures involving skull or face with other bones, with other and unspecified intracranial hemorrhage, with concussion, unspecified
804.9	ICD9	Open fractures involving skull or face with other bones with intracranial injury of other and unspecified nature
804.90	ICD9	Open fractures involving skull or face with other bones, with intracranial injury of other and unspecified nature, unspecified state of consciousness
804.91	ICD9	Open fractures involving skull or face with other bones, with intracranial injury of other and unspecified nature, with no loss of consciousness
804.92	ICD9	Open fractures involving skull or face with other bones, with intracranial injury of other and unspecified nature, with brief [less than one hour] loss of consciousness
804.93	ICD9	Open fractures involving skull or face with other bones, with intracranial injury of other and unspecified nature, with moderate [1-24 hours] loss of consciousness
804.94	ICD9	Open fractures involving skull or face with other bones, with intracranial injury of other and unspecified nature, with prolonged [more than 24 hours] loss of consciousness and return to pre-existing conscious level
804.95	ICD9	Open fractures involving skull or face with other bones, with intracranial injury of other and unspecified nature, with prolonged [more than 24 hours] loss of consciousness without return to pre-existing conscious level
804.96	ICD9	Open fractures involving skull or face with other bones, with intracranial injury of other and unspecified nature, with loss of consciousness of unspecified duration

© 2023 Brenner LA et al. *JAMA Network Open.*

804.99	ICD9	Open fractures involving skull or face with other bones, with intracranial injury of other and unspecified nature, with concussion, unspecified
850.	ICD9	Concussion
850.0	ICD9	Concussion with no loss of consciousness
850.1	ICD9	Concussion w/brief LOC
850.11	ICD9	Concussion, with loss of consciousness of 30 minutes or less
850.12	ICD9	Concussion, with loss of consciousness from 31 to 59 minutes
850.2	ICD9	Concussion with moderate loss of consciousness
850.3	ICD9	Concussion with prolonged loss of consciousness and return to pre-existing conscious level
850.4	ICD9	Concussion with prolonged loss of consciousness, without return to pre-existing conscious level
850.5	ICD9	Concussion with loss of consciousness of unspecified duration
850.9	ICD9	Concussion, unspecified
851.0	ICD9	Cortex (cerebral) contusion w/o mention of open intracranial wound
851.00	ICD9	Cortex (cerebral) contusion without mention of open intracranial wound, unspecified state of consciousness
851.01	ICD9	Cortex (cerebral) contusion without mention of open intracranial wound, with no loss of consciousness
851.02	ICD9	Cortex (cerebral) contusion without mention of open intracranial wound, with brief [less than one hour] loss of consciousness
851.03	ICD9	Cortex (cerebral) contusion without mention of open intracranial wound, with moderate [1-24 hours] loss of consciousness
851.04	ICD9	Cortex (cerebral) contusion without mention of open intracranial wound, with prolonged [more than 24 hours] loss of consciousness and return to pre-existing conscious level
851.05	ICD9	Cortex (cerebral) contusion without mention of open intracranial wound, with prolonged [more than 24 hours] loss of consciousness without return to pre-existing conscious level
851.06	ICD9	Cortex (cerebral) contusion without mention of open intracranial wound, with loss of consciousness of unspecified duration
851.09	ICD9	Cortex (cerebral) contusion without mention of open intracranial wound, with concussion, unspecified
851.1	ICD9	Cortex (cerebral) contusion with open intracranial wound
851.10	ICD9	Cortex (cerebral) contusion with open intracranial wound, unspecified state of consciousness
851.11	ICD9	Cortex (cerebral) contusion with open intracranial wound, with no loss of consciousness
851.12	ICD9	Cortex (cerebral) contusion with open intracranial wound, with brief [less than one hour] loss of consciousness
851.13	ICD9	Cortex (cerebral) contusion with open intracranial wound, with moderate [1-24 hours] loss of consciousness
851.14	ICD9	Cortex (cerebral) contusion with open intracranial wound, with prolonged [more than 24 hours] loss of consciousness and return to pre-existing conscious level
851.15	ICD9	Cortex (cerebral) contusion with open intracranial wound, with prolonged [more than 24 hours] loss of consciousness without return to pre-existing conscious level
851.16	ICD9	Cortex (cerebral) contusion with open intracranial wound, with loss of consciousness of unspecified duration
851.19	ICD9	Cortex (cerebral) contusion with open intracranial wound, with concussion, unspecified

© 2023 Brenner LA et al. *JAMA Network Open.*

851.2	ICD9	Cortex (cerebral) laceration w/o mention of open intracranial wound
851.20	ICD9	Cortex (cerebral) laceration without mention of open intracranial wound, unspecified state of consciousness
851.21	ICD9	Cortex (cerebral) laceration without mention of open intracranial wound, with no loss of consciousness
851.22	ICD9	Cortex (cerebral) laceration without mention of open intracranial wound, with brief [less than one hour] loss of consciousness
851.23	ICD9	Cortex (cerebral) laceration without mention of open intracranial wound, with moderate [1-24 hours] loss of consciousness
851.24	ICD9	Cortex (cerebral) laceration without mention of open intracranial wound, with prolonged [more than 24 hours] loss of consciousness and return to pre-existing conscious level
851.25	ICD9	Cortex (cerebral) laceration without mention of open intracranial wound, with prolonged [more than 24 hours] loss of consciousness without return to pre-existing conscious level
851.26	ICD9	Cortex (cerebral) laceration without mention of open intracranial wound, with loss of consciousness of unspecified duration
851.29	ICD9	Cortex (cerebral) laceration without mention of open intracranial wound, with concussion, unspecified
851.3	ICD9	Cortex (derebral) laceration with open intracranial wound
851.30	ICD9	Cortex (cerebral) laceration with open intracranial wound, unspecified state of consciousness
851.31	ICD9	Cortex (cerebral) laceration with open intracranial wound, with no loss of consciousness
851.32	ICD9	Cortex (cerebral) laceration with open intracranial wound, with brief [less than one hour] loss of consciousness
851.33	ICD9	Cortex (cerebral) laceration with open intracranial wound, with moderate [1-24 hours] loss of consciousness
851.34	ICD9	Cortex (cerebral) laceration with open intracranial wound, with prolonged [more than 24 hours] loss of consciousness and return to pre-existing conscious level
851.35	ICD9	Cortex (cerebral) laceration with open intracranial wound, with prolonged [more than 24 hours] loss of consciousness without return to pre-existing conscious level
851.36	ICD9	Cortex (cerebral) laceration with open intracranial wound, with loss of consciousness of unspecified duration
851.39	ICD9	Cortex (cerebral) laceration with open intracranial wound, with concussion, unspecified
851.4	ICD9	Cerebellar or brain stem contusion w/o mention of open intracranial wound
851.40	ICD9	Cerebellar or brain stem contusion without mention of open intracranial wound, unspecified state of consciousness
851.41	ICD9	Cerebellar or brain stem contusion without mention of open intracranial wound, with no loss of consciousness
851.42	ICD9	Cerebellar or brain stem contusion without mention of open intracranial wound, with brief [less than one hour] loss of consciousness
851.43	ICD9	Cerebellar or brain stem contusion without mention of open intracranial wound, with moderate [1-24 hours] loss of consciousness
851.44	ICD9	Cerebellar or brain stem contusion without mention of open intracranial wound, with prolonged [more than 24 hours] loss consciousness and return to pre-existing conscious level
851.45	ICD9	Cerebellar or brain stem contusion without mention of open intracranial wound, with prolonged [more than 24 hours] loss of consciousness without return to pre-existing conscious level

© 2023 Brenner LA et al. *JAMA Network Open.*

851.46	ICD9	Cerebellar or brain stem contusion without mention of open intracranial wound, with loss of consciousness of unspecified duration
851.49	ICD9	Cerebellar or brain stem contusion without mention of open intracranial wound, with concussion, unspecified
851.5	ICD9	Cerebellar or brain stem contusion with open intracranial wound
851.50	ICD9	Cerebellar or brain stem contusion with open intracranial wound, unspecified state of consciousness
851.51	ICD9	Cerebellar or brain stem contusion with open intracranial wound, with no loss of consciousness
851.52	ICD9	Cerebellar or brain stem contusion with open intracranial wound, with brief [less than one hour] loss of consciousness
851.53	ICD9	Cerebellar or brain stem contusion with open intracranial wound, with moderate [1-24 hours] loss of consciousness
851.54	ICD9	Cerebellar or brain stem contusion with open intracranial wound, with prolonged [more than 24 hours] loss of consciousness and return to pre-existing conscious level
851.55	ICD9	Cerebellar or brain stem contusion with open intracranial wound, with prolonged [more than 24 hours] loss of consciousness without return to pre-existing conscious level
851.56	ICD9	Cerebellar or brain stem contusion with open intracranial wound, with loss of consciousness of unspecified duration
851.59	ICD9	Cerebellar or brain stem contusion with open intracranial wound, with concussion, unspecified
851.6	ICD9	Cerebellar or brain stem laceration w/o mention of open intracranial wound
851.60	ICD9	Cerebellar or brain stem laceration without mention of open intracranial wound, unspecified state of consciousness
851.61	ICD9	Cerebellar or brain stem laceration without mention of open intracranial wound, with no loss of consciousness
851.62	ICD9	Cerebellar or brain stem laceration without mention of open intracranial wound, with brief [less than 1 hour] loss of consciousness
851.63	ICD9	Cerebellar or brain stem laceration without mention of open intracranial wound, with moderate [1-24 hours] loss of consciousness
851.64	ICD9	Cerebellar or brain stem laceration without mention of open intracranial wound, with prolonged [more than 24 hours] loss of consciousness and return to pre-existing conscious level
851.65	ICD9	Cerebellar or brain stem laceration without mention of open intracranial wound, with prolonged [more than 24 hours] loss of consciousness without return to pre-existing conscious level
851.66	ICD9	Cerebellar or brain stem laceration without mention of open intracranial wound, with loss of consciousness of unspecified duration
851.69	ICD9	Cerebellar or brain stem laceration without mention of open intracranial wound, with concussion, unspecified
851.7	ICD9	Cerebellar or brain stem laceration with open intracranial wound
851.70	ICD9	Cerebellar or brain stem laceration with open intracranial wound, unspecified state of consciousness
851.71	ICD9	Cerebellar or brain stem laceration with open intracranial wound, with no loss of consciousness
851.72	ICD9	Cerebellar or brain stem laceration with open intracranial wound, with brief [less than one hour] loss of consciousness

© 2023 Brenner LA et al. *JAMA Network Open.*

851.73	ICD9	Cerebellar or brain stem laceration with open intracranial wound, with moderate [1-24 hours] loss of consciousness
851.74	ICD9	Cerebellar or brain stem laceration with open intracranial wound, with prolonged [more than 24 hours] loss of consciousness and return to pre-existing conscious level
851.75	ICD9	Cerebellar or brain stem laceration with open intracranial wound, with prolonged [more than 24 hours] loss of consciousness without return to pre-existing conscious level
851.76	ICD9	Cerebellar or brain stem laceration with open intracranial wound, with loss of consciousness of unspecified duration
851.79	ICD9	Cerebellar or brain stem laceration with open intracranial wound, with concussion, unspecified
851.8	ICD9	Other and unspecified cerebral laceration and contusion w/o mention of open intracranial wound
851.80	ICD9	Other and unspecified cerebral laceration and contusion, without mention of open intracranial wound, unspecified state of consciousness
851.81	ICD9	Other and unspecified cerebral laceration and contusion, without mention of open intracranial wound, with no loss of consciousness
851.82	ICD9	Other and unspecified cerebral laceration and contusion, without mention of open intracranial wound, with brief [less than one hour] loss of consciousness
851.83	ICD9	Other and unspecified cerebral laceration and contusion, without mention of open intracranial wound, with moderate [1-24 hours] loss of consciousness
851.84	ICD9	Other and unspecified cerebral laceration and contusion, without mention of open intracranial wound, with prolonged [more than 24 hours] loss of consciousness and return to pre- existing conscious level
851.85	ICD9	Other and unspecified cerebral laceration and contusion, without mention of open intracranial wound, with prolonged [more than 24 hours] loss of consciousness without return to pre-existing conscious level
851.86	ICD9	Other and unspecified cerebral laceration and contusion, without mention of open intracranial wound, with loss of consciousness of unspecified duration
851.89	ICD9	Other and unspecified cerebral laceration and contusion, without mention of open intracranial wound, with concussion, unspecified
851.9	ICD9	Other and unspecified cerebral laceration and contusion with open intracranial wound
851.90	ICD9	Other and unspecified cerebral laceration and contusion, with open intracranial wound, unspecified state of consciousness
851.91	ICD9	Other and unspecified cerebral laceration and contusion, with open intracranial wound, with no loss of consciousness
851.92	ICD9	Other and unspecified cerebral laceration and contusion, with open intracranial wound, with brief [less than one hour] loss of consciousness
851.93	ICD9	Other and unspecified cerebral laceration and contusion, with open intracranial wound, with moderate [1-24 hours] loss of consciousness
851.94	ICD9	Other and unspecified cerebral laceration and contusion, with open intracranial wound, with prolonged [more than 24 hours] loss of consciousness and return to pre-existing conscious level
851.95	ICD9	Other and unspecified cerebral laceration and contusion, with open intracranial wound, with prolonged [more than 24 hours] loss of consciousness without return to pre-existing conscious level

© 2023 Brenner LA et al. *JAMA Network Open.*

851.96	ICD9	Other and unspecified cerebral laceration and contusion, with open intracranial wound, with loss of consciousness of unspecified duration
851.99	ICD9	Other and unspecified cerebral laceration and contusion, with open intracranial wound, with concussion, unspecified
852.0	ICD9	Subarachnoid hemorrhage following injury w/o mention of open intracranial wound
852.00	ICD9	Subarachnoid hemorrhage following injury without mention of open intracranial wound, unspecified state of consciousness
852.01	ICD9	Subarachnoid hemorrhage following injury without mention of open intracranial wound, with no loss of consciousness
852.02	ICD9	Subarachnoid hemorrhage following injury without mention of open intracranial wound, with brief [less than one hour] loss of consciousness
852.03	ICD9	Subarachnoid hemorrhage following injury without mention of open intracranial wound, with moderate [1-24 hours] loss of consciousness
852.04	ICD9	Subarachnoid hemorrhage following injury without mention of open intracranial wound, with prolonged [more than 24 hours] loss of consciousness and return to pre-existing conscious level
852.05	ICD9	Subarachnoid hemorrhage following injury without mention of open intracranial wound, with prolonged [more than 24 hours] loss of consciousness without return to pre-existing conscious level
852.06	ICD9	Subarachnoid hemorrhage following injury without mention of open intracranial wound, with loss of consciousness of unspecified duration
852.09	ICD9	Subarachnoid hemorrhage following injury without mention of open intracranial wound, with concussion, unspecified
852.1	ICD9	Subarachnoid hemorrhage following injury with open intracranial wound
852.10	ICD9	Subarachnoid hemorrhage following injury with open intracranial wound, unspecified state of consciousness
852.11	ICD9	Subarachnoid hemorrhage following injury with open intracranial wound, with no loss of consciousness
852.12	ICD9	Subarachnoid hemorrhage following injury with open intracranial wound, with brief [less than one hour] loss of consciousness
852.13	ICD9	Subarachnoid hemorrhage following injury with open intracranial wound, with moderate [1-24 hours] loss of consciousness
852.14	ICD9	Subarachnoid hemorrhage following injury with open intracranial wound, with prolonged [more than 24 hours) loss of consciousness and return to pre-existing conscious level
852.15	ICD9	Subarachnoid hemorrhage following injury with open intracranial wound, with prolonged [more than 24 hours] loss of consciousness without return to pre-existing conscious level
852.16	ICD9	Subarachnoid hemorrhage following injury with open intracranial wound, with loss of consciousness of unspecified duration
852.19	ICD9	Subarachnoid hemorrhage following injury with open intracranial wound, with concussion, unspecified
852.2	ICD9	Subdural hemorrhage following injury w/o mention of open intracranial wound
852.20	ICD9	Subdural hemorrhage following injury without mention of open intracranial wound, unspecified state of consciousness

© 2023 Brenner LA et al. *JAMA Network Open.*

852.21	ICD9	Subdural hemorrhage following injury without mention of open intracranial wound, with no loss of consciousness
852.22	ICD9	Subdural hemorrhage following injury without mention of open intracranial wound, with brief [less than one hour] loss of consciousness
852.23	ICD9	Subdural hemorrhage following injury without mention of open intracranial wound, with moderate [1-24 hours] loss of consciousness
852.24	ICD9	Subdural hemorrhage following injury without mention of open intracranial wound, with prolonged [more than 24 hours] loss of consciousness and return to pre-existing conscious level
852.25	ICD9	Subdural hemorrhage following injury without mention of open intracranial wound, with prolonged [more than 24 hours] loss of consciousness without return to pre-existing conscious level
852.26	ICD9	Subdural hemorrhage following injury without mention of open intracranial wound, with loss of consciousness of unspecified duration
852.29	ICD9	Subdural hemorrhage following injury without mention of open intracranial wound, with concussion, unspecified
852.3	ICD9	Subdural hemorrhage following injury with open intracranial wound
852.30	ICD9	Subdural hemorrhage following injury with open intracranial wound, unspecified state of consciousness
852.31	ICD9	Subdural hemorrhage following injury with open intracranial wound, with no loss of consciousness
852.32	ICD9	Subdural hemorrhage following injury with open intracranial wound, with brief [less than one hour] loss of consciousness
852.33	ICD9	Subdural hemorrhage following injury with open intracranial wound, with moderate [1-24 hours] loss of consciousness
852.34	ICD9	Subdural hemorrhage following injury with open intracranial wound, with prolonged [more than 24 hours] loss of consciousness and return to pre-existing conscious level
852.35	ICD9	Subdural hemorrhage following injury with open intracranial wound, with prolonged [more than 24 hours] loss of consciousness without return to pre-existing conscious level
852.36	ICD9	Subdural hemorrhage following injury with open intracranial wound, with loss of consciousness of unspecified duration
852.39	ICD9	Subdural hemorrhage following injury with open intracranial wound, with concussion, unspecified
852.4	ICD9	Extradural hemorrhage following injury w/o mention of open intracranial wound
852.40	ICD9	Extradural hemorrhage following injury without mention of open intracranial wound, unspecified state of consciousness
852.41	ICD9	Extradural hemorrhage following injury without mention of open intracranial wound, with no loss of consciousness
852.42	ICD9	Extradural hemorrhage following injury without mention of open intracranial wound, with brief [less than 1 hour] loss of consciousness
852.43	ICD9	Extradural hemorrhage following injury without mention of open intracranial wound, with moderate [1-24 hours] loss of consciousness
852.44	ICD9	Extradural hemorrhage following injury without mention of open intracranial wound, with prolonged [more than 24 hours] loss of consciousness and return to pre-existing conscious level

© 2023 Brenner LA et al. *JAMA Network Open.*

852.45	ICD9	Extradural hemorrhage following injury without mention of open intracranial wound, with prolonged [more than 24 hours] loss of consciousness without return to pre-existing conscious level
852.46	ICD9	Extradural hemorrhage following injury without mention of open intracranial wound, with loss of consciousness of unspecified duration
852.49	ICD9	Extradural hemorrhage following injury without mention of open intracranial wound, with concussion, unspecified
852.5	ICD9	Extradural hemorrhage following injury with open intracranial wound
852.50	ICD9	Extradural hemorrhage following injury with open intracranial wound, unspecified state of consciousness
852.51	ICD9	Extradural hemorrhage following injury with open intracranial wound, with no loss of consciousness
852.52	ICD9	Extradural hemorrhage following injury with open intracranial wound, with brief [less than one hour] loss of consciousness
852.53	ICD9	Extradural hemorrhage following injury with open intracranial wound, with moderate [1-24 hours] loss of consciousness
852.54	ICD9	Extradural hemorrhage following injury with open intracranial wound, with prolonged [more than 24 hours] loss of consciousness and return to pre-existing conscious level
852.55	ICD9	Extradural hemorrhage following injury with open intracranial wound, with prolonged [more than 24 hours] loss of consciousness without return to pre-existing conscious level
852.56	ICD9	Extradural hemorrhage following injury with open intracranial wound, with loss of consciousness of unspecified duration
852.59	ICD9	Extradural hemorrhage following injury with open intracranial wound, with concussion, unspecified
853.0	ICD9	Other and unspecified intracranial hemorrhage following injury w/o mention of open intracranial wound
853.00	ICD9	Other and unspecified intracranial hemorrhage following injury without mention of open intracranial wound, unspecified state of consciousness
853.01	ICD9	Other and unspecified intracranial hemorrhage following injury without mention of open intracranial wound, with no loss of consciousness
853.02	ICD9	Other and unspecified intracranial hemorrhage following injury without mention of open intracranial wound, with brief [less than one hour] loss of consciousness
853.03	ICD9	Other and unspecified intracranial hemorrhage following injury without mention of open intracranial wound, with moderate [1-24 hours] loss of consciousness
853.04	ICD9	Other and unspecified intracranial hemorrhage following injury without mention of open intracranial wound, with prolonged [more than 24 hours] loss of consciousness and return to pre-existing conscious level
853.05	ICD9	Other and unspecified intracranial hemorrhage following injury without mention of open intracranial wound, with prolonged [more than 24 hours] loss of consciousness without return to pre-existing conscious level
853.06	ICD9	Other and unspecified intracranial hemorrhage following injury without mention of open intracranial wound, with loss of consciousness of unspecified duration
853.09	ICD9	Other and unspecified intracranial hemorrhage following injury without mention of open intracranial wound, with concussion, unspecified
853.1	ICD9	Other and unspecified intracranial hemorrhage following injury with open intracranial wound

© 2023 Brenner LA et al. *JAMA Network Open.*

853.10	ICD9	Other and unspecified intracranial hemorrhage following injury with open intracranial wound, unspecified state of consciousness
853.11	ICD9	Other and unspecified intracranial hemorrhage following injury with open intracranial wound, with no loss of consciousness
853.12	ICD9	Other and unspecified intracranial hemorrhage following injury with open intracranial wound, with brief [less than one hour] loss of consciousness
853.13	ICD9	Other and unspecified intracranial hemorrhage following injury with open intracranial wound, with moderate [1-24 hours] loss of consciousness
853.14	ICD9	Other and unspecified intracranial hemorrhage following injury with open intracranial wound, with prolonged [more than 24 hours] loss of consciousness and return to pre-existing conscious level
853.15	ICD9	Other and unspecified intracranial hemorrhage following injury with open intracranial wound, with prolonged [more than 24 hours] loss of consciousness without return to pre-existing conscious level
853.16	ICD9	Other and unspecified intracranial hemorrhage following injury with open intracranial wound, with loss of consciousness of unspecified duration
853.19	ICD9	Other and unspecified intracranial hemorrhage following injury with open intracranial wound, with concussion, unspecified
854.0	ICD9	Intracranial injury of other and unspecified nature w/o mention of open intracranial wound
854.00	ICD9	Intracranial injury of other and unspecified nature without mention of open intracranial wound, unspecified state of consciousness
854.01	ICD9	Intracranial injury of other and unspecified nature without mention of open intracranial wound, with no loss of consciousness
854.02	ICD9	Intracranial injury of other and unspecified nature without mention of open intracranial wound, with brief [less than one hour] loss of consciousness
854.03	ICD9	Intracranial injury of other and unspecified nature without mention of open intracranial wound, with moderate [1-24 hours] loss of consciousness
854.04	ICD9	Intracranial injury of other and unspecified nature without mention of open intracranial wound, with prolonged [more than 24 hours] loss of consciousness and return to pre-existing conscious level
854.05	ICD9	Intracranial injury of other and unspecified nature without mention of open intracranial wound, with prolonged [more than 24 hours] loss of consciousness without return to pre-existing conscious level
854.06	ICD9	Intracranial injury of other and unspecified nature without mention of open intracranial wound, with loss of consciousness of unspecified duration
854.09	ICD9	Intracranial injury of other and unspecified nature without mention of open intracranial wound, with concussion, unspecified
854.1	ICD9	Intracranial injury of other and unspecified nature with open Intracranial wound
854.10	ICD9	Intracranial injury of other and unspecified nature with open intracranial wound, unspecified state of consciousness
854.11	ICD9	Intracranial injury of other and unspecified nature with open intracranial wound, with no loss of consciousness
854.12	ICD9	Intracranial injury of other and unspecified nature with open intracranial wound, with brief [less than one hour] loss of consciousness

© 2023 Brenner LA et al. *JAMA Network Open.*

854.13	ICD9	Intracranial injury of other and unspecified nature with open intracranial wound, with moderate [1-24 hours] loss of consciousness
854.14	ICD9	Intracranial injury of other and unspecified nature with open intracranial wound, with prolonged [more than 24 hours] loss of consciousness and return to pre-existing conscious level
854.15	ICD9	Intracranial injury of other and unspecified nature with open intracranial wound, with prolonged [more than 24 hours] loss of consciousness without return to pre-existing conscious level
854.16	ICD9	Intracranial injury of other and unspecified nature with open intracranial wound, with loss of consciousness of unspecified duration
854.19	ICD9	Intracranial injury of other and unspecified nature with open intracranial wound, with concussion, unspecified
907.0	ICD9	Late effect of intracranial injury without mention of skull fracture
310.2 U	ICD9	Postconcussion syndrome
800.41 U	ICD9	Closed fracture of vault of skull with intracranial injury of other and unspecified nature, with no loss of consciousness
800.66 U	ICD9	Open fracture of vault of skull with cerebral laceration and contusion, with loss of consciousness of unspecified duration
801.06 U	ICD9	Closed fracture of base of skull without mention of intra cranial injury, with loss of consciousness of unspecified duration
801.11 U	ICD9	Closed fracture of base of skull with cerebral laceration and contusion, with no loss of consciousness
804.02 U	ICD9	Closed fractures involving skull or face with other bones, without mention of intracranial injury, with brief [less than one hour] loss of consciousness
850.11 U	ICD9	Concussion, with loss of consciousness of 30 minutes or less
850.12 U	ICD9	Concussion, with loss of consciousness from 31 to 59 minutes
850.5 U	ICD9	Concussion with loss of consciousness of unspecified duration
850.9 U	ICD9	Concussion, unspecified
850.9 Z	ICD9	Concussion, unspecified
851.00 U	ICD9	Cortex (cerebral) contusion without mention of open intracranial wound, unspecified state of consciousness
851.41 U	ICD9	Cerebellar or brain stem contusion without mention of open intracranial wound, with no loss of consciousness
851.81 Z	ICD9	Other and unspecified cerebral laceration and contusion, without mention of open intracranial wound, with no loss of consciousness
852.02 U	ICD9	Subarachnoid hemorrhage following injury without mention of open intracranial wound, with brief [less than one hour] loss of consciousness
852.02 Z	ICD9	Subarachnoid hemorrhage following injury without mention of open intracranial wound, with brief [less than one hour] loss of consciousness
852.20 U	ICD9	Subdural hemorrhage following injury without mention of open intracranial wound, unspecified state of consciousness
852.21 U	ICD9	Subdural hemorrhage following injury without mention of open intracranial wound, with no loss of consciousness

© 2023 Brenner LA et al. *JAMA Network Open.*

852.22 U	ICD9	Subdural hemorrhage following injury without mention of open intracranial wound, with brief [less than one hour] loss of consciousness
852.26 U	ICD9	Subdural hemorrhage following injury without mention of open intracranial wound, with loss of consciousness of unspecified duration
852.40 U	ICD9	Extradural hemorrhage following injury without mention of open intracranial wound, unspecified state of consciousness
854.02 U	ICD9	Intracranial injury of other and unspecified nature without mention of open intracranial wound, with brief [less than one hour] loss of consciousness
854.06 U	ICD9	Intracranial injury of other and unspecified nature without mention of open intracranial wound, with loss of consciousness of unspecified duration
V15.5 1	ICD9	Personal History Of Traumatic Brain Injury (TBI),Global War On Terrorism (GWOT) Related,Unknown Level Of Severity
V15.5 1U	ICD9	Personal History Of Traumatic Brain Injury (TBI),Global War On Terrorism (GWOT) Related,Unknown Level Of Severity
V15.5 2	ICD9	Personal History Of Traumatic Brain Injury (TBI),Global War On Terrorism (GWOT) Related,Highest Level Of Severity Mild (Glasgow Coma Scale 13-15),Loc<1Hr,Post Trauma Amnesia<24Hr
V15.5 2U	ICD9	Personal History Of Traumatic Brain Injury (TBI),Global War On Terrorism (GWOT) Related,Highest Level Of Severity Mild (Glasgow Coma Scale 13-15),Loc<1Hr,Post Trauma Amnesia<24Hr
V15.5 3	ICD9	Personal History Of Traumatic Brain Injury (TBI),Global War On Terrorism (GWOT) Related,Highest Level Of Severity Moderate (Glasgow Coma Scale 9-12),Loc 1-24 Hrs,Post Trauma Amnesia 2-7 Days
V15.5 4	ICD9	Personal History Of Traumatic Brain Injury (TBI),Global War On Terrorism (GWOT) Related,Highest Level Of Severity Severe (Glasgow Coma Scale 3-8),Loc >24Hrs,Post Trauma Amnesia >7 Days
V15.5 5	ICD9	Personal History Of Traumatic Brain Injury (TBI),Global War On Terrorism (GWOT) Related,Penetrating Intracranial Wound (No Level Of Severity Assigned)
V15.5 6	ICD9	Personal History Of Traumatic Brain Injury (TBI), Not GWOT Related, Unknown Level Of Severity
V15.5 6U	ICD9	Personal History Of Traumatic Brain Injury (TBI), Not GWOT Related, Unknown Level Of Severity
V15.5 7	ICD9	Personal History Of Traumatic Brain Injury (TBI),Not Related To Global War On Terrorism (GWOT),Highest Level Of Severity Mild (Glasgow Coma Scale 13-15),Loc<1Hr,Post Trauma Amnesia<24Hr
V15.5 8	ICD9	Personal History Of Traumatic Brain Injury (TBI),Not Related To Global War On Terrorism (GWOT),Highest Level Of Severity Moderate (Glasgow Coma Scale 9-12),Loc 1-24 Hrs,Post Trauma Amnesia 2-7 Days
V15.5 9	ICD9	Personal History Of Traumatic Brain Injury (TBI),Not Related To Global War On Terrorism (GWOT),Highest Level Of Severity Severe (Glasgow Coma Scale 3-8),Loc >24Hrs,Post Trauma Amnesia >7 Days
V15.5 A	ICD9	Personal History Of Traumatic Brain Injury (TBI),Not Related To Global War On Terrorism (GWOT),Penetrating Intracranial Wound (No Level Of Severity Assigned)
V15.5 B	ICD9	Personal History Of Traumatic Brain Injury (TBI), Unknown If GWOT Related, Unknown Severity Level
V15.5 C	ICD9	Personal History Of Traumatic Brain Injury (TBI),Unknown If Related To Global War On Terrorism (GWOT),Highest Level Of Severity Mild (Glasgow Coma Scale 13-15),Loc<1Hr,Post Trauma Amnesia<24Hr

© 2023 Brenner LA et al. *JAMA Network Open.*

V15.5 E	ICD9	Personal History Of Traumatic Brain Injury (TBI),Unknown If Related To Global War On Terrorism (GWOT),Highest Level Of Severity Severe (Glasgow Coma Scale 3-8),Loc >24Hrs,Post Trauma Amnesia >7 Days
V15.5 1	ICD9	Personal History Of Traumatic Brain Injury (TBI),Global War On Terrorism (GWOT) Related,Unknown Level Of Severity
V15.5 2	ICD9	Personal History Of Traumatic Brain Injury (TBI),Global War On Terrorism (GWOT) Related,Highest Level Of Severity Mild (Glasgow Coma Scale 13-15),Loc<1Hr,Post Trauma Amnesia<24Hr
V15.5 3	ICD9	Personal History Of Traumatic Brain Injury (TBI),Global War On Terrorism (GWOT) Related,Highest Level Of Severity Moderate (Glasgow Coma Scale 9-12),Loc 1-24 Hrs,Post Trauma Amnesia 2-7 Days
V15.5 4	ICD9	Personal History Of Traumatic Brain Injury (TBI),Global War On Terrorism (GWOT) Related,Highest Level Of Severity Severe (Glasgow Coma Scale 3-8),Loc >24Hrs,Post Trauma Amnesia >7 Days
V15.5 5	ICD9	Personal History Of Traumatic Brain Injury (TBI),Global War On Terrorism (GWOT) Related,Penetrating Intracranial Wound (No Level Of Severity Assigned)
V15.5 6	ICD9	Personal History Of Traumatic Brain Injury (TBI), Not GWOT Related, Unknown Level Of Severity
V15.5 7	ICD9	Personal History Of Traumatic Brain Injury (TBI),Not Related To Global War On Terrorism (GWOT),Highest Level Of Severity Mild (Glasgow Coma Scale 13-15),Loc<1Hr,Post Trauma Amnesia<24Hr
V15.5 8	ICD9	Personal History Of Traumatic Brain Injury (TBI),Not Related To Global War On Terrorism (GWOT),Highest Level Of Severity Moderate (Glasgow Coma Scale 9-12),Loc 1-24 Hrs,Post Trauma Amnesia 2-7 Days
V15.5 9	ICD9	Personal History Of Traumatic Brain Injury (TBI),Not Related To Global War On Terrorism (GWOT),Highest Level Of Severity Severe (Glasgow Coma Scale 3-8),Loc >24Hrs,Post Trauma Amnesia >7 Days
V15.5 A	ICD9	Personal History Of Traumatic Brain Injury (TBI),Not Related To Global War On Terrorism (GWOT),Penetrating Intracranial Wound (No Level Of Severity Assigned)
V15.5 B	ICD9	Personal History Of Traumatic Brain Injury (TBI), Unknown If GWOT Related, Unknown Severity Level
V15.5 C	ICD9	Personal History Of Traumatic Brain Injury (TBI),Unknown If Related To Global War On Terrorism (GWOT),Highest Level Of Severity Mild (Glasgow Coma Scale 13-15),Loc<1Hr,Post Trauma Amnesia<24Hr
V15.5 D	ICD9	Personal History Of Traumatic Brain Injury (TBI),Unknown If Related To Global War On Terrorism (GWOT),Highest Level Of Severity Moderate (Glasgow Coma Scale 9-12),Loc 1-24 Hrs,Post Trauma Amnesia 2-7 Days
V15.5 E	ICD9	Personal History Of Traumatic Brain Injury (TBI),Unknown If Related To Global War On Terrorism (GWOT),Highest Level Of Severity Severe (Glasgow Coma Scale 3-8),Loc >24Hrs,Post Trauma Amnesia >7 Days
V15.5 F	ICD9	Personal History Of Traumatic Brain Injury (TBI), Unknown If Related To Global War On Terrorism (GWOT),Penetrating Intracranial Wound (No Level Of Severity Assigned)
V15.52	ICD9	Personal History Of Traumatic Brain Injury
V15.52 0	ICD9	Personal History Of Traumatic Brain Injury Not Otherwise Specified
V15.52 0U	ICD9	Personal History Of Traumatic Brain Injury Not Otherwise Specified
V15.52 1	ICD9	Personal History Of Traumatic Brain Injury (TBI),Global War On Terrorism (GWOT) Related,Unknown Level Of Severity

© 2023 Brenner LA et al. *JAMA Network Open.*

V15.52 1U	ICD9	Personal History Of Traumatic Brain Injury (TBI),Global War On Terrorism (GWOT) Related,Unknown Level Of Severity
V15.52 2	ICD9	Personal History Of Traumatic Brain Injury (TBI),Global War On Terrorism (GWOT) Related,Highest Level Of Severity Mild
V15.52 2U	ICD9	Personal History Of Traumatic Brain Injury (TBI),Global War On Terrorism (GWOT) Related,Highest Level Of Severity Mild
V15.52 3	ICD9	Personal History Of Traumatic Brain Injury (TBI),Global War On Terrorism (GWOT) Related,Highest Level Of Severity Moderate
V15.52 3U	ICD9	Personal History Of Traumatic Brain Injury (TBI),Global War On Terrorism (GWOT) Related,Highest Level Of Severity Moderate
V15.52 4	ICD9	Personal History Of Traumatic Brain Injury (TBI),Global War On Terrorism (GWOT) Related,Highest Level Of Severity Severe
V15.52 5	ICD9	Personal History Of Traumatic Brain Injury (TBI),Global War On Terrorism (GWOT) Related,Penetrating Intracranial Wound (No Level Of Severity Assigned)
V15.52 6	ICD9	Personal History Of Traumatic Brain Injury (TBI), Not GWOT Related, Unknown Level Of Severity
V15.52 6U	ICD9	Personal History Of Traumatic Brain Injury (TBI), Not GWOT Related, Unknown Level Of Severity
V15.52 7	ICD9	Personal History Of Traumatic Brain Injury (TBI),Not Related To Global War On Terrorism (GWOT),Highest Level Of Severity Mild
V15.52 8	ICD9	Personal History Of Traumatic Brain Injury (TBI),Not Related To Global War On Terrorism (GWOT),Highest Level Of Severity Moderate
V15.52 9	ICD9	Personal History Of Traumatic Brain Injury (TBI),Not Related To Global War On Terrorism (GWOT),Highest Level Of Severity Severe
V15.52 9U	ICD9	Personal History Of Traumatic Brain Injury (TBI),Not Related To Global War On Terrorism (GWOT),Highest Level Of Severity Severe
V15.52 A	ICD9	Personal History Of Traumatic Brain Injury (TBI),Not Related To Global War On Terrorism (GWOT),Penetrating Intracranial Wound (No Level Of Severity Assigned)
V15.52 B	ICD9	Personal History Of Traumatic Brain Injury (TBI), Unknown If GWOT Related, Unknown Severity Level
V15.52 C	ICD9	Personal History Of Traumatic Brain Injury (TBI),Unknown If Related To Global War On Terrorism (GWOT),Highest Level Of Severity Mild
V15.52 CU	ICD9	Personal History Of Traumatic Brain Injury (TBI),Unknown If Related To Global War On Terrorism (GWOT),Highest Level Of Severity Mild
V15.52 D	ICD9	Personal History Of Traumatic Brain Injury (TBI),Unknown If Related To Global War On Terrorism (GWOT),Highest Level Of Severity Moderate
V15.52 E	ICD9	Personal History Of Traumatic Brain Injury (TBI),Unknown If Related To Global War On Terrorism (GWOT),Highest Level Of Severity Severe
V15.52 F	ICD9	Personal History Of Traumatic Brain Injury (TBI), Unknown If Related To Global War On Terrorism (GWOT),Penetrating Intracranial Wound
V15.52 FU	ICD9	Personal History Of Traumatic Brain Injury (TBI), Unknown If Related To Global War On Terrorism (GWOT),Penetrating Intracranial Wound

© 2023 Brenner LA et al. *JAMA Network Open.*

V15.59 1	ICD9	Personal History Of Traumatic Brain Injury (TBI),Global War On Terrorism (GWOT) Related,Unknown Level Of Severity
V15.59 1U	ICD9	Personal History Of Traumatic Brain Injury (TBI),Global War On Terrorism (GWOT) Related,Unknown Level Of Severity
V15.59 2	ICD9	Personal History Of Traumatic Brain Injury (TBI),Global War On Terrorism (GWOT) Related,Highest Level Of Severity Mild (Glasgow Coma Scale 13-15),Loc<1Hr,Post Trauma Amnesia<24Hr
V15.59 2U	ICD9	Personal History Of Traumatic Brain Injury (TBI),Global War On Terrorism (GWOT) Related,Highest Level Of Severity Mild (Glasgow Coma Scale 13-15),Loc<1Hr,Post Trauma Amnesia<24Hr
V15.59 3	ICD9	Personal History Of Traumatic Brain Injury (TBI),Global War On Terrorism (GWOT) Related,Highest Level Of Severity Moderate (Glasgow Coma Scale 9-12),Loc 1-24 Hrs,Post Trauma Amnesia 2-7 Days
V15.59 4	ICD9	Personal History Of Traumatic Brain Injury (TBI),Global War On Terrorism (GWOT) Related,Highest Level Of Severity Severe (Glasgow Coma Scale 3-8),Loc >24Hrs,Post Trauma Amnesia >7 Days
V15.59 5	ICD9	Personal History Of Traumatic Brain Injury (TBI),Global War On Terrorism (GWOT) Related,Penetrating Intracranial Wound (No Level Of Severity Assigned)
V15.59 6	ICD9	Personal History Of Traumatic Brain Injury (TBI), Not GWOT Related, Unknown Level Of Severity
V15.59 6U	ICD9	Personal History Of Traumatic Brain Injury (TBI), Not GWOT Related, Unknown Level Of Severity
V15.59 7	ICD9	Personal History Of Traumatic Brain Injury (TBI),Not Related To Global War On Terrorism (GWOT),Highest Level Of Severity Mild (Glasgow Coma Scale 13-15),Loc<1Hr,Post Trauma Amnesia<24Hr
V15.59 8	ICD9	Personal History Of Traumatic Brain Injury (TBI),Not Related To Global War On Terrorism (GWOT),Highest Level Of Severity Moderate (Glasgow Coma Scale 9-12),Loc 1-24 Hrs,Post Trauma Amnesia 2-7 Days
V15.59 9	ICD9	Personal History Of Traumatic Brain Injury (TBI),Not Related To Global War On Terrorism (GWOT),Highest Level Of Severity Severe (Glasgow Coma Scale 3-8),Loc >24Hrs,Post Trauma Amnesia >7 Days
V15.59 A	ICD9	Personal History Of Traumatic Brain Injury (TBI),Not Related To Global War On Terrorism (GWOT),Penetrating Intracranial Wound (No Level Of Severity Assigned)
V15.59 B	ICD9	Personal History Of Traumatic Brain Injury (TBI), Unknown If GWOT Related, Unknown Severity Level
V15.59 C	ICD9	Personal History Of Traumatic Brain Injury (TBI),Unknown If Related To Global War On Terrorism (GWOT),Highest Level Of Severity Mild (Glasgow Coma Scale 13-15),Loc<1Hr,Post Trauma Amnesia<24Hr
V15.59 D	ICD9	Personal History Of Traumatic Brain Injury (TBI),Unknown If Related To Global War On Terrorism (GWOT),Highest Level Of Severity Moderate (Glasgow Coma Scale 9-12),Loc 1-24 Hrs,Post Trauma Amnesia 2-7 Days
V15.59 E	ICD9	Personal History Of Traumatic Brain Injury (TBI),Unknown If Related To Global War On Terrorism (GWOT),Highest Level Of Severity Severe (Glasgow Coma Scale 3-8),Loc >24Hrs,Post Trauma Amnesia >7 Days
V15.59 F	ICD9	Personal History Of Traumatic Brain Injury (TBI), Unknown If Related To Global War On Terrorism (GWOT),Penetrating Intracranial Wound (No Level Of Severity Assigned)
DOD.0101	ICD10	Personal History Of Traumatic Brain Injury (TBI) - Unknown
DOD.0102	ICD10	Personal History Of Traumatic Brain Injury (TBI) - Mild
DOD.0103	ICD10	Personal History Of Traumatic Brain Injury (TBI) - Moderate

© 2023 Brenner LA et al. *JAMA Network Open.*

DOD.0104	ICD10	Personal History Of Traumatic Brain Injury (TBI) - Severe
DOD.0105	ICD10	Personal History Of Traumatic Brain Injury (TBI) - Penetrating
F07.81	ICD10	Postconcussional syndrome
S04.04	ICD10	Injury of visual cortex
S04.041	ICD10	Injury of visual cortex, right eye
S04.041A	ICD10	Injury of visual cortex, right side, initial encounter
S04.041D	ICD10	Injury of visual cortex, right side, subsequent encounter
S04.041S	ICD10	Injury of visual cortex, right side, sequela
S04.042	ICD10	Injury of visual cortex, left eye
S04.042A	ICD10	Injury of visual cortex, left side, initial encounter
S04.042D	ICD10	Injury of visual cortex, left side, subsequent encounter
S04.042S	ICD10	Injury of visual cortex, left side, sequela
S04.049	ICD10	Injury of visual cortex, unspecified eye
S04.049A	ICD10	Injury of visual cortex, unspecified side, initial encounter
S04.049D	ICD10	Injury of visual cortex, unspecified side, subsequent encounter
S04.049S	ICD10	Injury of visual cortex, unspecified side, sequela
S06.0	ICD10	Concussion
S06.0X0	ICD10	Concussion w/o LOC
S06.0X0A	ICD10	Concussion without loss of consciousness, initial encounter
S06.0X0D	ICD10	Concussion without loss of consciousness, subsequent encounter
S06.0X0S	ICD10	Concussion without loss of consciousness, sequela
S06.0X1	ICD10	Concussion w/LOC of 30 mins or less
S06.0X1A	ICD10	Concussion with loss of consciousness of 30 minutes or less, initial encounter
S06.0X1D	ICD10	Concussion with loss of consciousness of 30 minutes or less, subsequent encounter
S06.0X1S	ICD10	Concussion with loss of consciousness of 30 minutes or less, sequela
S06.0X2	ICD10	Concussion with loss of consciousness of 31 minutes to 59 minutes
S06.0X2A	ICD10	Concussion with loss of consciousness of 31 minutes to 59 minutes, initial encounter
S06.0X2D	ICD10	Concussion with loss of consciousness of 31 minutes to 59 minutes, subsequent encounter
S06.0X2S	ICD10	Concussion with loss of consciousness of 31 minutes to 59 minutes, sequela
S06.0X3	ICD10	Concussion with loss of consciousness of 1 hour to 5 hours 59 minutes
S06.0X3A	ICD10	Concussion with loss of consciousness of 1 hour to 5 hours 59 minutes, initial encounter
S06.0X3D	ICD10	Concussion with loss of consciousness of 1 hour to 5 hours 59 minutes, subsequent encounter
S06.0X3S	ICD10	Concussion with loss of consciousness of 1 hour to 5 hours 59 minutes, sequela
S06.0X4	ICD10	Concussion with loss of consciousness of 6 hours to 24 hours
S06.0X4A	ICD10	Concussion with loss of consciousness of 6 hours to 24 hours, initial encounter

© 2023 Brenner LA et al. *JAMA Network Open.*

S06.0X4D	ICD10	Concussion with loss of consciousness of 6 hours to 24 hours, subsequent encounter
S06.0X4S	ICD10	Concussion without loss of consciousness, sequela
S06.0X5	ICD10	Concussion with loss of consciousness greater than 24 hours with return to pre-existing conscious level
S06.0X5A	ICD10	Concussion with loss of consciousness greater than 24 hours with return to pre-existing conscious level, initial encounter
S06.0X5D	ICD10	Concussion with loss of consciousness greater than 24 hours with return to pre-existing conscious level, subsequent encounter
S06.0X5S	ICD10	Concussion with loss of consciousness greater than 24 hours with return to pre-existing conscious level, sequela
S06.0X6	ICD10	Concussion with loss of consciousness greater than 24 hours without return to pre-existing conscious level with patient surviving
S06.0X6A	ICD10	Concussion with loss of consciousness greater than 24 hours without return to pre-existing conscious level with patient surviving, initial encounter
S06.0X6D	ICD10	Concussion with loss of consciousness greater than 24 hours without return to pre-existing conscious level with patient surviving, initial encounter
S06.0X6S	ICD10	Concussion with loss of consciousness greater than 24 hours without return to pre-existing conscious level with patient surviving, sequela
S06.0X7	ICD10	Concussion with loss of consciousness of any duration with death due to brain injury prior to regaining consciousness
S06.0X7A	ICD10	Concussion with loss of consciousness of any duration with death due to brain injury prior to regaining consciousness, initial encounter
S06.0X7D	ICD10	Concussion with loss of consciousness of any duration with death due to brain injury prior to regaining consciousness, subsequent encounter
S06.0X7S	ICD10	Concussion with loss of consciousness of any duration with death due to brain injury prior to regaining consciousness, sequela
S06.0X8	ICD10	Concussion with loss of consciousness of any duration with death due to other cause prior to regaining consciousness
S06.0X8A	ICD10	Concussion with loss of consciousness of any duration with death due to other cause prior to regaining consciousness, initial encounter
S06.0X8D	ICD10	Concussion with loss of consciousness of any duration with death due to other cause prior to regaining consciousness, subsequent encounter
S06.0X8S	ICD10	Concussion with loss of consciousness of any duration with death due to other cause prior to regaining consciousness, sequela
S06.0X9	ICD10	Concussion w/LOC of unspecified duration
S06.0X9A	ICD10	Concussion with loss of consciousness of unspecified duration, initial encounter
S06.0X9D	ICD10	Concussion with loss of consciousness of unspecified duration, subsequent encounter
S06.0X9S	ICD10	Concussion with loss of consciousness of unspecified duration, sequela
S06.1X	ICD10	Traumatic cerebral edema

© 2023 Brenner LA et al. *JAMA Network Open.*

S06.1X0	ICD10	Traumatic cerebral edema w/o LOC
S06.1X0A	ICD10	Traumatic cerebral edema without loss of consciousness, initial encounter
S06.1X0D	ICD10	Traumatic cerebral edema without loss of consciousness, subsequent encounter
S06.1X0S	ICD10	Traumatic cerebral edema without loss of consciousness, sequela
S06.1X1	ICD10	Traumatic cerebral edema w/LOC of 30 min or less
S06.1X1A	ICD10	Traumatic cerebral edema with loss of consciousness of 30 minutes or less, initial encounter
S06.1X1D	ICD10	Traumatic cerebral edema with loss of consciousness of 30 minutes or less, subsequent encounter
S06.1X1S	ICD10	Traumatic cerebral edema with loss of consciousness of 30 minutes or less, sequela
S06.1X2	ICD10	Traumatic cerebral edema w/LOC of 31-59 minutes
S06.1X2A	ICD10	Traumatic cerebral edema with loss of consciousness of 31 minutes to 59 minutes, initial encounter
S06.1X2D	ICD10	Traumatic cerebral edema with loss of consciousness of 31 minutes to 59 minutes, subsequent encounter
S06.1X2S	ICD10	Traumatic cerebral edema with loss of consciousness of 31 minutes to 59 minutes, sequela
S06.1X3	ICD10	Traumatic cerebral edema w/LOC of 1hr to 5 hrs 59 mins
S06.1X3A	ICD10	Traumatic cerebral edema with loss of consciousness of 1 hour to 5 hours 59 minutes, initial encounter
S06.1X3D	ICD10	Traumatic cerebral edema with loss of consciousness of 1 hour to 5 hours 59 minutes, subsequent encounter
S06.1X3S	ICD10	Traumatic cerebral edema with loss of consciousness of 1 hour to 5 hours 59 minutes, sequela
S06.1X4	ICD10	Traumatic cerebral edema w/LOC of 6-24 hrs
S06.1X4A	ICD10	Traumatic cerebral edema with loss of consciousness of 6 hours to 24 hours, initial encounter
S06.1X4D	ICD10	Traumatic cerebral edema with loss of consciousness of 6 hours to 24 hours, subsequent encounter
S06.1X4S	ICD10	Traumatic cerebral edema with loss of consciousness of 6 hours to 24 hours, sequela
S06.1X5	ICD10	Traumatic cerebral edema w/ LOC >24 hrs with return to pre-existing conscious level
S06.1X5A	ICD10	Traumatic cerebral edema with loss of consciousness greater than 24 hours with return to pre-existing conscious level, initial encounter
S06.1X5D	ICD10	Traumatic cerebral edema with loss of consciousness greater than 24 hours with return to pre-existing conscious level, subsequent encounter
S06.1X5S	ICD10	Traumatic cerebral edema with loss of consciousness greater than 24 hours with return to pre-existing conscious level, sequela
S06.1X6	ICD10	Traumatic cerebral edema w/ LOC >24 hrs w/o return to pre-existing conscious level with patient surviving
S06.1X6A	ICD10	Traumatic cerebral edema with loss of consciousness greater than 24 hours without return to pre-existing conscious level with patient surviving, initial encounter
S06.1X6D	ICD10	Traumatic cerebral edema with loss of consciousness greater than 24 hours without return to pre-existing conscious level with patient surviving, subsequent encounter
S06.1X6S	ICD10	Traumatic cerebral edema with loss of consciousness greater than 24 hours without return to pre-existing conscious level with patient surviving, sequela
S06.1X7	ICD10	Traumatic cerebral edema w/LOC of any duration with death due to brain injury prior to regaining consciousness

© 2023 Brenner LA et al. *JAMA Network Open.*

S06.1X7A	ICD10	Traumatic cerebral edema with loss of consciousness of any duration with death due to brain injury prior to regaining consciousness, initial encounter
S06.1X7D	ICD10	Traumatic cerebral edema with loss of consciousness of any duration with death due to brain injury prior to regaining consciousness, subsequent encounter
S06.1X7S	ICD10	Traumatic cerebral edema with loss of consciousness of any duration with death due to brain injury prior to regaining consciousness, sequela
S06.1X8	ICD10	Traumatic cerebral edema w/LOC of any duration with death due to other causes prior to regaining consciousness
S06.1X8A	ICD10	Traumatic cerebral edema with loss of consciousness of any duration with death due to other cause prior to regaining consciousness, initial encounter
S06.1X8D	ICD10	Traumatic cerebral edema with loss of consciousness of any duration with death due to other cause prior to regaining consciousness, subsequent encounter
S06.1X8S	ICD10	Traumatic cerebral edema with loss of consciousness of any duration with death due to other cause prior to regaining consciousness, sequela
S06.1X9	ICD10	Traumatic cerebral edema w/LOC of unspecified duration
S06.1X9A	ICD10	Traumatic cerebral edema with loss of consciousness of unspecified duration, initial encounter
S06.1X9D	ICD10	Traumatic cerebral edema with loss of consciousness of unspecified duration, subsequent encounter
S06.1X9S	ICD10	Traumatic cerebral edema with loss of consciousness of unspecified duration, sequela
S06.2X	ICD10	Diffuse traumatic brain injury
S06.2X0	ICD10	Diffuse traumatic brain injury w/o LOC
S06.2X0A	ICD10	Diffuse traumatic brain injury without loss of consciousness, initial encounter
S06.2X0D	ICD10	Diffuse traumatic brain injury without loss of consciousness, subsequent encounter
S06.2X0S	ICD10	Diffuse traumatic brain injury without loss of consciousness, sequela
S06.2X1	ICD10	Diffuse traumatic brain injury w/LOC of 30 min or less
S06.2X1A	ICD10	Diffuse traumatic brain injury with loss of consciousness of 30 minutes or less, initial encounter
S06.2X1D	ICD10	Diffuse traumatic brain injury with loss of consciousness of 30 minutes or less, subsequent encounter
S06.2X1S	ICD10	Diffuse traumatic brain injury with loss of consciousness of 30 minutes or less, sequela
S06.2X2	ICD10	Diffuse traumatic brain injury w/LOC of 31-59 minutes
S06.2X2A	ICD10	Diffuse traumatic brain injury with loss of consciousness of 31 minutes to 59 minutes, initial encounter
S06.2X2D	ICD10	Diffuse traumatic brain injury with loss of consciousness of 31 minutes to 59 minutes, subsequent encounter
S06.2X2S	ICD10	Diffuse traumatic brain injury with loss of consciousness of 31 minutes to 59 minutes, sequela
S06.2X3	ICD10	Diffuse traumatic brain injury w/LOC of 1hr to 5 hrs 59 mins
S06.2X3A	ICD10	Diffuse traumatic brain injury with loss of consciousness of 1 hour to 5 hours 59 minutes, initial encounter
S06.2X3D	ICD10	Diffuse traumatic brain injury with loss of consciousness of 1 hour to 5 hours 59 minutes, subsequent encounter
S06.2X3S	ICD10	Diffuse traumatic brain injury with loss of consciousness of 1 hour to 5 hours 59 minutes, sequela
S06.2X4	ICD10	Diffuse traumatic brain injury w/LOC of 6-24 hrs

© 2023 Brenner LA et al. *JAMA Network Open.*

S06.2X4A	ICD10	Diffuse traumatic brain injury with loss of consciousness of 6 hours to 24 hours, initial encounter
S06.2X4D	ICD10	Diffuse traumatic brain injury with loss of consciousness of 6 hours to 24 hours, subsequent encounter
S06.2X4S	ICD10	Diffuse traumatic brain injury with loss of consciousness of 6 hours to 24 hours, sequela
S06.2X5	ICD10	Diffuse traumatic brain injury w/ LOC >24 hrs with return to pre-existing conscious level
S06.2X5A	ICD10	Diffuse traumatic brain injury with loss of consciousness greater than 24 hours with return to pre-existing conscious levels, initial encounter
S06.2X5D	ICD10	Diffuse traumatic brain injury with loss of consciousness greater than 24 hours with return to pre-existing conscious levels, subsequent encounter
S06.2X5S	ICD10	Diffuse traumatic brain injury with loss of consciousness greater than 24 hours with return to pre-existing conscious levels, sequela
S06.2X6	ICD10	Diffuse traumatic brain injury w/LOC >24 hrs w/o return to pre-existing conscious level with patient surviving
S06.2X6A	ICD10	Diffuse traumatic brain injury with loss of consciousness greater than 24 hours without return to pre-existing conscious level with patient surviving, initial encounter
S06.2X6D	ICD10	Diffuse traumatic brain injury with loss of consciousness greater than 24 hours without return to pre-existing conscious level with patient surviving, subsequent encounter
S06.2X6S	ICD10	Diffuse traumatic brain injury with loss of consciousness greater than 24 hours without return to pre-existing conscious level with patient surviving, sequela
S06.2X7	ICD10	Diffuse traumatic brain injury w/LOC of any duration with death due to brain injury prior to regaining consciousness
S06.2X7A	ICD10	Diffuse traumatic brain injury with loss of consciousness of any duration with death due to brain injury prior to regaining consciousness, initial encounter
S06.2X7D	ICD10	Diffuse traumatic brain injury with loss of consciousness of any duration with death due to brain injury prior to regaining consciousness, subsequent encounter
S06.2X7S	ICD10	Diffuse traumatic brain injury with loss of consciousness of any duration with death due to brain injury prior to regaining consciousness, sequela
S06.2X8	ICD10	Diffuse traumatic brain injury w/LOC of any duration with death due to other causes prior to regaining consciousness
S06.2X8A	ICD10	Diffuse traumatic brain injury with loss of consciousness of any duration with death due to other cause prior to regaining consciousness, initial encounter
S06.2X8D	ICD10	Diffuse traumatic brain injury with loss of consciousness of any duration with death due to other cause prior to regaining consciousness, subsequent encounter
S06.2X8S	ICD10	Diffuse traumatic brain injury with loss of consciousness of any duration with death due to other cause prior to regaining consciousness, sequela
S06.2X9	ICD10	Diffuse traumatic brain injury w/LOC of unspecified duration
S06.2X9A	ICD10	Diffuse traumatic brain injury with loss of consciousness of unspecified duration, initial encounter
S06.2X9D	ICD10	Diffuse traumatic brain injury with loss of consciousness of unspecified duration, subsequent encounter
S06.2X9S	ICD10	Diffuse traumatic brain injury with loss of consciousness of unspecified duration, sequela
S06.30	ICD10	Unspecified focal traumatic brain injury

© 2023 Brenner LA et al. *JAMA Network Open.*

S06.300	ICD10	Unspecified focal traumatic brain injury w/o LOC
S06.300A	ICD10	Unspecified focal traumatic brain injury without loss of consciousness, initial encounter
S06.300D	ICD10	Unspecified focal traumatic brain injury without loss of consciousness, subsequent encounter
S06.300S	ICD10	Unspecified focal traumatic brain injury without loss of consciousness, sequela
S06.301	ICD10	Unspecified focal traumatic brain injury w/LOC of 30 min or less
S06.301A	ICD10	Unspecified focal traumatic brain injury with loss of consciousness of 30 minutes or less, initial encounter
S06.301D	ICD10	Unspecified focal traumatic brain injury with loss of consciousness of 30 minutes or less, subsequent encounter
S06.301S	ICD10	Unspecified focal traumatic brain injury with loss of consciousness of 30 minutes or less, sequela
S06.302	ICD10	Unspecified focal traumatic brain injury w/LOC of 31-59 minutes
S06.302A	ICD10	Unspecified focal traumatic brain injury with loss of consciousness of 31 minutes to 59 minutes, initial encounter
S06.302D	ICD10	Unspecified focal traumatic brain injury with loss of consciousness of 31 minutes to 59 minutes, subsequent encounter
S06.302S	ICD10	Unspecified focal traumatic brain injury with loss of consciousness of 31 minutes to 59 minutes, sequela
S06.303	ICD10	Unspecified focal traumatic brain injury w/LOC of 1hr to 5 hrs 59 mins
S06.303A	ICD10	Unspecified focal traumatic brain injury with loss of consciousness of 1 hour to 5 hours 59 minutes, initial encounter
S06.303D	ICD10	Unspecified focal traumatic brain injury with loss of consciousness of 1 hour to 5 hours 59 minutes, subsequent encounter
S06.303S	ICD10	Unspecified focal traumatic brain injury with loss of consciousness of 1 hour to 5 hours 59 minutes, sequela
S06.304	ICD10	Unspecified focal traumatic brain injury w/LOC of 6-24 hrs
S06.304A	ICD10	Unspecified focal traumatic brain injury with loss of consciousness of 6 hours to 24 hours, initial encounter
S06.304D	ICD10	Unspecified focal traumatic brain injury with loss of consciousness of 6 hours to 24 hours, subsequent encounter
S06.304S	ICD10	Unspecified focal traumatic brain injury with loss of consciousness of 6 hours to 24 hours, sequela
S06.305	ICD10	Unspecified focal traumatic brain injury w/LOC >24 hrs with return to pre-existing conscious level
S06.305A	ICD10	Unspecified focal traumatic brain injury with loss of consciousness greater than 24 hours with return to pre-existing conscious level, initial encounter
S06.305D	ICD10	Unspecified focal traumatic brain injury with loss of consciousness greater than 24 hours with return to pre-existing conscious level, subsequent encounter
S06.305S	ICD10	Unspecified focal traumatic brain injury with loss of consciousness greater than 24 hours with return to pre-existing conscious level, sequela
S06.306	ICD10	Unspecified focal traumatic brain injury w/LOC >24 hrs w/o return to pre-existing conscious level with patient surviving
S06.306A	ICD10	Unspecified focal traumatic brain injury with loss of consciousness greater than 24 hours without return to pre-existing conscious level with patient surviving, initial encounter

© 2023 Brenner LA et al. *JAMA Network Open.*

S06.306D	ICD10	Unspecified focal traumatic brain injury with loss of consciousness greater than 24 hours without return to pre-existing conscious level with patient surviving, subsequent encounter
S06.306S	ICD10	Unspecified focal traumatic brain injury with loss of consciousness greater than 24 hours without return to pre-existing conscious level with patient surviving, sequela
S06.307	ICD10	Unspecified focal traumatic brain injury w/LOC of any duration with death due to brain injury prior to regaining consciousness
S06.307A	ICD10	Unspecified focal traumatic brain injury with loss of consciousness of any duration with death due to brain injury prior to regaining consciousness, initial encounter
S06.307D	ICD10	Unspecified focal traumatic brain injury with loss of consciousness of any duration with death due to brain injury prior to regaining consciousness, subsequent encounter
S06.307S	ICD10	Unspecified focal traumatic brain injury with loss of consciousness of any duration with death due to brain injury prior to regaining consciousness, sequela
S06.308	ICD10	Unspecified focal traumatic brain injury w/LOC of any duration with death due to other causes prior to regaining consciousness
S06.308A	ICD10	Unspecified focal traumatic brain injury with loss of consciousness of any duration with death due to other cause prior to regaining consciousness, initial encounter
S06.308D	ICD10	Unspecified focal traumatic brain injury with loss of consciousness of any duration with death due to other cause prior to regaining consciousness, subsequent encounter
S06.308S	ICD10	Unspecified focal traumatic brain injury with loss of consciousness of any duration with death due to other cause prior to regaining consciousness, sequela
S06.309	ICD10	Unspecified focal traumatic brain injury w/LOC of unspecified duration
S06.309A	ICD10	Unspecified focal traumatic brain injury with loss of consciousness of unspecified duration, initial encounter
S06.309D	ICD10	Unspecified focal traumatic brain injury with loss of consciousness of unspecified duration, subsequent encounter
S06.309S	ICD10	Unspecified focal traumatic brain injury with loss of consciousness of unspecified duration, sequela
S06.31	ICD10	Contusion and laceration of right cerebrum
S06.310	ICD10	Contusion and laceration of right cerebrum w/o LOC
S06.310A	ICD10	Contusion and laceration of right cerebrum without loss of consciousness, initial encounter
S06.310D	ICD10	Contusion and laceration of right cerebrum without loss of consciousness, subsequent encounter
S06.310S	ICD10	Contusion and laceration of right cerebrum without loss of consciousness, sequela
S06.311	ICD10	Contusion and laceration of right cerebrum w/LOC of 30 min or less
S06.311A	ICD10	Contusion and laceration of right cerebrum with loss of consciousness of 30 minutes or less, initial encounter
S06.311D	ICD10	Contusion and laceration of right cerebrum with loss of consciousness of 30 minutes or less, subsequent encounter
S06.311S	ICD10	Contusion and laceration of right cerebrum with loss of consciousness of 30 minutes or less, sequela
S06.312	ICD10	Contusion and laceration of right cerebrum w/LOC of 31-59 minutes
S06.312A	ICD10	Contusion and laceration of right cerebrum with loss of consciousness of 31 minutes to 59 minutes, initial encounter

© 2023 Brenner LA et al. *JAMA Network Open.*

S06.312D	ICD10	Contusion and laceration of right cerebrum with loss of consciousness of 31 minutes to 59 minutes, subsequent encounter
S06.312S	ICD10	Contusion and laceration of right cerebrum with loss of consciousness of 31 minutes to 59 minutes, sequela
S06.313	ICD10	Contusion and laceration of right cerebrum w/LOC of 1hr to 5 hrs 59 mins
S06.313A	ICD10	Contusion and laceration of right cerebrum with loss of consciousness of 1 hour to 5 hours 59 minutes, initial encounter
S06.313D	ICD10	Contusion and laceration of right cerebrum with loss of consciousness of 1 hour to 5 hours 59 minutes, subsequent encounter
S06.313S	ICD10	Contusion and laceration of right cerebrum with loss of consciousness of 1 hour to 5 hours 59 minutes, sequela
S06.314	ICD10	Contusion and laceration of right cerebrum w/LOC of 6-24 hrs
S06.314A	ICD10	Contusion and laceration of right cerebrum with loss of consciousness of 6 hours to 24 hours, initial encounter
S06.314D	ICD10	Contusion and laceration of right cerebrum with loss of consciousness of 6 hours to 24 hours, subsequent encounter
S06.314S	ICD10	Contusion and laceration of right cerebrum with loss of consciousness of 6 hours to 24 hours, sequela
S06.315	ICD10	Contusion and laceration of right cerebrum w/LOC >24 hrs with return to pre-existing conscious level
S06.315A	ICD10	Contusion and laceration of right cerebrum with loss of consciousness greater than 24 hours with return to pre-existing conscious level, initial encounter
S06.315D	ICD10	Contusion and laceration of right cerebrum with loss of consciousness greater than 24 hours with return to pre-existing conscious level, subsequent encounter
S06.315S	ICD10	Contusion and laceration of right cerebrum with loss of consciousness greater than 24 hours with return to pre-existing conscious level, sequela
S06.316	ICD10	Contusion and laceration of right cerebrum w/ LOC >24 hrs w/o return to pre-existing conscious level with patient surviving
S06.316A	ICD10	Contusion and laceration of right cerebrum with loss of consciousness greater than 24 hours without return to pre-existing conscious level with patient surviving, initial encounter
S06.316D	ICD10	Contusion and laceration of right cerebrum with loss of consciousness greater than 24 hours without return to pre-existing conscious level with patient surviving, subsequent encounter
S06.316S	ICD10	Contusion and laceration of right cerebrum with loss of consciousness greater than 24 hours without return to pre-existing conscious level with patient surviving, sequela
S06.317	ICD10	Contusion and laceration of right cerebrum w/LOC of any duration with death due to brain injury prior to regaining consciousness
S06.317A	ICD10	Contusion and laceration of right cerebrum with loss of consciousness of any duration with death due to brain injury prior to regaining consciousness, initial encounter
S06.317D	ICD10	Contusion and laceration of right cerebrum with loss of consciousness of any duration with death due to brain injury prior to regaining consciousness, subsequent encounter
S06.317S	ICD10	Contusion and laceration of right cerebrum with loss of consciousness of any duration with death due to brain injury prior to regaining consciousness, sequela

© 2023 Brenner LA et al. *JAMA Network Open.*

S06.318	ICD10	Contusion and laceration of right cerebrum w/LOC of any duration with death due to other causes prior to regaining consciousness
S06.318A	ICD10	Contusion and laceration of right cerebrum with loss of consciousness of any duration with death due to other cause prior to regaining consciousness, initial encounter
S06.318D	ICD10	Contusion and laceration of right cerebrum with loss of consciousness of any duration with death due to other cause prior to regaining consciousness, subsequent encounter
S06.318S	ICD10	Contusion and laceration of right cerebrum with loss of consciousness of any duration with death due to other cause prior to regaining consciousness, sequela
S06.319	ICD10	Contusion and laceration of right cerebrum w/LOC of unspecified duration
S06.319A	ICD10	Contusion and laceration of right cerebrum with loss of consciousness of unspecified duration, initial encounter
S06.319D	ICD10	Contusion and laceration of right cerebrum with loss of consciousness of unspecified duration, subsequent encounter
S06.319S	ICD10	Contusion and laceration of right cerebrum with loss of consciousness of unspecified duration, sequela
S06.32	ICD10	Contusion and laceration of left cerebrum
S06.320	ICD10	Contusion and laceration of left cerebrum w/o LOC
S06.320A	ICD10	Contusion and laceration of left cerebrum without loss of consciousness, initial encounter
S06.320D	ICD10	Contusion and laceration of left cerebrum without loss of consciousness, subsequent encounter
S06.320S	ICD10	Contusion and laceration of left cerebrum without loss of consciousness, sequela
S06.321	ICD10	Contusion and laceration of left cerebrum w/LOC of 30 min or less
S06.321A	ICD10	Contusion and laceration of left cerebrum with loss of consciousness of 30 minutes or less, initial encounter
S06.321D	ICD10	Contusion and laceration of left cerebrum with loss of consciousness of 30 minutes or less, subsequent encounter
S06.321S	ICD10	Contusion and laceration of left cerebrum with loss of consciousness of 30 minutes or less, sequela
S06.322	ICD10	Contusion and laceration of left cerebrum w/LOC of 31-59 minutes
S06.322A	ICD10	Contusion and laceration of left cerebrum with loss of consciousness of 31 minutes to 59 minutes, initial encounter
S06.322D	ICD10	Contusion and laceration of left cerebrum with loss of consciousness of 31 minutes to 59 minutes, subsequent encounter
S06.322S	ICD10	Contusion and laceration of left cerebrum with loss of consciousness of 31 minutes to 59 minutes, sequela
S06.323	ICD10	Contusion and laceration of left cerebrum w/LOC of 1hr to 5 hrs 59 mins
S06.323A	ICD10	Contusion and laceration of left cerebrum with loss of consciousness of 1 hour to 5 hours 59 minutes, initial encounter
S06.323D	ICD10	Contusion and laceration of left cerebrum with loss of consciousness of 1 hour to 5 hours 59 minutes, subsequent encounter
S06.323S	ICD10	Contusion and laceration of left cerebrum with loss of consciousness of 1 hour to 5 hours 59 minutes, sequela
S06.324	ICD10	Contusion and laceration of left cerebrum w/LOC of 6-24 hrs
S06.324A	ICD10	Contusion and laceration of left cerebrum with loss of consciousness of 6 hours to 24 hours, initial encounter

© 2023 Brenner LA et al. *JAMA Network Open.*

S06.324D	ICD10	Contusion and laceration of left cerebrum with loss of consciousness of 6 hours to 24 hours, subsequent encounter
S06.324S	ICD10	Contusion and laceration of left cerebrum with loss of consciousness of 6 hours to 24 hours, sequela
S06.325	ICD10	Contusion and laceration of left cerebrum w/LOC >24 hrs with return to pre-existing conscious level
S06.325A	ICD10	Contusion and laceration of left cerebrum with loss of consciousness greater than 24 hours with return to pre-existing conscious level, initial encounter
S06.325D	ICD10	Contusion and laceration of left cerebrum with loss of consciousness greater than 24 hours with return to pre-existing conscious level, subsequent encounter
S06.325S	ICD10	Contusion and laceration of left cerebrum with loss of consciousness greater than 24 hours with return to pre-existing conscious level, sequela
S06.326	ICD10	Contusion and laceration of left cerebrum w/LOC >24 hrs w/o return to pre-existing conscious level with patient surviving
S06.326A	ICD10	Contusion and laceration of left cerebrum with loss of consciousness greater than 24 hours without return to pre-existing conscious level with patient surviving, initial encounter
S06.326D	ICD10	Contusion and laceration of left cerebrum with loss of consciousness greater than 24 hours without return to pre-existing conscious level with patient surviving, subsequent encounter
S06.326S	ICD10	Contusion and laceration of left cerebrum with loss of consciousness greater than 24 hours without return to pre-existing conscious level with patient surviving, sequela
S06.327	ICD10	Contusion and laceration of left cerebrum w/LOC of any duration w/ death due to brain injury prior to regaining consciousness
S06.327A	ICD10	Contusion and laceration of left cerebrum with loss of consciousness of any duration with death due to brain injury prior to regaining consciousness, initial encounter
S06.327D	ICD10	Contusion and laceration of left cerebrum with loss of consciousness of any duration with death due to brain injury prior to regaining consciousness, subsequent encounter
S06.327S	ICD10	Contusion and laceration of left cerebrum with loss of consciousness of any duration with death due to brain injury prior to regaining consciousness, sequela
S06.328	ICD10	Contusion and laceration of left cerebrum w/LOC of any duration w/ death due to other causes prior to regaining consciousness
S06.328A	ICD10	Contusion and laceration of left cerebrum with loss of consciousness of any duration with death due to other cause prior to regaining consciousness, initial encounter
S06.328D	ICD10	Contusion and laceration of left cerebrum with loss of consciousness of any duration with death due to other cause prior to regaining consciousness, subsequent encounter
S06.328S	ICD10	Contusion and laceration of left cerebrum with loss of consciousness of any duration with death due to other cause prior to regaining consciousness, sequela
S06.329	ICD10	Contusion and laceration of left cerebrum w/LOC of unspecified duration
S06.329A	ICD10	Contusion and laceration of left cerebrum with loss of consciousness of unspecified duration, initial encounter
S06.329D	ICD10	Contusion and laceration of left cerebrum with loss of consciousness of unspecified duration, subsequent encounter
S06.329S	ICD10	Contusion and laceration of left cerebrum with loss of consciousness of unspecified duration, sequela

© 2023 Brenner LA et al. *JAMA Network Open.*

S06.33	ICD10	Contusion and laceration of cerebrum, unspecified
S06.330	ICD10	Contusion and laceration of cerebrum, unspecified, w/o LOC
S06.330A	ICD10	Contusion and laceration of cerebrum, unspecified, without loss of consciousness, initial encounter
S06.330D	ICD10	Contusion and laceration of cerebrum, unspecified, without loss of consciousness, subsequent encounter
S06.330S	ICD10	Contusion and laceration of cerebrum, unspecified, without loss of consciousness, sequela
S06.331	ICD10	Contusion and laceration of cerebrum, unspecified, w/LOC of 30 mins or less
S06.331A	ICD10	Contusion and laceration of cerebrum, unspecified, with loss of consciousness of 30 minutes or less, initial encounter
S06.331D	ICD10	Contusion and laceration of cerebrum, unspecified, with loss of consciousness of 30 minutes or less, subsequent encounter
S06.331S	ICD10	Contusion and laceration of cerebrum, unspecified, with loss of consciousness of 30 minutes or less, sequela
S06.332	ICD10	Contusion and laceration of cerebrum, unspecified, w/LOC of 31-59 mins
S06.332A	ICD10	Contusion and laceration of cerebrum, unspecified, with loss of consciousness of 31 minutes to 59 minutes, initial encounter
S06.332D	ICD10	Contusion and laceration of cerebrum, unspecified, with loss of consciousness of 31 minutes to 59 minutes, subsequent encounter
S06.332S	ICD10	Contusion and laceration of cerebrum, unspecified, with loss of consciousness of 31 minutes to 59 minutes, sequela
S06.333	ICD10	Contusion and laceration of cerebrum, unspecified, w/LOC of 1 hr to 5 hrs 59 mins
S06.333A	ICD10	Contusion and laceration of cerebrum, unspecified, with loss of consciousness of 1 hour to 5 hours 59 minutes, initial encounter
S06.333D	ICD10	Contusion and laceration of cerebrum, unspecified, with loss of consciousness of 1 hour to 5 hours 59 minutes, subsequent encounter
S06.333S	ICD10	Contusion and laceration of cerebrum, unspecified, with loss of consciousness of 1 hour to 5 hours 59 minutes, sequela
S06.334	ICD10	Contusion and laceration of cerebrum, unspecified, w/ LOC of 6 -24 hrs
S06.334A	ICD10	Contusion and laceration of cerebrum, unspecified, with loss of consciousness of 6 hours to 24 hours, initial encounter
S06.334D	ICD10	Contusion and laceration of cerebrum, unspecified, with loss of consciousness of 6 hours to 24 hours, subsequent encounter
S06.334S	ICD10	Contusion and laceration of cerebrum, unspecified, with loss of consciousness of 6 hours to 24 hours, sequela
S06.335	ICD10	Contusion and laceration of cerebrum, unspecified, w/LOC > 24 hrs with return to pre-existing consciousness level
S06.335A	ICD10	Contusion and laceration of cerebrum, unspecified, with loss of consciousness greater than 24 hours with return to pre-existing conscious level, initial encounter
S06.335D	ICD10	Contusion and laceration of cerebrum, unspecified, with loss of consciousness greater than 24 hours with return to pre-existing conscious level, subsequent encounter

© 2023 Brenner LA et al. *JAMA Network Open.*

S06.335S	ICD10	Contusion and laceration of cerebrum, unspecified, with loss of consciousness greater than 24 hours with return to pre-existing conscious level, sequela
S06.336	ICD10	Contusion and laceration of cerebrum, unspecified, w/LOC >24 hrs w/o return to pre-existing conscious level with patient surviving
S06.336A	ICD10	Contusion and laceration of cerebrum, unspecified, with loss of consciousness greater than 24 hours without return to pre-existing conscious level with patient surviving, initial encounter
S06.336D	ICD10	Contusion and laceration of cerebrum, unspecified, with loss of consciousness greater than 24 hours without return to pre-existing conscious level with patient surviving, subsequent encounter
S06.336S	ICD10	Contusion and laceration of cerebrum, unspecified, with loss of consciousness greater than 24 hours without return to pre-existing conscious level with patient surviving, sequela
S06.337	ICD10	Contusion and laceration of cerebrum, unspecified, w/LOC of any duration w/ death due to brain injury prior to regaining consciousness
S06.337A	ICD10	Contusion and laceration of cerebrum, unspecified, with loss of consciousness of any duration with death due to brain injury prior to regaining consciousness, initial encounter
S06.337D	ICD10	Contusion and laceration of cerebrum, unspecified, with loss of consciousness of any duration with death due to brain injury prior to regaining consciousness, subsequent encounter
S06.337S	ICD10	Contusion and laceration of cerebrum, unspecified, with loss of consciousness of any duration with death due to brain injury prior to regaining consciousness, sequela
S06.338	ICD10	Contusion and laceration of cerebrum, unspecified, w/LOC of any duration with death due to other cause prior to regaining conscious
S06.338A	ICD10	Contusion and laceration of cerebrum, unspecified, with loss of consciousness of any duration with death due to other cause prior to regaining consciousness, initial encounter
S06.338D	ICD10	Contusion and laceration of cerebrum, unspecified, with loss of consciousness of any duration with death due to other cause prior to regaining consciousness, subsequent encounter
S06.338S	ICD10	Contusion and laceration of cerebrum, unspecified, with loss of consciousness of any duration with death due to other cause prior to regaining consciousness, sequela
S06.339	ICD10	Contusion and laceration of cerebrum, unspecified, w/LOC of unspecified duration
S06.339A	ICD10	Contusion and laceration of cerebrum, unspecified, with loss of consciousness of unspecified duration, initial encounter
S06.339D	ICD10	Contusion and laceration of cerebrum, unspecified, with loss of consciousness of unspecified duration, subsequent encounter
S06.339S	ICD10	Contusion and laceration of cerebrum, unspecified, with loss of consciousness of unspecified duration, sequela
S06.34	ICD10	Traumatic hemorrhage of right cerebrum
S06.340	ICD10	Traumatic hemorrhage of right cerebrum w/o LOC
S06.340A	ICD10	Traumatic hemorrhage of right cerebrum without loss of consciousness, initial encounter
S06.340D	ICD10	Traumatic hemorrhage of right cerebrum without loss of consciousness, subsequent encounter
S06.340S	ICD10	Traumatic hemorrhage of right cerebrum without loss of consciousness, sequela
S06.341	ICD10	Traumatic hemorrhage of right cerebrum w/LOC of 30 mins or less

© 2023 Brenner LA et al. *JAMA Network Open.*

S06.341A	ICD10	Traumatic hemorrhage of right cerebrum with loss of consciousness of 30 minutes or less, initial encounter
S06.341D	ICD10	Traumatic hemorrhage of right cerebrum with loss of consciousness of 30 minutes or less, subsequent encounter
S06.341S	ICD10	Traumatic hemorrhage of right cerebrum with loss of consciousness of 30 minutes or less, sequela
S06.342	ICD10	Traumatic hemorrhage of right cerebrum w/LOC of 31-59 mins
S06.342A	ICD10	Traumatic hemorrhage of right cerebrum with loss of consciousness of 31 minutes to 59 minutes, initial encounter
S06.342D	ICD10	Traumatic hemorrhage of right cerebrum with loss of consciousness of 31 minutes to 59 minutes, subsequent encounter
S06.342S	ICD10	Traumatic hemorrhage of right cerebrum with loss of consciousness of 31 minutes to 59 minutes, sequela
S06.343	ICD10	Traumatic hemorrhage of right cerebrum w/LOC of 1 hr to 5 hrs 59 mins
S06.343A	ICD10	Traumatic hemorrhage of right cerebrum with loss of consciousness of 1 hours to 5 hours 59 minutes, initial encounter
S06.343D	ICD10	Traumatic hemorrhage of right cerebrum with loss of consciousness of 1 hours to 5 hours 59 minutes, subsequent encounter
S06.343S	ICD10	Traumatic hemorrhage of right cerebrum with loss of consciousness of 1 hours to 5 hours 59 minutes, sequela
S06.344	ICD10	Traumatic hemorrhage of right cerebrum with LOC of 6 -24 hrs
S06.344A	ICD10	Traumatic hemorrhage of right cerebrum with loss of consciousness of 6 hours to 24 hours, initial encounter
S06.344D	ICD10	Traumatic hemorrhage of right cerebrum with loss of consciousness of 6 hours to 24 hours, subsequent encounter
S06.344S	ICD10	Traumatic hemorrhage of right cerebrum with loss of consciousness of 6 hours to 24 hours, sequela
S06.345	ICD10	Traumatic hemorrhage of right cerebrum w/LOC > 24 hrs with return to pre-existing consciousness level
S06.345A	ICD10	Traumatic hemorrhage of right cerebrum with loss of consciousness greater than 24 hours with return to pre-existing conscious level, initial encounter
S06.345D	ICD10	Traumatic hemorrhage of right cerebrum with loss of consciousness greater than 24 hours with return to pre-existing conscious level, subsequent encounter
S06.345S	ICD10	Traumatic hemorrhage of right cerebrum with loss of consciousness greater than 24 hours with return to pre-existing conscious level, sequela
S06.346	ICD10	Traumatic hemorrhage of right cerebrum w/LOC >24 hrs w/o return to pre-existing conscious level with patient surviving
S06.346A	ICD10	Traumatic hemorrhage of right cerebrum with loss of consciousness greater than 24 hours without return to pre-existing conscious level with patient surviving, initial encounter
S06.346D	ICD10	Traumatic hemorrhage of right cerebrum with loss of consciousness greater than 24 hours without return to pre-existing conscious level with patient surviving, subsequent encounter
S06.346S	ICD10	Traumatic hemorrhage of right cerebrum with loss of consciousness greater than 24 hours without return to pre-existing conscious level with patient surviving, sequela
S06.347	ICD10	Traumatic hemorrhage of right cerebrum w/LOC of any duration with death due to brain injury prior to regaining consciousness

© 2023 Brenner LA et al. *JAMA Network Open.*

S06.347A	ICD10	Traumatic hemorrhage of right cerebrum with loss of consciousness of any duration with death due to brain injury prior to regaining consciousness, initial encounter
S06.347D	ICD10	Traumatic hemorrhage of right cerebrum with loss of consciousness of any duration with death due to brain injury prior to regaining consciousness, subsequent encounter
S06.347S	ICD10	Traumatic hemorrhage of right cerebrum with loss of consciousness of any duration with death due to brain injury prior to regaining consciousness, sequela
S06.348	ICD10	Traumatic hemorrhage of right cerebrum w/LOC of any duration with death due to other cause prior to regaining consciousness
S06.348A	ICD10	Traumatic hemorrhage of right cerebrum with loss of consciousness of any duration with death due to other cause prior to regaining consciousness, initial encounter
S06.348D	ICD10	Traumatic hemorrhage of right cerebrum with loss of consciousness of any duration with death due to other cause prior to regaining consciousness, subsequent encounter
S06.348S	ICD10	Traumatic hemorrhage of right cerebrum with loss of consciousness of any duration with death due to other cause prior to regaining consciousness, sequela
S06.349	ICD10	Traumatic hemorrhage of right cerebrum w/LOC of unspecified duration
S06.349A	ICD10	Traumatic hemorrhage of right cerebrum with loss of consciousness of unspecified duration, initial encounter
S06.349D	ICD10	Traumatic hemorrhage of right cerebrum with loss of consciousness of unspecified duration, subsequent encounter
S06.349S	ICD10	Traumatic hemorrhage of right cerebrum with loss of consciousness of unspecified duration, sequela
S06.35	ICD10	Traumatic hemorrhage of left cerebrum
S06.350	ICD10	Traumatic hemorrhage of left cerebrum w/o LOC
S06.350A	ICD10	Traumatic hemorrhage of left cerebrum without loss of consciousness, initial encounter
S06.350D	ICD10	Traumatic hemorrhage of left cerebrum without loss of consciousness, subsequent encounter
S06.350S	ICD10	Traumatic hemorrhage of left cerebrum without loss of consciousness, sequela
S06.351	ICD10	Traumatic hemorrhage of left cerebrum w/LOC of 30 mins or less
S06.351A	ICD10	Traumatic hemorrhage of left cerebrum with loss of consciousness of 30 minutes or less, initial encounter
S06.351D	ICD10	Traumatic hemorrhage of left cerebrum with loss of consciousness of 30 minutes or less, subsequent encounter
S06.351S	ICD10	Traumatic hemorrhage of left cerebrum with loss of consciousness of 30 minutes or less, sequela
S06.352	ICD10	Traumatic hemorrhage of left cerebrum w/LOC of 31-59 mins
S06.352A	ICD10	Traumatic hemorrhage of left cerebrum with loss of consciousness of 31 minutes to 59 minutes, initial encounter
S06.352D	ICD10	Traumatic hemorrhage of left cerebrum with loss of consciousness of 31 minutes to 59 minutes, subsequent encounter
S06.352S	ICD10	Traumatic hemorrhage of left cerebrum with loss of consciousness of 31 minutes to 59 minutes, sequela
S06.353	ICD10	Traumatic hemorrhage of left cerebrum, unspecified, LOC of 1 hr to 5 hrs 59 mins
S06.353A	ICD10	Traumatic hemorrhage of left cerebrum with loss of consciousness of 1 hours to 5 hours 59 minutes, initial encounter

© 2023 Brenner LA et al. *JAMA Network Open.*

S06.353D	ICD10	Traumatic hemorrhage of left cerebrum with loss of consciousness of 1 hours to 5 hours 59 minutes, subsequent encounter
S06.353S	ICD10	Traumatic hemorrhage of left cerebrum with loss of consciousness of 1 hours to 5 hours 59 minutes, sequela
S06.354	ICD10	Traumatic hemorrhage of left cerebrum w/LOC of 6 -24 hrs
S06.354A	ICD10	Traumatic hemorrhage of left cerebrum with loss of consciousness of 6 hours to 24 hours, initial encounter
S06.354D	ICD10	Traumatic hemorrhage of left cerebrum with loss of consciousness of 6 hours to 24 hours, subsequent encounter
S06.354S	ICD10	Traumatic hemorrhage of left cerebrum with loss of consciousness of 6 hours to 24 hours, sequela
S06.355	ICD10	Traumatic hemorrhage of left cerebrum w/LOC >24 hrs with return to pre-existing consciousness level
S06.355A	ICD10	Traumatic hemorrhage of left cerebrum with loss of consciousness greater than 24 hours with return to pre-existing conscious level, initial encounter
S06.355D	ICD10	Traumatic hemorrhage of left cerebrum with loss of consciousness greater than 24 hours with return to pre-existing conscious level, subsequent encounter
S06.355S	ICD10	Traumatic hemorrhage of left cerebrum with loss of consciousness greater than 24 hours with return to pre-existing conscious level, sequela
S06.356	ICD10	Traumatic hemorrhage of left cerebrum w/LOC >24 hrs w/o return to pre-existing conscious level with patient surviving
S06.356A	ICD10	Traumatic hemorrhage of left cerebrum with loss of consciousness greater than 24 hours without return to pre-existing conscious level with patient surviving, initial encounter
S06.356D	ICD10	Traumatic hemorrhage of left cerebrum with loss of consciousness greater than 24 hours without return to pre-existing conscious level with patient surviving, subsequent encounter
S06.356S	ICD10	Traumatic hemorrhage of left cerebrum with loss of consciousness greater than 24 hours without return to pre-existing conscious level with patient surviving, sequela
S06.357	ICD10	Traumatic hemorrhage of left cerebrum w/LOC of any duration with death due to brain injury prior to regaining consciousness
S06.357A	ICD10	Traumatic hemorrhage of left cerebrum with loss of consciousness of any duration with death due to brain injury prior to regaining consciousness, initial encounter
S06.357D	ICD10	Traumatic hemorrhage of left cerebrum with loss of consciousness of any duration with death due to brain injury prior to regaining consciousness, subsequent encounter
S06.357S	ICD10	Traumatic hemorrhage of left cerebrum with loss of consciousness of any duration with death due to brain injury prior to regaining consciousness, sequela
S06.358	ICD10	Traumatic hemorrhage of left cerebrum w/LOC of any duration with death due to other cause prior to regaining consciousness
S06.358A	ICD10	Traumatic hemorrhage of left cerebrum with loss of consciousness of any duration with death due to other cause prior to regaining consciousness, initial encounter
S06.358D	ICD10	Traumatic hemorrhage of left cerebrum with loss of consciousness of any duration with death due to other cause prior to regaining consciousness, subsequent encounter
S06.358S	ICD10	Traumatic hemorrhage of left cerebrum with loss of consciousness of any duration with death due to other cause prior to regaining consciousness, sequela

© 2023 Brenner LA et al. *JAMA Network Open.*

S06.359	ICD10	Traumatic hemorrhage of left cerebrum, w/ LOC of unspecified duration
S06.359A	ICD10	Traumatic hemorrhage of left cerebrum with loss of consciousness of unspecified duration, initial encounter
S06.359D	ICD10	Traumatic hemorrhage of left cerebrum with loss of consciousness of unspecified duration, subsequent encounter
S06.359S	ICD10	Traumatic hemorrhage of left cerebrum with loss of consciousness of unspecified duration, sequela
S06.36	ICD10	Traumatic hemorrhage of cerebrum, unspecified
S06.360	ICD10	Traumatic hemorrhage of cerebrum, unspecified, w/o LOC
S06.360A	ICD10	Traumatic hemorrhage of cerebrum, unspecified, without loss of consciousness, initial encounter
S06.360D	ICD10	Traumatic hemorrhage of cerebrum, unspecified, without loss of consciousness, subsequent encounter
S06.360S	ICD10	Traumatic hemorrhage of cerebrum, unspecified, without loss of consciousness, sequela
S06.361	ICD10	Traumatic hemorrhage of cerebrum, unspecified, w/ LOC of 30 mins or less
S06.361A	ICD10	Traumatic hemorrhage of cerebrum, unspecified, with loss of consciousness of 30 minutes or less, initial encounter
S06.361D	ICD10	Traumatic hemorrhage of cerebrum, unspecified, with loss of consciousness of 30 minutes or less, subsequent encounter
S06.361S	ICD10	Traumatic hemorrhage of cerebrum, unspecified, with loss of consciousness of 30 minutes or less, sequela
S06.362	ICD10	Traumatic hemorrhage of cerebrum, unspecified, w/ LOC of 31-59 mins
S06.362A	ICD10	Traumatic hemorrhage of cerebrum, unspecified, with loss of consciousness of 31 minutes to 59 minutes, initial encounter
S06.362D	ICD10	Traumatic hemorrhage of cerebrum, unspecified, with loss of consciousness of 31 minutes to 59 minutes, subsequent encounter
S06.362S	ICD10	Traumatic hemorrhage of cerebrum, unspecified, with loss of consciousness of 31 minutes to 59 minutes, sequela
S06.363	ICD10	Traumatic hemorrhage of cerebrum, unspecified, LOC of 1 hr to 5 hrs 59 mins
S06.363A	ICD10	Traumatic hemorrhage of cerebrum, unspecified, with loss of consciousness of 1 hours to 5 hours 59 minutes, initial encounter
S06.363D	ICD10	Traumatic hemorrhage of cerebrum, unspecified, with loss of consciousness of 1 hours to 5 hours 59 minutes, subsequent encounter
S06.363S	ICD10	Traumatic hemorrhage of cerebrum, unspecified, with loss of consciousness of 1 hours to 5 hours 59 minutes, sequela
S06.364	ICD10	Traumatic hemorrhage of cerebrum, unspecified, w/LOC of 6 -24 hrs
S06.364A	ICD10	Traumatic hemorrhage of cerebrum, unspecified, with loss of consciousness of 6 hours to 24 hours, initial encounter
S06.364D	ICD10	Traumatic hemorrhage of cerebrum, unspecified, with loss of consciousness of 6 hours to 24 hours, subsequent encounter
S06.364S	ICD10	Traumatic hemorrhage of cerebrum, unspecified, with loss of consciousness of 6 hours to 24 hours, sequela
S06.365	ICD10	Traumatic hemorrhage of cerebrum, unspecified, w/LOC > 24 hrs with return to pre-existing conscioueness level

© 2023 Brenner LA et al. *JAMA Network Open.*

S06.365A	ICD10	Traumatic hemorrhage of cerebrum, unspecified, with loss of consciousness greater than 24 hours with return to pre-existing conscious level, initial encounter
S06.365D	ICD10	Traumatic hemorrhage of cerebrum, unspecified, with loss of consciousness greater than 24 hours with return to pre-existing conscious level, subsequent encounter
S06.365S	ICD10	Traumatic hemorrhage of cerebrum, unspecified, with loss of consciousness greater than 24 hours with return to pre-existing conscious level, sequela
S06.366	ICD10	Traumatic hemorrhage of cerebrum, unspecified, LOC >24 hrs w/o return to pre-existing conscious level with patient surviving
S06.366A	ICD10	Traumatic hemorrhage of cerebrum, unspecified, with loss of consciousness greater than 24 hours without return to pre-existing conscious level with patient surviving, initial encounter
S06.366D	ICD10	Traumatic hemorrhage of cerebrum, unspecified, with loss of consciousness greater than 24 hours without return to pre-existing conscious level with patient surviving, subsequent encounter
S06.366S	ICD10	Traumatic hemorrhage of cerebrum, unspecified, with loss of consciousness greater than 24 hours without return to pre-existing conscious level with patient surviving, sequela
S06.367	ICD10	Traumatic hemorrhage of cerebrum, unspecified, w/LOC of any duration with death due to brain injury prior to regaining consciousness
S06.367A	ICD10	Traumatic hemorrhage of cerebrum, unspecified, with loss of consciousness of any duration with death due to brain injury prior to regaining consciousness, initial encounter
S06.367D	ICD10	Traumatic hemorrhage of cerebrum, unspecified, with loss of consciousness of any duration with death due to brain injury prior to regaining consciousness, subsequent encounter
S06.367S	ICD10	Traumatic hemorrhage of cerebrum, unspecified, with loss of consciousness of any duration with death due to brain injury prior to regaining consciousness, sequela
S06.368	ICD10	Traumatic hemorrhage of cerebrum, unspecified, w/LOC of any duration with death due to other cause prior to regaining consciousness
S06.368A	ICD10	Traumatic hemorrhage of cerebrum, unspecified, with loss of consciousness of any duration with death due to other cause prior to regaining consciousness, initial encounter
S06.368D	ICD10	Traumatic hemorrhage of cerebrum, unspecified, with loss of consciousness of any duration with death due to other cause prior to regaining consciousness, subsequent encounter
S06.368S	ICD10	Traumatic hemorrhage of cerebrum, unspecified, with loss of consciousness of any duration with death due to other cause prior to regaining consciousness, sequela
S06.369	ICD10	Traumatic hemorrhage of cerebrum, unspecified, w/ LOC of unspecified duration
S06.369A	ICD10	Traumatic hemorrhage of cerebrum, unspecified, with loss of consciousness of unspecified duration, initial encounter
S06.369D	ICD10	Traumatic hemorrhage of cerebrum, unspecified, with loss of consciousness of unspecified duration, subsequent encounter
S06.369S	ICD10	Traumatic hemorrhage of cerebrum, unspecified, with loss of consciousness of unspecified duration, sequela
S06.37	ICD10	Contusion, laceration, and hemorrhage of cerebellum
S06.370	ICD10	Contusion, laceration, and hemorrhage of cerebellum w/o LOC
S06.370A	ICD10	Contusion, laceration, and hemorrhage of cerebellum without loss of consciousness, initial encounter

© 2023 Brenner LA et al. *JAMA Network Open.*

S06.370D	ICD10	Contusion, laceration, and hemorrhage of cerebellum without loss of consciousness, subsequent encounter
S06.370S	ICD10	Contusion, laceration, and hemorrhage of cerebellum without loss of consciousness, sequela
S06.371	ICD10	Contusion, laceration, and hemorrhage of cerebellum w/LOC of 30 mins or less
S06.371A	ICD10	Contusion, laceration, and hemorrhage of cerebellum with loss of consciousness of 30 minutes or less, initial encounter
S06.371D	ICD10	Contusion, laceration, and hemorrhage of cerebellum with loss of consciousness of 30 minutes or less, subsequent encounter
S06.371S	ICD10	Contusion, laceration, and hemorrhage of cerebellum with loss of consciousness of 30 minutes or less, sequela
S06.372	ICD10	Contusion, laceration, and hemorrhage of cerebellum w/LOC of 31 to 59 mins
S06.372A	ICD10	Contusion, laceration, and hemorrhage of cerebellum with loss of consciousness of 31 minutes to 59 minutes, initial encounter
S06.372D	ICD10	Contusion, laceration, and hemorrhage of cerebellum with loss of consciousness of 31 minutes to 59 minutes, subsequent encounter
S06.372S	ICD10	Contusion, laceration, and hemorrhage of cerebellum with loss of consciousness of 31 minutes to 59 minutes, sequela
S06.373	ICD10	Contusion, laceration, and hemorrhage of cerebellum w/LOC of 1 hr to 5 hrs 59 mins
S06.373A	ICD10	Contusion, laceration, and hemorrhage of cerebellum with loss of consciousness of 1 hour to 5 hours 59 minutes, initial encounter
S06.373D	ICD10	Contusion, laceration, and hemorrhage of cerebellum with loss of consciousness of 1 hour to 5 hours 59 minutes, subsequent encounter
S06.373S	ICD10	Contusion, laceration, and hemorrhage of cerebellum with loss of consciousness of 1 hour to 5 hours 59 minutes, sequela
S06.374	ICD10	Contusion, laceration, and hemorrhage of cerebellum w/LOC of 6-24 hrs
S06.374A	ICD10	Contusion, laceration, and hemorrhage of cerebellum with loss of consciousness of 6 hours to 24 hours, initial encounter
S06.374D	ICD10	Contusion, laceration, and hemorrhage of cerebellum with loss of consciousness of 6 hours to 24 hours, subsequent encounter
S06.374S	ICD10	Contusion, laceration, and hemorrhage of cerebellum with loss of consciousness of 6 hours to 24 hours, sequela
S06.375	ICD10	Contusion, laceration, and hemorrhage of cerebellum w/LOC > 24hrs with return to pre-existing conscious level
S06.375A	ICD10	Contusion, laceration, and hemorrhage of cerebellum with loss of consciousness greater than 24 hours with return to pre-existing conscious level, initial encounter
S06.375D	ICD10	Contusion, laceration, and hemorrhage of cerebellum with loss of consciousness greater than 24 hours with return to pre-existing conscious level, subsequent encounter
S06.375S	ICD10	Contusion, laceration, and hemorrhage of cerebellum with loss of consciousness greater than 24 hours with return to pre-existing conscious level, sequela
S06.376	ICD10	Contusion, laceration, and hemorrhage of cerebellum w/LOC <24 hrs w/o return to pre-existing conscious level with patient surviving

© 2023 Brenner LA et al. *JAMA Network Open.*

S06.376A	ICD10	Contusion, laceration, and hemorrhage of cerebellum with loss of consciousness greater than 24 hours without return to pre-existing conscious level with patient surviving, initial encounter
S06.376D	ICD10	Contusion, laceration, and hemorrhage of cerebellum with loss of consciousness greater than 24 hours without return to pre-existing conscious level with patient surviving, subsequent encounter
S06.376S	ICD10	Contusion, laceration, and hemorrhage of cerebellum with loss of consciousness greater than 24 hours without return to pre-existing conscious level with patient surviving, sequela
S06.377	ICD10	Contusion, laceration, and hemorrhage of cerebellum w/LOC of any duration w/death due to brain injury prior to regaining consciousness
S06.377A	ICD10	Contusion, laceration, and hemorrhage of cerebellum with loss of consciousness of any duration with death due to brain injury prior to regaining consciousness, initial encounter
S06.377D	ICD10	Contusion, laceration, and hemorrhage of cerebellum with loss of consciousness of any duration with death due to brain injury prior to regaining consciousness, subsequent encounter
S06.377S	ICD10	Contusion, laceration, and hemorrhage of cerebellum with loss of consciousness of any duration with death due to brain injury prior to regaining consciousness, sequela
S06.378	ICD10	Contusion, laceration, and hemorrhage of cerebellum w/LOC of any duration w/ death due to other cause prior to regaining consciousness
S06.378A	ICD10	Contusion, laceration, and hemorrhage of cerebellum with loss of consciousness of any duration with death due to other cause prior to regaining consciousness, initial encounter
S06.378D	ICD10	Contusion, laceration, and hemorrhage of cerebellum with loss of consciousness of any duration with death due to other cause prior to regaining consciousness, subsequent encounter
S06.378S	ICD10	Contusion, laceration, and hemorrhage of cerebellum with loss of consciousness of any duration with death due to other cause prior to regaining consciousness, sequela
S06.379	ICD10	Contusion, laceration, and hemorrhage of cerebellum w/LOC of unspecified duration
S06.379A	ICD10	Contusion, laceration, and hemorrhage of cerebellum with loss of consciousness of unspecified duration, initial encounter
S06.379D	ICD10	Contusion, laceration, and hemorrhage of cerebellum with loss of consciousness of unspecified duration, subsequent encounter
S06.379S	ICD10	Contusion, laceration, and hemorrhage of cerebellum with loss of consciousness of unspecified duration, sequela
S06.38	ICD10	Contusion, laceration, and hemorrhage of brainstem
S06.380	ICD10	Contusion, laceration, and hemorrhage of brainstem w/o LOC
S06.380A	ICD10	Contusion, laceration, and hemorrhage of brainstem without loss of consciousness, initial encounter
S06.380D	ICD10	Contusion, laceration, and hemorrhage of brainstem without loss of consciousness, subsequent encounter
S06.380S	ICD10	Contusion, laceration, and hemorrhage of brainstem without loss of consciousness, sequela
S06.381	ICD10	Contusion, laceration, and hemorrhage of brainstem w/LOC of 30 minutes or less
S06.381A	ICD10	Contusion, laceration, and hemorrhage of brainstem with loss of consciousness of 30 minutes or less, initial encounter

© 2023 Brenner LA et al. *JAMA Network Open.*

S06.381D	ICD10	Contusion, laceration, and hemorrhage of brainstem with loss of consciousness of 30 minutes or less, subsequent encounter
S06.381S	ICD10	Contusion, laceration, and hemorrhage of brainstem with loss of consciousness of 30 minutes or less, sequela
S06.382	ICD10	Contusion, laceration, and hemorrhage of brainstem w/LOC of 31 to 59 mins
S06.382A	ICD10	Contusion, laceration, and hemorrhage of brainstem with loss of consciousness of 31 minutes to 59 minutes, initial encounter
S06.382D	ICD10	Contusion, laceration, and hemorrhage of brainstem with loss of consciousness of 31 minutes to 59 minutes, subsequent encounter
S06.382S	ICD10	Contusion, laceration, and hemorrhage of brainstem with loss of consciousness of 31 minutes to 59 minutes, sequela
S06.383	ICD10	Contusion, laceration, and hemorrhage of brainstem w/LOC of 1 hr to 5 hrs 59 mins
S06.383A	ICD10	Contusion, laceration, and hemorrhage of brainstem with loss of consciousness of 1 hour to 5 hours 59 minutes, initial encounter
S06.383D	ICD10	Contusion, laceration, and hemorrhage of brainstem with loss of consciousness of 1 hour to 5 hours 59 minutes, subsequent encounter
S06.383S	ICD10	Contusion, laceration, and hemorrhage of brainstem with loss of consciousness of 1 hour to 5 hours 59 minutes, sequela
S06.384	ICD10	Contusion, laceration, and hemorrhage of brainstem w/LOC of 6-24 hrs
S06.384A	ICD10	Contusion, laceration, and hemorrhage of brainstem with loss of consciousness of 6 hours to 24 hours, initial encounter
S06.384D	ICD10	Contusion, laceration, and hemorrhage of brainstem with loss of consciousness of 6 hours to 24 hours, subsequent encounter
S06.384S	ICD10	Contusion, laceration, and hemorrhage of brainstem with loss of consciousness of 6 hours to 24 hours, sequela
S06.385	ICD10	Contusion, laceration, and hemorrhage of brainstem w/LOC > 24hrs with return to pre-existing conscious level
S06.385A	ICD10	Contusion, laceration, and hemorrhage of brainstem with loss of consciousness greater than 24 hours with return to pre-existing conscious level, initial encounter
S06.385D	ICD10	Contusion, laceration, and hemorrhage of brainstem with loss of consciousness greater than 24 hours with return to pre-existing conscious level, subsequent encounter
S06.385S	ICD10	Contusion, laceration, and hemorrhage of brainstem with loss of consciousness greater than 24 hours with return to pre-existing conscious level, sequela
S06.386	ICD10	Contusion, laceration, and hemorrhage of brainstem w/LOC > 24 hrs w/o return to pre-existing conscious level with patient surviving
S06.386A	ICD10	Contusion, laceration, and hemorrhage of brainstem with loss of consciousness greater than 24 hours without return to pre-existing conscious level with patient surviving, initial encounter
S06.386D	ICD10	Contusion, laceration, and hemorrhage of brainstem with loss of consciousness greater than 24 hours without return to pre-existing conscious level with patient surviving, subsequent encounter
S06.386S	ICD10	Contusion, laceration, and hemorrhage of brainstem with loss of consciousness greater than 24 hours without return to pre-existing conscious level with patient surviving, sequela

© 2023 Brenner LA et al. *JAMA Network Open.*

S06.387	ICD10	Contusion, laceration, and hemorrhage of brainstem w/LOC of any duration w/ death due to brain injury prior to regaining consciousness
S06.387A	ICD10	Contusion, laceration, and hemorrhage of brainstem with loss of consciousness of any duration with death due to brain injury prior to regaining consciousness, initial encounter
S06.387D	ICD10	Contusion, laceration, and hemorrhage of brainstem with loss of consciousness of any duration with death due to brain injury prior to regaining consciousness, subsequent encounter
S06.387S	ICD10	Contusion, laceration, and hemorrhage of brainstem with loss of consciousness of any duration with death due to brain injury prior to regaining consciousness, sequela
S06.388	ICD10	Contusion, laceration, and hemorrhage of brainstem with loss of consciousness of any duration w/death due to other cause prior to regaining consciousness
S06.388A	ICD10	Contusion, laceration, and hemorrhage of brainstem with loss of consciousness of any duration with death due to other cause prior to regaining consciousness, initial encounter
S06.388D	ICD10	Contusion, laceration, and hemorrhage of brainstem with loss of consciousness of any duration with death due to other cause prior to regaining consciousness, subsequent encounter
S06.388S	ICD10	Contusion, laceration, and hemorrhage of brainstem with loss of consciousness of any duration with death due to other cause prior to regaining consciousness, sequela
S06.389	ICD10	Contusion, laceration, and hemorrhage of brainstem w/LOC of unspecified duration
S06.389A	ICD10	Contusion, laceration, and hemorrhage of brainstem with loss of consciousness of unspecified duration, initial encounter
S06.389D	ICD10	Contusion, laceration, and hemorrhage of brainstem with loss of consciousness of unspecified duration, subsequent encounter
S06.389S	ICD10	Contusion, laceration, and hemorrhage of brainstem with loss of consciousness of unspecified duration, sequela
S06.4X	ICD10	Epidural hemorrhage
S06.4X0	ICD10	Epidural hemorrhage w/o LOC
S06.4X0A	ICD10	Epidural hemorrhage without loss of consciousness, initial encounter
S06.4X0D	ICD10	Epidural hemorrhage without loss of consciousness, subsequent encounter
S06.4X0S	ICD10	Epidural hemorrhage without loss of consciousness, sequela
S06.4X1	ICD10	Epidural hemorrhage w/LOC of 30 mins or less
S06.4X1A	ICD10	Epidural hemorrhage with loss of consciousness of 30 minutes or less, initial encounter
S06.4X1D	ICD10	Epidural hemorrhage with loss of consciousness of 30 minutes or less, subsequent encounter
S06.4X1S	ICD10	Epidural hemorrhage with loss of consciousness of 30 minutes or less, sequela
S06.4X2	ICD10	Epidural hemorrhage w/LOC of 31-59 minutes
S06.4X2A	ICD10	Epidural hemorrhage with loss of consciousness of 31 minutes to 59 minutes, initial encounter
S06.4X2D	ICD10	Epidural hemorrhage with loss of consciousness of 31 minutes to 59 minutes, subsequent encounter
S06.4X2S	ICD10	Epidural hemorrhage with loss of consciousness of 31 minutes to 59 minutes, sequela
S06.4X3	ICD10	Epidural hemorrhage w/LOC of 1 hr to 5 hrs 59 mins

© 2023 Brenner LA et al. *JAMA Network Open.*

S06.4X3A	ICD10	Epidural hemorrhage with loss of consciousness of 1 hour to 5 hours 59 minutes, initial encounter
S06.4X3D	ICD10	Epidural hemorrhage with loss of consciousness of 1 hour to 5 hours 59 minutes, subsequent encounter
S06.4X3S	ICD10	Epidural hemorrhage with loss of consciousness of 1 hour to 5 hours 59 minutes, sequela
S06.4X4	ICD10	Epidural hemorrhage w/LOC of 6-24 hrs
S06.4X4A	ICD10	Epidural hemorrhage with loss of consciousness of 6 hours to 24 hours, initial encounter
S06.4X4D	ICD10	Epidural hemorrhage with loss of consciousness of 6 hours to 24 hours, subsequent encounter
S06.4X4S	ICD10	Epidural hemorrhage with loss of consciousness of 6 hours to 24 hours, sequela
S06.4X5	ICD10	Epidural hemorrhage w/LOC >24 hrs with return to pre-existing conscious level
S06.4X5A	ICD10	Epidural hemorrhage with loss of consciousness greater than 24 hours with return to pre-existing conscious level, initial encounter
S06.4X5D	ICD10	Epidural hemorrhage with loss of consciousness greater than 24 hours with return to pre-existing conscious level, subsequent encounter
S06.4X5S	ICD10	Epidural hemorrhage with loss of consciousness greater than 24 hours with return to pre-existing conscious level, sequela
S06.4X6	ICD10	Epidural hemorrhage w/LOC >24 hrs w/o return to pre-existing conscious level with patient surviving
S06.4X6A	ICD10	Epidural hemorrhage with loss of consciousness greater than 24 hours without return to pre-existing conscious level with patient surviving, initial encounter
S06.4X6D	ICD10	Epidural hemorrhage with loss of consciousness greater than 24 hours without return to pre-existing conscious level with patient surviving, subsequent encounter
S06.4X6S	ICD10	Epidural hemorrhage with loss of consciousness greater than 24 hours without return to pre-existing conscious level with patient surviving, sequela
S06.4X7	ICD10	Epidural hemorrhage w/LOC of any duration with death due to brain injury prior to regaining consciousness
S06.4X7A	ICD10	Epidural hemorrhage with loss of consciousness of any duration with death due to brain injury prior to regaining consciousness, initial encounter
S06.4X7D	ICD10	Epidural hemorrhage with loss of consciousness of any duration with death due to brain injury prior to regaining consciousness, subsequent encounter
S06.4X7S	ICD10	Epidural hemorrhage with loss of consciousness of any duration with death due to brain injury prior to regaining consciousness, sequela
S06.4X8	ICD10	Epidural hemorrhage w/LOC of any duration with death due to other causes prior to regaining consciousness
S06.4X8A	ICD10	Epidural hemorrhage with loss of consciousness of any duration with death due to other causes prior to regaining consciousness, initial encounter
S06.4X8D	ICD10	Epidural hemorrhage with loss of consciousness of any duration with death due to other causes prior to regaining consciousness, subsequent encounter
S06.4X8S	ICD10	Epidural hemorrhage with loss of consciousness of any duration with death due to other causes prior to regaining consciousness, sequela
S06.4X9	ICD10	Epidural hemorrhage w/LOC of unspecified duration
S06.4X9A	ICD10	Epidural hemorrhage with loss of consciousness of unspecified duration, initial encounter
S06.4X9D	ICD10	Epidural hemorrhage with loss of consciousness of unspecified duration, subsequent encounter

© 2023 Brenner LA et al. *JAMA Network Open.*

S06.4X9S	ICD10	Epidural hemorrhage with loss of consciousness of unspecified duration, sequela
S06.5X	ICD10	Traumatic subdural hemorrhage
S06.5X0	ICD10	Traumatic subdural hemorrhage w/o LOC
S06.5X0A	ICD10	Traumatic subdural hemorrhage without loss of consciousness, initial encounter
S06.5X0D	ICD10	Traumatic subdural hemorrhage without loss of consciousness, subsequent encounter
S06.5X0S	ICD10	Traumatic subdural hemorrhage without loss of consciousness, sequela
S06.5X1	ICD10	Traumatic subdural hemorrhage w/LOC of 30 mins or less
S06.5X1A	ICD10	Traumatic subdural hemorrhage with loss of consciousness of 30 minutes or less, initial encounter
S06.5X1D	ICD10	Traumatic subdural hemorrhage with loss of consciousness of 30 minutes or less, subsequent encounter
S06.5X1S	ICD10	Traumatic subdural hemorrhage with loss of consciousness of 30 minutes or less, sequela
S06.5X2	ICD10	Traumatic subdural hemorrhage with LOC of 31-59 mins
S06.5X2A	ICD10	Traumatic subdural hemorrhage with loss of consciousness of 31 minutes to 59 minutes, initial encounter
S06.5X2D	ICD10	Traumatic subdural hemorrhage with loss of consciousness of 31 minutes to 59 minutes, subsequent encounter
S06.5X2S	ICD10	Traumatic subdural hemorrhage with loss of consciousness of 31 minutes to 59 minutes, sequela
S06.5X3	ICD10	Traumatic subdural hemorrhage w/LOC of 1 hr to 5 hrs 59 mins
S06.5X3A	ICD10	Traumatic subdural hemorrhage with loss of consciousness of 1 hour to 5 hours 59 minutes, initial encounter
S06.5X3D	ICD10	Traumatic subdural hemorrhage with loss of consciousness of 1 hour to 5 hours 59 minutes, subsequent encounter
S06.5X3S	ICD10	Traumatic subdural hemorrhage with loss of consciousness of 1 hour to 5 hours 59 minutes, sequela
S06.5X4	ICD10	Traumatic subdural hemorrhage with LOC of 6-24 hrs
S06.5X4A	ICD10	Traumatic subdural hemorrhage with loss of consciousness of 6 hours to 24 hours, initial encounter
S06.5X4D	ICD10	Traumatic subdural hemorrhage with loss of consciousness of 6 hours to 24 hours, subsequent encounter
S06.5X4S	ICD10	Traumatic subdural hemorrhage with loss of consciousness of 6 hours to 24 hours, sequela
S06.5X5	ICD10	Traumatic subdural hemorrhage w/LOC >24 hrs with return to pre-existing conscious level
S06.5X5A	ICD10	Traumatic subdural hemorrhage with loss of consciousness greater than 24 hours with return to pre-existing conscious level, initial encounter
S06.5X5D	ICD10	Traumatic subdural hemorrhage with loss of consciousness greater than 24 hours with return to pre-existing conscious level, subsequent encounter
S06.5X5S	ICD10	Traumatic subdural hemorrhage with loss of consciousness greater than 24 hours with return to pre-existing conscious level, sequela
S06.5X6	ICD10	Traumatic subdural hemorrhage w/ LOC >24 hrs w/o return to pre-existing conscious level with patient surviving
S06.5X6A	ICD10	Traumatic subdural hemorrhage with loss of consciousness greater than 24 hours without return to pre-existing conscious level with patient surviving, initial encounter
S06.5X6D	ICD10	Traumatic subdural hemorrhage with loss of consciousness greater than 24 hours without return to pre-existing conscious level with patient surviving, subsequent encounter

© 2023 Brenner LA et al. *JAMA Network Open.*

S06.5X6S	ICD10	Traumatic subdural hemorrhage with loss of consciousness greater than 24 hours without return to pre-existing conscious level with patient surviving, sequela
S06.5X7	ICD10	Traumatic subdural hemorrhage w/LOC of any duration with death due to brain injury before regaining consciousness
S06.5X7A	ICD10	Traumatic subdural hemorrhage with loss of consciousness of any duration with death due to brain injury before regaining consciousness, initial encounter
S06.5X7D	ICD10	Traumatic subdural hemorrhage with loss of consciousness of any duration with death due to brain injury before regaining consciousness, subsequent encounter
S06.5X7S	ICD10	Traumatic subdural hemorrhage with loss of consciousness of any duration with death due to brain injury before regaining consciousness, sequela
S06.5X8	ICD10	Traumatic subdural hemorrhage w/LOC of any duration with death due to other causes before regaining consciousness
S06.5X8A	ICD10	Traumatic subdural hemorrhage with loss of consciousness of any duration with death due to other cause before regaining consciousness, initial encounter
S06.5X8D	ICD10	Traumatic subdural hemorrhage with loss of consciousness of any duration with death due to other cause before regaining consciousness, subsequent encounter
S06.5X8S	ICD10	Traumatic subdural hemorrhage with loss of consciousness of any duration with death due to other cause before regaining consciousness, sequela
S06.5X9	ICD10	Traumatic subdural hemorrhage w/LOC of unspecified duration
S06.5X9A	ICD10	Traumatic subdural hemorrhage with loss of consciousness of unspecified duration, initial encounter
S06.5X9D	ICD10	Traumatic subdural hemorrhage with loss of consciousness of unspecified duration, subsequent encounter
S06.5X9S	ICD10	Traumatic subdural hemorrhage with loss of consciousness of unspecified duration, sequela
S06.6X	ICD10	Traumatic subarachnoid hemorrhage
S06.6X0	ICD10	Traumatic subarachnoid hemorrhage w/o LOC
S06.6X0A	ICD10	Traumatic subarachnoid hemorrhage without loss of consciousness, initial encounter
S06.6X0D	ICD10	Traumatic subarachnoid hemorrhage without loss of consciousness, subsequent encounter
S06.6X0S	ICD10	Traumatic subarachnoid hemorrhage without loss of consciousness, sequela
S06.6X1	ICD10	Traumatic subarachnoid hemorrhage w/LOC of 30 mins or less
S06.6X1A	ICD10	Traumatic subarachnoid hemorrhage with loss of consciousness of 30 minutes or less, initial encounter
S06.6X1D	ICD10	Traumatic subarachnoid hemorrhage with loss of consciousness of 30 minutes or less, subsequent encounter
S06.6X1S	ICD10	Traumatic subarachnoid hemorrhage with loss of consciousness of 30 minutes or less, sequela
S06.6X2	ICD10	Traumatic subarachnoid hemorrhage w/LOC of 31-59 mins
S06.6X2A	ICD10	Traumatic subarachnoid hemorrhage with loss of consciousness of 31 minutes to 59 minutes, initial encounter
S06.6X2D	ICD10	Traumatic subarachnoid hemorrhage with loss of consciousness of 31 minutes to 59 minutes, subsequent encounter
S06.6X2S	ICD10	Traumatic subarachnoid hemorrhage with loss of consciousness of 31 minutes to 59 minutes, sequela
S06.6X3	ICD10	Traumatic subarachnoid hemorrhage w/ LOC of 1 hr to 5 hrs 59 mins

© 2023 Brenner LA et al. *JAMA Network Open.*

S06.6X3A	ICD10	Traumatic subarachnoid hemorrhage with loss of consciousness of 1 hour to 5 hours 59 minutes, initial encounter
S06.6X3D	ICD10	Traumatic subarachnoid hemorrhage with loss of consciousness of 1 hour to 5 hours 59 minutes, subsequent encounter
S06.6X3S	ICD10	Traumatic subarachnoid hemorrhage with loss of consciousness of 1 hour to 5 hours 59 minutes, sequela
S06.6X4	ICD10	Traumatic subarachnoid hemorrhage w/LOC of 6-24 hrs
S06.6X4A	ICD10	Traumatic subarachnoid hemorrhage with loss of consciousness of 6 hours to 24 hours, initial encounter
S06.6X4D	ICD10	Traumatic subarachnoid hemorrhage with loss of consciousness of 6 hours to 24 hours, subsequent encounter
S06.6X4S	ICD10	Traumatic subarachnoid hemorrhage with loss of consciousness of 6 hours to 24 hours, sequela
S06.6X5	ICD10	Traumatic subarachnoid hemorrhage w/LOC > 24hrs with return to pre-existing conscious level
S06.6X5A	ICD10	Traumatic subarachnoid hemorrhage with loss of consciousness greater than 24 hours with return to pre-existing conscious level, initial encounter
S06.6X5D	ICD10	Traumatic subarachnoid hemorrhage with loss of consciousness greater than 24 hours with return to pre-existing conscious level, subsequent encounter
S06.6X5S	ICD10	Traumatic subarachnoid hemorrhage with loss of consciousness greater than 24 hours with return to pre-existing conscious level, sequela
S06.6X6	ICD10	Traumatic subarachnoid hemorrhage w/LOC > 24 hrs w/o return to pre-existing conscious level with patient surviving
S06.6X6A	ICD10	Traumatic subarachnoid hemorrhage with loss of consciousness greater than 24 hours without return to pre-existing conscious level with patient surviving, initial encounter
S06.6X6D	ICD10	Traumatic subarachnoid hemorrhage with loss of consciousness greater than 24 hours without return to pre-existing conscious level with patient surviving, subsequent encounter
S06.6X6S	ICD10	Traumatic subarachnoid hemorrhage with loss of consciousness greater than 24 hours without return to pre-existing conscious level with patient surviving, sequela
S06.6X7	ICD10	Traumatic subarachnoid hemorrhage w/LOC of any duration with death due to brain injury prior to regaining consciousness
S06.6X7A	ICD10	Traumatic subarachnoid hemorrhage with loss of consciousness of any duration with death due to brain injury prior to regaining consciousness, initial encounter
S06.6X7D	ICD10	Traumatic subarachnoid hemorrhage with loss of consciousness of any duration with death due to brain injury prior to regaining consciousness, subsequent encounter
S06.6X7S	ICD10	Traumatic subarachnoid hemorrhage with loss of consciousness of any duration with death due to brain injury prior to regaining consciousness, sequela
S06.6X8	ICD10	Traumatic subarachnoid hemorrhage w/LOC of any duration with death due to other causes before regaining consciousness
S06.6X8A	ICD10	Traumatic subarachnoid hemorrhage with loss of consciousness of any duration with death due to other cause prior to regaining consciousness, initial encounter
S06.6X8D	ICD10	Traumatic subarachnoid hemorrhage with loss of consciousness of any duration with death due to other cause prior to regaining consciousness, subsequent encounter

© 2023 Brenner LA et al. *JAMA Network Open.*

S06.6X8S	ICD10	Traumatic subarachnoid hemorrhage with loss of consciousness of any duration with death due to other cause prior to regaining consciousness, sequela
S06.6X9	ICD10	Traumatic subarachnoid hemorrhage w/LOC of unspecified duration
S06.6X9A	ICD10	Traumatic subarachnoid hemorrhage with loss of consciousness of unspecified duration, initial encounter
S06.6X9D	ICD10	Traumatic subarachnoid hemorrhage with loss of consciousness of unspecified duration, subsequent encounter
S06.6X9S	ICD10	Traumatic subarachnoid hemorrhage with loss of consciousness of unspecified duration, sequela
S06.810A	ICD10	Injury of right internal carotid artery, intracranial portion, not elsewhere classified without loss of consciousness, initial encounter
S06.810D	ICD10	Injury of right internal carotid artery, intracranial portion, not elsewhere classified without loss of consciousness, subsequent encounter
S06.810S	ICD10	Injury of right internal carotid artery, intracranial portion, not elsewhere classified without loss of consciousness, sequela
S06.811A	ICD10	Injury of right internal carotid artery, intracranial portion, not elsewhere classified with loss of consciousness of 30 minutes or less, initial encounter
S06.811D	ICD10	Injury of right internal carotid artery, intracranial portion, not elsewhere classified with loss of consciousness of 30 minutes or less, subsequent encounter
S06.811S	ICD10	Injury of right internal carotid artery, intracranial portion, not elsewhere classified with loss of consciousness of 30 minutes or less, sequela
S06.812A	ICD10	Injury of right internal carotid artery, intracranial portion, not elsewhere classified with loss of consciousness of 31 minutes to 59 minutes, initial encounter
S06.812D	ICD10	Injury of right internal carotid artery, intracranial portion, not elsewhere classified with loss of consciousness of 31 minutes to 59 minutes, subsequent encounter
S06.812S	ICD10	Injury of right internal carotid artery, intracranial portion, not elsewhere classified with loss of consciousness of 31 minutes to 59 minutes, sequela
S06.813A	ICD10	Injury of right internal carotid artery, intracranial portion, not elsewhere classified with loss of consciousness of 1 hour to 5 hours 59 minutes, initial encounter
S06.813D	ICD10	Injury of right internal carotid artery, intracranial portion, not elsewhere classified with loss of consciousness of 1 hour to 5 hours 59 minutes, subsequent encounter
S06.813S	ICD10	Injury of right internal carotid artery, intracranial portion, not elsewhere classified with loss of consciousness of 1 hour to 5 hours 59 minutes, sequela
S06.814A	ICD10	Injury of right internal carotid artery, intracranial portion, not elsewhere classified with loss of consciousness of 6 hours to 24 hours, initial encounter
S06.814D	ICD10	Injury of right internal carotid artery, intracranial portion, not elsewhere classified with loss of consciousness of 6 hours to 24 hours, subsequent encounter
S06.814S	ICD10	Injury of right internal carotid artery, intracranial portion, not elsewhere classified with loss of consciousness of 6 hours to 24 hours, sequela
S06.815A	ICD10	Injury of right internal carotid artery, intracranial portion, not elsewhere classified with loss of consciousness greater than 24 hours with return to pre-existing conscious level, initial encounter

© 2023 Brenner LA et al. *JAMA Network Open.*

S06.815D	ICD10	Injury of right internal carotid artery, intracranial portion, not elsewhere classified with loss of consciousness greater than 24 hours with return to pre-existing conscious level, subsequent encounter
S06.815S	ICD10	Injury of right internal carotid artery, intracranial portion, not elsewhere classified with loss of consciousness greater than 24 hours with return to pre-existing conscious level, sequela
S06.816A	ICD10	Injury of right internal carotid artery, intracranial portion, not elsewhere classified with loss of consciousness greater than 24 hours without return to pre-existing conscious level with patient surviving, initial encounter
S06.816D	ICD10	Injury of right internal carotid artery, intracranial portion, not elsewhere classified with loss of consciousness greater than 24 hours without return to pre-existing conscious level with patient surviving, subsequent encounter
S06.816S	ICD10	Injury of right internal carotid artery, intracranial portion, not elsewhere classified with loss of consciousness greater than 24 hours without return to pre-existing conscious level with patient surviving, sequela
S06.817A	ICD10	Injury of right internal carotid artery, intracranial portion, not elsewhere classified with loss of consciousness of any duration with death due to brain injury prior to regaining consciousness, initial encounter
S06.817D	ICD10	Injury of right internal carotid artery, intracranial portion, not elsewhere classified with loss of consciousness of any duration with death due to brain injury prior to regaining consciousness, subsequent encounter
S06.817S	ICD10	Injury of right internal carotid artery, intracranial portion, not elsewhere classified with loss of consciousness of any duration with death due to brain injury prior to regaining consciousness, sequela
S06.818A	ICD10	Injury of right internal carotid artery, intracranial portion, not elsewhere classified with loss of consciousness of any duration with death due to other cause prior to regaining consciousness, initial encounter
S06.818D	ICD10	Injury of right internal carotid artery, intracranial portion, not elsewhere classified with loss of consciousness of any duration with death due to other cause prior to regaining consciousness, subsequent encounter
S06.818S	ICD10	Injury of right internal carotid artery, intracranial portion, not elsewhere classified with loss of consciousness of any duration with death due to other cause prior to regaining consciousness, sequela
S06.819A	ICD10	Injury of right internal carotid artery, intracranial portion, not elsewhere classified with loss of consciousness of unspecified duration, initial encounter
S06.819D	ICD10	Injury of right internal carotid artery, intracranial portion, not elsewhere classified with loss of consciousness of unspecified duration, subsequent encounter
S06.819S	ICD10	Injury of right internal carotid artery, intracranial portion, not elsewhere classified with loss of consciousness of unspecified duration, sequela
S06.820A	ICD10	Injury of left internal carotid artery, intracranial portion, not elsewhere classified without loss of consciousness, initial encounter
S06.820D	ICD10	Injury of left internal carotid artery, intracranial portion, not elsewhere classified without loss of consciousness, subsequent encounter
S06.820S	ICD10	Injury of left internal carotid artery, intracranial portion, not elsewhere classified without loss of consciousness, sequela
S06.821A	ICD10	Injury of left internal carotid artery, intracranial portion, not elsewhere classified with loss of consciousness of 30 minutes or less, initial encounter
S06.821D	ICD10	Injury of left internal carotid artery, intracranial portion, not elsewhere classified with loss of consciousness of 30 minutes or less, subsequent encounter

© 2023 Brenner LA et al. *JAMA Network Open.*

S06.821S	ICD10	Injury of left internal carotid artery, intracranial portion, not elsewhere classified with loss of consciousness of 30 minutes or less, sequela
S06.822A	ICD10	Injury of left internal carotid artery, intracranial portion, not elsewhere classified with loss of consciousness of 31 minutes to 59 minutes, initial encounter
S06.822D	ICD10	Injury of left internal carotid artery, intracranial portion, not elsewhere classified with loss of consciousness of 31 minutes to 59 minutes, subsequent encounter
S06.822S	ICD10	Injury of left internal carotid artery, intracranial portion, not elsewhere classified with loss of consciousness of 31 minutes to 59 minutes, sequela
S06.823A	ICD10	Injury of left internal carotid artery, intracranial portion, not elsewhere classified with loss of consciousness of 1 hour to 5 hours 59 minutes, initial encounter
S06.823D	ICD10	Injury of left internal carotid artery, intracranial portion, not elsewhere classified with loss of consciousness of 1 hour to 5 hours 59 minutes, subsequent encounter
S06.823S	ICD10	Injury of left internal carotid artery, intracranial portion, not elsewhere classified with loss of consciousness of 1 hour to 5 hours 59 minutes, sequela
S06.824A	ICD10	Injury of left internal carotid artery, intracranial portion, not elsewhere classified with loss of consciousness of 6 hours to 24 hours, initial encounter
S06.824D	ICD10	Injury of left internal carotid artery, intracranial portion, not elsewhere classified with loss of consciousness of 6 hours to 24 hours, subsequent encounter
S06.824S	ICD10	Injury of left internal carotid artery, intracranial portion, not elsewhere classified with loss of consciousness of 6 hours to 24 hours, sequela
S06.825A	ICD10	Injury of left internal carotid artery, intracranial portion, not elsewhere classified with loss of consciousness greater than 24 hours with return to pre-existing conscious level, initial encounter
S06.825D	ICD10	Injury of left internal carotid artery, intracranial portion, not elsewhere classified with loss of consciousness greater than 24 hours with return to pre-existing conscious level, subsequent encounter
S06.825S	ICD10	Injury of left internal carotid artery, intracranial portion, not elsewhere classified with loss of consciousness greater than 24 hours with return to pre-existing conscious level, sequela
S06.826A	ICD10	Injury of left internal carotid artery, intracranial portion, not elsewhere classified with loss of consciousness greater than 24 hours without return to pre-existing conscious level with patient surviving, initial encounter
S06.826D	ICD10	Injury of left internal carotid artery, intracranial portion, not elsewhere classified with loss of consciousness greater than 24 hours without return to pre-existing conscious level with patient surviving, subsequent encounter
S06.826S	ICD10	Injury of left internal carotid artery, intracranial portion, not elsewhere classified with loss of consciousness greater than 24 hours without return to pre-existing conscious level with patient surviving, sequela
S06.827A	ICD10	Injury of left internal carotid artery, intracranial portion, not elsewhere classified with loss of consciousness of any duration with death due to brain injury prior to regaining consciousness, initial encounter
S06.827D	ICD10	Injury of left internal carotid artery, intracranial portion, not elsewhere classified with loss of consciousness of any duration with death due to brain injury prior to regaining consciousness, subsequent encounter
S06.827S	ICD10	Injury of left internal carotid artery, intracranial portion, not elsewhere classified with loss of consciousness of any duration with death due to brain injury prior to regaining consciousness, sequela

© 2023 Brenner LA et al. *JAMA Network Open.*

S06.828A	ICD10	Injury of left internal carotid artery, intracranial portion, not elsewhere classified with loss of consciousness of any duration with death due to other cause prior to regaining consciousness, initial encounter
S06.828D	ICD10	Injury of left internal carotid artery, intracranial portion, not elsewhere classified with loss of consciousness of any duration with death due to other cause prior to regaining consciousness, subsequent encounter
S06.828S	ICD10	Injury of left internal carotid artery, intracranial portion, not elsewhere classified with loss of consciousness of any duration with death due to other cause prior to regaining consciousness, sequela
S06.829A	ICD10	Injury of left internal carotid artery, intracranial portion, not elsewhere classified with loss of consciousness of unspecified duration, initial encounter
S06.829D	ICD10	Injury of left internal carotid artery, intracranial portion, not elsewhere classified with loss of consciousness of unspecified duration, subsequent encounter
S06.829S	ICD10	Injury of left internal carotid artery, intracranial portion, not elsewhere classified with loss of consciousness of unspecified duration, sequela
S06.89	ICD10	Other specified intracranial injury
S06.890	ICD10	Other specified intracranial injury w/o LOC
S06.890A	ICD10	Other specified intracranial injury without loss of consciousness, initial encounter
S06.890D	ICD10	Other specified intracranial injury without loss of consciousness, subsequent encounter
S06.890S	ICD10	Other specified intracranial injury without loss of consciousness, sequela
S06.891	ICD10	Other specified intracranial injury w/LOC of 30 mins or less
S06.891A	ICD10	Other specified intracranial injury with loss of consciousness of 30 minutes or less, initial encounter
S06.891D	ICD10	Other specified intracranial injury with loss of consciousness of 30 minutes or less, subsequent encounter
S06.891S	ICD10	Other specified intracranial injury with loss of consciousness of 30 minutes or less, sequela
S06.892	ICD10	Other specified intracranial injury w/LOC of 31-59 mins
S06.892A	ICD10	Other specified intracranial injury with loss of consciousness of 31 minutes to 59 minutes, initial encounter
S06.892D	ICD10	Other specified intracranial injury with loss of consciousness of 31 minutes to 59 minutes, subsequent encounter
S06.892S	ICD10	Other specified intracranial injury with loss of consciousness of 31 minutes to 59 minutes, sequela
S06.893	ICD10	Other specified intracranial injury w/LOC of 1 hr to 5 hrs 59 mins
S06.893A	ICD10	Other specified intracranial injury with loss of consciousness of 1 hour to 5 hours 59 minutes, initial encounter
S06.893D	ICD10	Other specified intracranial injury with loss of consciousness of 1 hour to 5 hours 59 minutes, subsequent encounter
S06.893S	ICD10	Other specified intracranial injury with loss of consciousness of 1 hour to 5 hours 59 minutes, sequela
S06.894	ICD10	Other specified intracranial injury w/LOC of 6 -24 hrs
S06.894A	ICD10	Other specified intracranial injury with loss of consciousness of 6 hours to 24 hours, initial encounter
S06.894D	ICD10	Other specified intracranial injury with loss of consciousness of 6 hours to 24 hours, subsequent encounter
S06.894S	ICD10	Other specified intracranial injury with loss of consciousness of 6 hours to 24 hours, sequela
S06.895	ICD10	Other specified intracranial injury w/LOC > 24 hrs with return to pre-existing conscious level

© 2023 Brenner LA et al. *JAMA Network Open.*

S06.895A	ICD10	Other specified intracranial injury with loss of consciousness greater than 24 hours with return to pre-existing conscious level, initial encounter
S06.895D	ICD10	Other specified intracranial injury with loss of consciousness greater than 24 hours with return to pre-existing conscious level, subsequent encounter
S06.895S	ICD10	Other specified intracranial injury with loss of consciousness greater than 24 hours with return to pre-existing conscious level, sequela
S06.896	ICD10	Other specified intracranial injury w/LOC >24 hrs w/o return to pre-existing conscious level with patient surviving
S06.896A	ICD10	Other specified intracranial injury with loss of consciousness greater than 24 hours without return to pre-existing conscious level with patient surviving, initial encounter
S06.896D	ICD10	Other specified intracranial injury with loss of consciousness greater than 24 hours without return to pre-existing conscious level with patient surviving, subsequent encounter
S06.896S	ICD10	Other specified intracranial injury with loss of consciousness greater than 24 hours without return to pre-existing conscious level with patient surviving, sequela
S06.897	ICD10	Other specified intracranial injury w/LOC of any duration with death due to brain injury prior to regaining consciousness
S06.897A	ICD10	Other specified intracranial injury with loss of consciousness of any duration with death due to brain injury prior to regaining consciousness, initial encounter
S06.897D	ICD10	Other specified intracranial injury with loss of consciousness of any duration with death due to brain injury prior to regaining consciousness, subsequent encounter
S06.897S	ICD10	Other specified intracranial injury with loss of consciousness of any duration with death due to brain injury prior to regaining consciousness, subsequent encounter
S06.898	ICD10	Other specified intracranial injury w/LOC of any duration with death due to other cause prior to regaining consciousness
S06.898A	ICD10	Other specified intracranial injury with loss of consciousness of any duration with death due to other cause prior to regaining consciousness, initial encounter
S06.898D	ICD10	Other specified intracranial injury with loss of consciousness of any duration with death due to other cause prior to regaining consciousness, subsequent encounter
S06.898S	ICD10	Other specified intracranial injury with loss of consciousness of any duration with death due to other cause prior to regaining consciousness, sequela
S06.899	ICD10	Other specified intracranial injury w/LOC of unspecified duration
S06.899A	ICD10	Other specified intracranial injury with loss of consciousness of unspecified duration, initial encounter
S06.899D	ICD10	Other specified intracranial injury with loss of consciousness of unspecified duration, subsequent encounter
S06.899S	ICD10	Other specified intracranial injury with loss of consciousness of unspecified duration, sequela
S06.9X	ICD10	Unspecified intracranial injury
S06.9X0	ICD10	Unspecified intracranial injury w/o LOC
S06.9X0A	ICD10	Unspecified intracranial injury without loss of consciousness, initial encounter
S06.9X0D	ICD10	Unspecified intracranial injury without loss of consciousness, subsequent encounter

© 2023 Brenner LA et al. *JAMA Network Open.*

S06.9X0S	ICD10	Unspecified intracranial injury without loss of consciousness, sequela
S06.9X1	ICD10	Unspecified intracranial injury w/LOC of 30 mins or less
S06.9X1A	ICD10	Unspecified intracranial injury with loss of consciousness of 30 minutes or less, initial encounter
S06.9X1D	ICD10	Unspecified intracranial injury with loss of consciousness of 30 minutes or less, subsequent encounter
S06.9X1S	ICD10	Unspecified intracranial injury with loss of consciousness of 30 minutes or less, sequela
S06.9X2	ICD10	Unspecified intracranial injury w/LOC of 31-59 mins
S06.9X2A	ICD10	Unspecified intracranial injury with loss of consciousness of 31 minutes to 59 minutes, initial encounter
S06.9X2D	ICD10	Unspecified intracranial injury with loss of consciousness of 31 minutes to 59 minutes, subsequent encounter
S06.9X2S	ICD10	Unspecified intracranial injury with loss of consciousness of 31 minutes to 59 minutes, sequela
S06.9X3	ICD10	Unspecified intracranial injury w/LOC of 1 hr to 5 hrs 59 min
S06.9X3A	ICD10	Unspecified intracranial injury with loss of consciousness of 1 hour to 5 hours 59 minutes, initial encounter
S06.9X3D	ICD10	Unspecified intracranial injury with loss of consciousness of 1 hour to 5 hours 59 minutes, subsequent encounter
S06.9X3S	ICD10	Unspecified intracranial injury with loss of consciousness of 1 hour to 5 hours 59 minutes, sequela
S06.9X4	ICD10	Unspecified intracranial injury w/ LOC of 6 -24 hrs
S06.9X4A	ICD10	Unspecified intracranial injury with loss of consciousness of 6 hours to 24 hours, initial encounter
S06.9X4D	ICD10	Unspecified intracranial injury with loss of consciousness of 6 hours to 24 hours, subsequent encounter
S06.9X4S	ICD10	Unspecified intracranial injury with loss of consciousness of 6 hours to 24 hours, sequela
S06.9X5	ICD10	Unspecified intracranial injury w/LOC > 24 hrs with return to pre-existing conscious level
S06.9X5A	ICD10	Unspecified intracranial injury with loss of consciousness greater than 24 hours with return to pre-existing conscious level, initial encounter
S06.9X5D	ICD10	Unspecified intracranial injury with loss of consciousness greater than 24 hours with return to pre-existing conscious level, subsequent encounter
S06.9X5S	ICD10	Unspecified intracranial injury with loss of consciousness greater than 24 hours with return to pre-existing conscious level, sequela
S06.9X6	ICD10	Unspecified intracranial injury w/LOC >24 hrs w/o return to pre-existing conscious level with patient surviving
S06.9X6A	ICD10	Unspecified intracranial injury with loss of consciousness greater than 24 hours without return to pre-existing conscious level with patient surviving, initial encounter
S06.9X6D	ICD10	Unspecified intracranial injury with loss of consciousness greater than 24 hours without return to pre-existing conscious level with patient surviving, subsequent encounter
S06.9X6S	ICD10	Unspecified intracranial injury with loss of consciousness greater than 24 hours without return to pre-existing conscious level with patient surviving, sequela
S06.9X7	ICD10	Unspecified intracranial injury w/LOC of any duration with death due to brain injury prior to regaining consciousness
S06.9X7A	ICD10	Unspecified intracranial injury with loss of consciousness of any duration with death due to brain injury prior to regaining consciousness, initial encounter

© 2023 Brenner LA et al. *JAMA Network Open.*

S06.9X7D	ICD10	Unspecified intracranial injury with loss of consciousness of any duration with death due to brain injury prior to regaining consciousness, subsequent encounter
S06.9X7S	ICD10	Unspecified intracranial injury with loss of consciousness of any duration with death due to brain injury prior to regaining consciousness, sequela
S06.9X8	ICD10	Unspecified intracranial injury w/LOC of any duration with death due to other cause prior to regaining consciousness
S06.9X8A	ICD10	Unspecified intracranial injury with loss of consciousness of any duration with death due to other cause prior to regaining consciousness, initial encounter
S06.9X8D	ICD10	Unspecified intracranial injury with loss of consciousness of any duration with death due to other cause prior to regaining consciousness, subsequent encounter
S06.9X8S	ICD10	Unspecified intracranial injury with loss of consciousness of any duration with death due to other cause prior to regaining consciousness, sequela
S06.9X9	ICD10	Unspecified intracranial injury w/LOC of unspecified duration
S06.9X9A	ICD10	Unspecified intracranial injury with loss of consciousness of unspecified duration, initial encounter
S06.9X9D	ICD10	Unspecified intracranial injury with loss of consciousness of unspecified duration, subsequent encounter
S06.9X9S	ICD10	Unspecified intracranial injury with loss of consciousness of unspecified duration, sequela
Z87.820	ICD10	Personal history of traumatic brain injury

© 2023 Brenner LA et al. *JAMA Network Open.*

**eTable 2:** Mental Health *ICD-9* and *ICD-10* Codes and Classifications			
**ICD Code**	**Code Type**	**Classification**	**Description**
309.0	ICD-9	Adjustment	Adjustment disorder with depressed mood
309.1	ICD-9	Adjustment	Prolonged depressive reaction
309.21	ICD-9	Adjustment	Separation anxiety disorder
309.22	ICD-9	Adjustment	Emancipation disorder of adolescence and early adult life
309.23	ICD-9	Adjustment	Specific academic or work inhibition
309.24	ICD-9	Adjustment	Adjustment disorder with anxiety
309.28	ICD-9	Adjustment	Adjustment disorder with mixed anxiety and depressed mood
309.29	ICD-9	Adjustment	Other adjustment reactions with predominant disturbance of other emotions
309.3	ICD-9	Adjustment	Adjustment disorder with disturbance of conduct
309.4	ICD-9	Adjustment	Adjustment disorder with mixed disturbance of emotions and conduct
309.82	ICD-9	Adjustment	Adjustment reaction with physical symptoms
309.83	ICD-9	Adjustment	Adjustment reaction with withdrawal
309.89	ICD-9	Adjustment	Other specified adjustment reactions
309.9	ICD-9	Adjustment	Unspecified adjustment reaction
F43.20	ICD-10	Adjustment	Adjustment disorder, unspecified
F43.21	ICD-10	Adjustment	Adjustment disorder with depressed mood
F43.22	ICD-10	Adjustment	Adjustment disorder with anxiety
F43.23	ICD-10	Adjustment	Adjustment disorder with mixed anxiety and depressed mood
F43.24	ICD-10	Adjustment	Adjustment disorder with disturbance of conduct
F43.25	ICD-10	Adjustment	Adjustment disorder with mixed disturbance of emotions and conduct
F43.29	ICD-10	Adjustment	Adjustment disorder with other symptoms
291.0	ICD-9	Alcohol	Alcohol withdrawal delirium
291.1	ICD-9	Alcohol	Alcohol-induced persisting amnestic disorder
291.2	ICD-9	Alcohol	Alcohol-induced persisting dementia
291.3	ICD-9	Alcohol	Alcohol-induced psychotic disorder with hallucinations
291.4	ICD-9	Alcohol	Idiosyncratic alcohol intoxication
291.5	ICD-9	Alcohol	Alcohol-induced psychotic disorder with delusions
291.81	ICD-9	Alcohol	Alcohol withdrawal
291.82	ICD-9	Alcohol	Alcohol induced sleep disorders
291.89	ICD-9	Alcohol	Other alcohol-induced mental disorders
291.9	ICD-9	Alcohol	Unspecified alcohol-induced mental disorders

© 2023 Brenner LA et al. *JAMA Network Open.*

303.00	ICD-9	Alcohol	Acute alcoholic intoxication in alcoholism, unspecified
303.01	ICD-9	Alcohol	Acute alcoholic intoxication in alcoholism, continuous
303.02	ICD-9	Alcohol	Acute alcoholic intoxication in alcoholism, episodic
303.03	ICD-9	Alcohol	Acute alcoholic intoxication in alcoholism, in remission
303.90	ICD-9	Alcohol	Other and unspecified alcohol dependence, unspecified
303.91	ICD-9	Alcohol	Other and unspecified alcohol dependence, continuous
303.92	ICD-9	Alcohol	Other and unspecified alcohol dependence, episodic
303.93	ICD-9	Alcohol	Other and unspecified alcohol dependence, in remission
305.00	ICD-9	Alcohol	Alcohol abuse, unspecified
305.01	ICD-9	Alcohol	Alcohol abuse, continuous
305.02	ICD-9	Alcohol	Alcohol abuse, episodic
305.03	ICD-9	Alcohol	Alcohol abuse, in remission
790.3	ICD-9	Alcohol	Excessive blood level of alcohol
980.0	ICD-9	Alcohol	Toxic effect of ethyl alcohol
V11.3	ICD-9	Alcohol	Personal history of alcoholism
F10.10	ICD-10	Alcohol	Alcohol abuse, uncomplicated
F10.11	ICD-10	Alcohol	Alcohol abuse, in remission
F10.120	ICD-10	Alcohol	Alcohol abuse with intoxication, uncomplicated
F10.121	ICD-10	Alcohol	Alcohol abuse with intoxication delirium
F10.129	ICD-10	Alcohol	Alcohol abuse with intoxication, unspecified
F10.130	ICD-10	Alcohol	Alcohol abuse with withdrawal, uncomplicated
F10.131	ICD-10	Alcohol	Alcohol abuse with withdrawal delirium
F10.132	ICD-10	Alcohol	Alcohol abuse with withdrawal with perceptual disturbance
F10.139	ICD-10	Alcohol	Alcohol abuse with withdrawal, unspecified
F10.14	ICD-10	Alcohol	Alcohol abuse with alcohol-induced mood disorder
F10.150	ICD-10	Alcohol	Alcohol abuse with alcohol-induced psychotic disorder with delusions
F10.151	ICD-10	Alcohol	Alcohol abuse with alcohol-induced psychotic disorder with hallucinations
F10.159	ICD-10	Alcohol	Alcohol abuse with alcohol-induced psychotic disorder, unspecified
F10.180	ICD-10	Alcohol	Alcohol abuse with alcohol-induced anxiety disorder
F10.181	ICD-10	Alcohol	Alcohol abuse with alcohol-induced sexual dysfunction
F10.182	ICD-10	Alcohol	Alcohol abuse with alcohol-induced sleep disorder
F10.188	ICD-10	Alcohol	Alcohol abuse with other alcohol-induced disorder
F10.19	ICD-10	Alcohol	Alcohol abuse with unspecified alcohol-induced disorder
F10.20	ICD-10	Alcohol	Alcohol dependence, uncomplicated

© 2023 Brenner LA et al. *JAMA Network Open.*

F10.21	ICD-10	Alcohol	Alcohol dependence, in remission
F10.220	ICD-10	Alcohol	Alcohol dependence with intoxication, uncomplicated
F10.221	ICD-10	Alcohol	Alcohol dependence with intoxication delirium
F10.229	ICD-10	Alcohol	Alcohol dependence with intoxication, unspecified
F10.230	ICD-10	Alcohol	Alcohol dependence with withdrawal, uncomplicated
F10.231	ICD-10	Alcohol	Alcohol dependence with withdrawal delirium
F10.232	ICD-10	Alcohol	Alcohol dependence with withdrawal with perceptual disturbance
F10.239	ICD-10	Alcohol	Alcohol dependence with withdrawal, unspecified
F10.24	ICD-10	Alcohol	Alcohol dependence with alcohol-induced mood disorder
F10.250	ICD-10	Alcohol	Alcohol dependence with alcohol-induced psychotic disorder with delusions
F10.251	ICD-10	Alcohol	Alcohol dependence with alcohol-induced psychotic disorder with hallucinations
F10.259	ICD-10	Alcohol	Alcohol dependence with alcohol-induced psychotic disorder, unspecified
F10.26	ICD-10	Alcohol	Alcohol dependence with alcohol-induced persisting amnestic disorder
F10.27	ICD-10	Alcohol	Alcohol dependence with alcohol-induced persisting dementia
F10.280	ICD-10	Alcohol	Alcohol dependence with alcohol-induced anxiety disorder
F10.281	ICD-10	Alcohol	Alcohol dependence with alcohol-induced sexual dysfunction
F10.282	ICD-10	Alcohol	Alcohol dependence with alcohol-induced sleep disorder
F10.288	ICD-10	Alcohol	Alcohol dependence with other alcohol-induced disorder
F10.29	ICD-10	Alcohol	Alcohol dependence with unspecified alcohol-induced disorder
F10.920	ICD-10	Alcohol	Alcohol use, unspecified with intoxication, uncomplicated
F10.921	ICD-10	Alcohol	Alcohol use, unspecified with intoxication delirium
F10.929	ICD-10	Alcohol	Alcohol use, unspecified with intoxication, unspecified
F10.930	ICD-10	Alcohol	Alcohol use, unspecified with withdrawal, uncomplicated
F10.931	ICD-10	Alcohol	Alcohol use, unspecified with withdrawal delirium
F10.932	ICD-10	Alcohol	Alcohol use, unspecified with withdrawal with perceptual disturbance
F10.939	ICD-10	Alcohol	Alcohol use, unspecified with withdrawal, unspecified
F10.94	ICD-10	Alcohol	Alcohol use, unspecified with alcohol-induced mood disorder
F10.950	ICD-10	Alcohol	Alcohol use, unspecified with alcohol-induced psychotic disorder with delusions
F10.951	ICD-10	Alcohol	Alcohol use, unspecified with alcohol-induced psychotic disorder with hallucinations
F10.959	ICD-10	Alcohol	Alcohol use, unspecified with alcohol-induced psychotic disorder, unspecified
F10.96	ICD-10	Alcohol	Alcohol use, unspecified with alcohol-induced persisting amnestic disorder
F10.97	ICD-10	Alcohol	Alcohol use, unspecified with alcohol-induced persisting dementia
F10.980	ICD-10	Alcohol	Alcohol use, unspecified with alcohol-induced anxiety disorder
F10.981	ICD-10	Alcohol	Alcohol use, unspecified with alcohol-induced sexual dysfunction

© 2023 Brenner LA et al. *JAMA Network Open.*

F10.982	ICD-10	Alcohol	Alcohol use, unspecified with alcohol-induced sleep disorder
F10.988	ICD-10	Alcohol	Alcohol use, unspecified with other alcohol-induced disorder
F10.99	ICD-10	Alcohol	Alcohol use, unspecified with unspecified alcohol-induced disorder
G62.1	ICD-10	Alcohol	Alcoholic polyneuropathy
I42.6	ICD-10	Alcohol	Alcoholic cardiomyopathy
K29.20	ICD-10	Alcohol	Alcoholic gastritis without bleeding
K29.21	ICD-10	Alcohol	Alcoholic gastritis with bleeding
K70.0	ICD-10	Alcohol	Alcoholic fatty liver
K70.10	ICD-10	Alcohol	Alcoholic hepatitis without ascites
K70.11	ICD-10	Alcohol	Alcoholic hepatitis with ascites
K70.2	ICD-10	Alcohol	Alcoholic fibrosis and sclerosis of liver
K70.30	ICD-10	Alcohol	Alcoholic cirrhosis of liver without ascites
K70.31	ICD-10	Alcohol	Alcoholic cirrhosis of liver with ascites
K70.40	ICD-10	Alcohol	Alcoholic hepatic failure without coma
K70.41	ICD-10	Alcohol	Alcoholic hepatic failure with coma
K70.9	ICD-10	Alcohol	Alcoholic liver disease, unspecified
293.83	ICD-9	Depression/Mood	Mood disorder in conditions classified elsewhere
296.20	ICD-9	Depression/Mood	Major depressive affective disorder, single episode, unspecified
296.21	ICD-9	Depression/Mood	Major depressive affective disorder, single episode, mild
296.22	ICD-9	Depression/Mood	Major depressive affective disorder, single episode, moderate
296.23	ICD-9	Depression/Mood	Major depressive affective disorder, single episode, severe, without mention of psychotic behavior
296.24	ICD-9	Depression/Mood	Major depressive affective disorder, single episode, severe, specified as with psychotic behavior
296.25	ICD-9	Depression/Mood	Major depressive affective disorder, single episode, in partial or unspecified remission
296.26	ICD-9	Depression/Mood	Major depressive affective disorder, single episode, in full remission
296.30	ICD-9	Depression/Mood	Major depressive affective disorder, recurrent episode, unspecified
296.31	ICD-9	Depression/Mood	Major depressive affective disorder, recurrent episode, mild
296.32	ICD-9	Depression/Mood	Major depressive affective disorder, recurrent episode, moderate
296.33	ICD-9	Depression/Mood	Major depressive affective disorder, recurrent episode, severe, without mention of psychotic behavior
296.34	ICD-9	Depression/Mood	Major depressive affective disorder, recurrent episode, severe, specified as with psychotic behavior
296.35	ICD-9	Depression/Mood	Major depressive affective disorder, recurrent episode, in partial or unspecified remission
296.36	ICD-9	Depression/Mood	Major depressive affective disorder, recurrent episode, in full remission
296.82	ICD-9	Depression/Mood	Atypical depressive disorder

© 2023 Brenner LA et al. *JAMA Network Open.*

296.90	ICD-9	Depression/Mood	Unspecified episodic mood disorder
296.99	ICD-9	Depression/Mood	Other specified episodic mood disorder
300.4	ICD-9	Depression/Mood	Dysthymic disorder
311	ICD-9	Depression/Mood	Depressive disorder, not elsewhere classified
V11.1	ICD-9	Depression/Mood	Personal history of affective disorders
F06.30	ICD-10	Depression/Mood	Mood disorder due to known physiological condition, unspecified
F06.31	ICD-10	Depression/Mood	Mood disorder due to known physiological condition with depressive features
F06.32	ICD-10	Depression/Mood	Mood disorder due to known physiological condition with major depressive-like episode
F06.34	ICD-10	Depression/Mood	Mood disorder due to known physiological condition with mixed features
F32.0	ICD-10	Depression/Mood	Major depressive disorder, single episode, mild
F32.1	ICD-10	Depression/Mood	Major depressive disorder, single episode, moderate
F32.2	ICD-10	Depression/Mood	Major depressive disorder, single episode, severe without psychotic features
F32.3	ICD-10	Depression/Mood	Major depressive disorder, single episode, severe with psychotic features
F32.4	ICD-10	Depression/Mood	Major depressive disorder, single episode, in partial remission
F32.5	ICD-10	Depression/Mood	Major depressive disorder, single episode, in full remission
F32.8	ICD-10	Depression/Mood	Other depressive episodes
F32.81	ICD-10	Depression/Mood	Premenstrual dysphoric disorder
F32.89	ICD-10	Depression/Mood	Other specified depressive episodes
F32.9	ICD-10	Depression/Mood	Major depressive disorder, single episode, unspecified
F32.A	ICD-10	Depression/Mood	Depression, unspecified
F33.0	ICD-10	Depression/Mood	Major depressive disorder, recurrent, mild
F33.1	ICD-10	Depression/Mood	Major depressive disorder, recurrent, moderate
F33.2	ICD-10	Depression/Mood	Major depressive disorder, recurrent severe without psychotic features
F33.3	ICD-10	Depression/Mood	Major depressive disorder, recurrent, severe with psychotic symptoms
F33.40	ICD-10	Depression/Mood	Major depressive disorder, recurrent, in remission, unspecified
F33.41	ICD-10	Depression/Mood	Major depressive disorder, recurrent, in partial remission
F33.42	ICD-10	Depression/Mood	Major depressive disorder, recurrent, in full remission
F33.8	ICD-10	Depression/Mood	Other recurrent depressive disorders
F33.9	ICD-10	Depression/Mood	Major depressive disorder, recurrent, unspecified
F34.1	ICD-10	Depression/Mood	Dysthymic disorder
F34.8	ICD-10	Depression/Mood	Other persistent mood [affective] disorders
F34.81	ICD-10	Depression/Mood	Disruptive mood dysregulation disorder
F34.89	ICD-10	Depression/Mood	Other specified persistent mood disorders
F34.9	ICD-10	Depression/Mood	Persistent mood [affective] disorder, unspecified

© 2023 Brenner LA et al. *JAMA Network Open.*

F39.	ICD-10	Depression/Mood	Unspecified mood [affective] disorder
293.84	ICD-9	Other Anxiety	Anxiety disorder in conditions classified elsewhere
300.00	ICD-9	Other Anxiety	Anxiety state, unspecified
300.01	ICD-9	Other Anxiety	Panic disorder without agoraphobia
300.02	ICD-9	Other Anxiety	Generalized anxiety disorder
300.09	ICD-9	Other Anxiety	Other anxiety states
300.20	ICD-9	Other Anxiety	Phobia, unspecified
300.21	ICD-9	Other Anxiety	Agoraphobia with panic disorder
300.22	ICD-9	Other Anxiety	Agoraphobia without mention of panic attacks
300.23	ICD-9	Other Anxiety	Social phobia
300.29	ICD-9	Other Anxiety	Other isolated or specific phobias
300.3	ICD-9	Other Anxiety	Obsessive-compulsive disorders
F06.4	ICD-10	Other Anxiety	Anxiety disorder due to known physiological condition
F40.00	ICD-10	Other Anxiety	Agoraphobia, unspecified
F40.01	ICD-10	Other Anxiety	Agoraphobia with panic disorder
F40.02	ICD-10	Other Anxiety	Agoraphobia without panic disorder
F40.10	ICD-10	Other Anxiety	Social phobia, unspecified
F40.11	ICD-10	Other Anxiety	Social phobia, generalized
F40.210	ICD-10	Other Anxiety	Arachnophobia
F40.218	ICD-10	Other Anxiety	Other animal type phobia
F40.220	ICD-10	Other Anxiety	Fear of thunderstorms
F40.228	ICD-10	Other Anxiety	Other natural environment type phobia
F40.230	ICD-10	Other Anxiety	Fear of blood
F40.231	ICD-10	Other Anxiety	Fear of injections and transfusions
F40.232	ICD-10	Other Anxiety	Fear of other medical care
F40.233	ICD-10	Other Anxiety	Fear of injury
F40.240	ICD-10	Other Anxiety	Claustrophobia
F40.241	ICD-10	Other Anxiety	Acrophobia
F40.242	ICD-10	Other Anxiety	Fear of bridges
F40.243	ICD-10	Other Anxiety	Fear of flying
F40.248	ICD-10	Other Anxiety	Other situational type phobia
F40.290	ICD-10	Other Anxiety	Androphobia
F40.291	ICD-10	Other Anxiety	Gynephobia
F40.298	ICD-10	Other Anxiety	Other specified phobia

© 2023 Brenner LA et al. *JAMA Network Open.*

F40.8	ICD-10	Other Anxiety	Other phobic anxiety disorders
F40.9	ICD-10	Other Anxiety	Phobic anxiety disorder, unspecified
F41.0	ICD-10	Other Anxiety	Panic disorder [episodic paroxysmal anxiety]
F41.1	ICD-10	Other Anxiety	Generalized anxiety disorder
F41.3	ICD-10	Other Anxiety	Other mixed anxiety disorders
F41.8	ICD-10	Other Anxiety	Other specified anxiety disorders
F41.9	ICD-10	Other Anxiety	Anxiety disorder, unspecified
F42.	ICD-10	Other Anxiety	Obsessive-compulsive disorder
F42.2	ICD-10	Other Anxiety	Mixed obsessional thoughts and acts
F42.3	ICD-10	Other Anxiety	Hoarding disorder
F42.4	ICD-10	Other Anxiety	Excoriation (skin-picking) disorder
F42.8	ICD-10	Other Anxiety	Other obsessive-compulsive disorder
F42.9	ICD-10	Other Anxiety	Obsessive-compulsive disorder, unspecified
R46.81	ICD-10	Other Anxiety	Obsessive-compulsive behavior
309.81	ICD-9	PTSD	Posttraumatic stress disorder
F43.10	ICD-10	PTSD	Post-traumatic stress disorder, unspecified
F43.11	ICD-10	PTSD	Post-traumatic stress disorder, acute
F43.12	ICD-10	PTSD	Post-traumatic stress disorder, chronic
292.0	ICD-9	Substance	Drug withdrawal
292.11	ICD-9	Substance	Drug-induced psychotic disorder with delusions
292.12	ICD-9	Substance	Drug-induced psychotic disorder with hallucinations
292.2	ICD-9	Substance	Pathological drug intoxication
292.81	ICD-9	Substance	Drug-induced delirium
292.82	ICD-9	Substance	Drug-induced persisting dementia
292.83	ICD-9	Substance	Drug-induced persisting amnestic disorder
292.84	ICD-9	Substance	Drug-induced mood disorder
292.85	ICD-9	Substance	Drug induced sleep disorders
292.89	ICD-9	Substance	Other specified drug-induced mental disorders
292.9	ICD-9	Substance	Unspecified drug-induced mental disorder
304.00	ICD-9	Substance	Opioid type dependence, unspecified
304.01	ICD-9	Substance	Opioid type dependence, continuous
304.02	ICD-9	Substance	Opioid type dependence, episodic
304.03	ICD-9	Substance	Opioid type dependence, in remission
304.10	ICD-9	Substance	Sedative, hypnotic or anxiolytic dependence, unspecified

© 2023 Brenner LA et al. *JAMA Network Open.*

304.11	ICD-9	Substance	Sedative, hypnotic or anxiolytic dependence, continuous
304.12	ICD-9	Substance	Sedative, hypnotic or anxiolytic dependence, episodic
304.13	ICD-9	Substance	Sedative, hypnotic or anxiolytic dependence, in remission
304.20	ICD-9	Substance	Cocaine dependence, unspecified
304.21	ICD-9	Substance	Cocaine dependence, continuous
304.22	ICD-9	Substance	Cocaine dependence, episodic
304.23	ICD-9	Substance	Cocaine dependence, in remission
304.30	ICD-9	Substance	Cannabis dependence, unspecified
304.31	ICD-9	Substance	Cannabis dependence, continuous
304.32	ICD-9	Substance	Cannabis dependence, episodic
304.33	ICD-9	Substance	Cannabis dependence, in remission
304.40	ICD-9	Substance	Amphetamine and other psychostimulant dependence, unspecified
304.41	ICD-9	Substance	Amphetamine and other psychostimulant dependence, continuous
304.42	ICD-9	Substance	Amphetamine and other psychostimulant dependence, episodic
304.43	ICD-9	Substance	Amphetamine and other psychostimulant dependence, in remission
304.50	ICD-9	Substance	Hallucinogen dependence, unspecified
304.51	ICD-9	Substance	Hallucinogen dependence, continuous
304.52	ICD-9	Substance	Hallucinogen dependence, episodic
304.53	ICD-9	Substance	Hallucinogen dependence, in remission
304.60	ICD-9	Substance	Other specified drug dependence, unspecified
304.61	ICD-9	Substance	Other specified drug dependence, continuous
304.62	ICD-9	Substance	Other specified drug dependence, episodic
304.63	ICD-9	Substance	Other specified drug dependence, in remission
304.70	ICD-9	Substance	Combinations of opioid type drug with any other drug dependence, unspecified
304.71	ICD-9	Substance	Combinations of opioid type drug with any other drug dependence, continuous
304.72	ICD-9	Substance	Combinations of opioid type drug with any other drug dependence, episodic
304.73	ICD-9	Substance	Combinations of opioid type drug with any other drug dependence, in remission
304.80	ICD-9	Substance	Combinations of drug dependence excluding opioid type drug, unspecified
304.81	ICD-9	Substance	Combinations of drug dependence excluding opioid type drug, continuous
304.82	ICD-9	Substance	Combinations of drug dependence excluding opioid type drug, episodic
304.83	ICD-9	Substance	Combinations of drug dependence excluding opioid type drug, in remission
304.90	ICD-9	Substance	Unspecified drug dependence, unspecified
304.91	ICD-9	Substance	Unspecified drug dependence, continuous
304.92	ICD-9	Substance	Unspecified drug dependence, episodic

© 2023 Brenner LA et al. *JAMA Network Open.*

304.93	ICD-9	Substance	Unspecified drug dependence, in remission
305.20	ICD-9	Substance	Cannabis abuse, unspecified
305.21	ICD-9	Substance	Cannabis abuse, continuous
305.22	ICD-9	Substance	Cannabis abuse, episodic
305.23	ICD-9	Substance	Cannabis abuse, in remission
305.30	ICD-9	Substance	Hallucinogen abuse, unspecified
305.31	ICD-9	Substance	Hallucinogen abuse, continuous
305.32	ICD-9	Substance	Hallucinogen abuse, episodic
305.33	ICD-9	Substance	Hallucinogen abuse, in remission
305.40	ICD-9	Substance	Sedative, hypnotic or anxiolytic abuse, unspecified
305.41	ICD-9	Substance	Sedative, hypnotic or anxiolytic abuse, continuous
305.42	ICD-9	Substance	Sedative, hypnotic or anxiolytic abuse, episodic
305.43	ICD-9	Substance	Sedative, hypnotic or anxiolytic abuse, in remission
305.50	ICD-9	Substance	Opioid abuse, unspecified
305.51	ICD-9	Substance	Opioid abuse, continuous
305.52	ICD-9	Substance	Opioid abuse, episodic
305.53	ICD-9	Substance	Opioid abuse, in remission
305.60	ICD-9	Substance	Cocaine abuse, unspecified
305.61	ICD-9	Substance	Cocaine abuse, continuous
305.62	ICD-9	Substance	Cocaine abuse, episodic
305.63	ICD-9	Substance	Cocaine abuse, in remission
305.70	ICD-9	Substance	Amphetamine or related acting sympathomimetic abuse, unspecified
305.71	ICD-9	Substance	Amphetamine or related acting sympathomimetic abuse, continuous
305.72	ICD-9	Substance	Amphetamine or related acting sympathomimetic abuse, episodic
305.73	ICD-9	Substance	Amphetamine or related acting sympathomimetic abuse, in remission
305.80	ICD-9	Substance	Antidepressant type abuse, unspecified
305.81	ICD-9	Substance	Antidepressant type abuse, continuous
305.82	ICD-9	Substance	Antidepressant type abuse, episodic
305.83	ICD-9	Substance	Antidepressant type abuse, in remission
305.90	ICD-9	Substance	Other, mixed, or unspecified drug abuse, unspecified
305.91	ICD-9	Substance	Other, mixed, or unspecified drug abuse, continuous
305.92	ICD-9	Substance	Other, mixed, or unspecified drug abuse, episodic
305.93	ICD-9	Substance	Other, mixed, or unspecified drug abuse, in remission
648.30	ICD-9	Substance	Drug dependence of mother, unspecified as to episode of care or not applicable

© 2023 Brenner LA et al. *JAMA Network Open.*

648.31	ICD-9	Substance	Drug dependence of mother, delivered, with or without mention of antepartum condition
648.32	ICD-9	Substance	Drug dependence of mother, delivered, with mention of postpartum complication
648.33	ICD-9	Substance	Drug dependence of mother, antepartum condition or complication
648.34	ICD-9	Substance	Drug dependence of mother, postpartum condition or complication
655.50	ICD-9	Substance	Suspected damage to fetus from drugs, affecting management of mother, unspecified as to episode of care or not applicable
655.51	ICD-9	Substance	Suspected damage to fetus from drugs, affecting management of mother, delivered, with or without mention of antepartum condition
655.53	ICD-9	Substance	Suspected damage to fetus from drugs, affecting management of mother, antepartum condition or complication
F11.10	ICD-10	Substance	Opioid abuse, uncomplicated
F11.11	ICD-10	Substance	Opioid abuse, in remission
F11.120	ICD-10	Substance	Opioid abuse with intoxication, uncomplicated
F11.121	ICD-10	Substance	Opioid abuse with intoxication delirium
F11.122	ICD-10	Substance	Opioid abuse with intoxication with perceptual disturbance
F11.129	ICD-10	Substance	Opioid abuse with intoxication, unspecified
F11.13	ICD-10	Substance	Opioid abuse with withdrawal
F11.14	ICD-10	Substance	Opioid abuse with opioid-induced mood disorder
F11.150	ICD-10	Substance	Opioid abuse with opioid-induced psychotic disorder with delusions
F11.151	ICD-10	Substance	Opioid abuse with opioid-induced psychotic disorder with hallucinations
F11.159	ICD-10	Substance	Opioid abuse with opioid-induced psychotic disorder, unspecified
F11.181	ICD-10	Substance	Opioid abuse with opioid-induced sexual dysfunction
F11.182	ICD-10	Substance	Opioid abuse with opioid-induced sleep disorder
F11.188	ICD-10	Substance	Opioid abuse with other opioid-induced disorder
F11.19	ICD-10	Substance	Opioid abuse with unspecified opioid-induced disorder
F11.20	ICD-10	Substance	Opioid dependence, uncomplicated
F11.21	ICD-10	Substance	Opioid dependence, in remission
F11.220	ICD-10	Substance	Opioid dependence with intoxication, uncomplicated
F11.221	ICD-10	Substance	Opioid dependence with intoxication delirium
F11.222	ICD-10	Substance	Opioid dependence with intoxication with perceptual disturbance
F11.229	ICD-10	Substance	Opioid dependence with intoxication, unspecified
F11.23	ICD-10	Substance	Opioid dependence with withdrawal
F11.24	ICD-10	Substance	Opioid dependence with opioid-induced mood disorder
F11.250	ICD-10	Substance	Opioid dependence with opioid-induced psychotic disorder with delusions
F11.251	ICD-10	Substance	Opioid dependence with opioid-induced psychotic disorder with hallucinations

© 2023 Brenner LA et al. *JAMA Network Open.*

F11.259	ICD-10	Substance	Opioid dependence with opioid-induced psychotic disorder, unspecified
F11.281	ICD-10	Substance	Opioid dependence with opioid-induced sexual dysfunction
F11.282	ICD-10	Substance	Opioid dependence with opioid-induced sleep disorder
F11.288	ICD-10	Substance	Opioid dependence with other opioid-induced disorder
F11.29	ICD-10	Substance	Opioid dependence with unspecified opioid-induced disorder
F11.90	ICD-10	Substance	Opioid use, unspecified, uncomplicated
F11.920	ICD-10	Substance	Opioid use, unspecified with intoxication, uncomplicated
F11.921	ICD-10	Substance	Opioid use, unspecified with intoxication delirium
F11.922	ICD-10	Substance	Opioid use, unspecified with intoxication with perceptual disturbance
F11.929	ICD-10	Substance	Opioid use, unspecified with intoxication, unspecified
F11.93	ICD-10	Substance	Opioid use, unspecified with withdrawal
F11.94	ICD-10	Substance	Opioid use, unspecified with opioid-induced mood disorder
F11.950	ICD-10	Substance	Opioid use, unspecified with opioid-induced psychotic disorder with delusions
F11.951	ICD-10	Substance	Opioid use, unspecified with opioid-induced psychotic disorder with hallucinations
F11.959	ICD-10	Substance	Opioid use, unspecified with opioid-induced psychotic disorder, unspecified
F11.981	ICD-10	Substance	Opioid use, unspecified with opioid-induced sexual dysfunction
F11.982	ICD-10	Substance	Opioid use, unspecified with opioid-induced sleep disorder
F11.988	ICD-10	Substance	Opioid use, unspecified with other opioid-induced disorder
F11.99	ICD-10	Substance	Opioid use, unspecified with unspecified opioid-induced disorder
F12.10	ICD-10	Substance	Cannabis abuse, uncomplicated
F12.11	ICD-10	Substance	Cannabis abuse, in remission
F12.120	ICD-10	Substance	Cannabis abuse with intoxication, uncomplicated
F12.121	ICD-10	Substance	Cannabis abuse with intoxication delirium
F12.122	ICD-10	Substance	Cannabis abuse with intoxication with perceptual disturbance
F12.129	ICD-10	Substance	Cannabis abuse with intoxication, unspecified
F12.13	ICD-10	Substance	Cannabis abuse with withdrawal
F12.150	ICD-10	Substance	Cannabis abuse with psychotic disorder with delusions
F12.151	ICD-10	Substance	Cannabis abuse with psychotic disorder with hallucinations
F12.159	ICD-10	Substance	Cannabis abuse with psychotic disorder, unspecified
F12.180	ICD-10	Substance	Cannabis abuse with cannabis-induced anxiety disorder
F12.188	ICD-10	Substance	Cannabis abuse with other cannabis-induced disorder
F12.19	ICD-10	Substance	Cannabis abuse with unspecified cannabis-induced disorder
F12.20	ICD-10	Substance	Cannabis dependence, uncomplicated
F12.21	ICD-10	Substance	Cannabis dependence, in remission

© 2023 Brenner LA et al. *JAMA Network Open.*

F12.220	ICD-10	Substance	Cannabis dependence with intoxication, uncomplicated
F12.221	ICD-10	Substance	Cannabis dependence with intoxication delirium
F12.222	ICD-10	Substance	Cannabis dependence with intoxication with perceptual disturbance
F12.229	ICD-10	Substance	Cannabis dependence with intoxication, unspecified
F12.23	ICD-10	Substance	Cannabis dependence with withdrawal
F12.250	ICD-10	Substance	Cannabis dependence with psychotic disorder with delusions
F12.251	ICD-10	Substance	Cannabis dependence with psychotic disorder with hallucinations
F12.259	ICD-10	Substance	Cannabis dependence with psychotic disorder, unspecified
F12.280	ICD-10	Substance	Cannabis dependence with cannabis-induced anxiety disorder
F12.288	ICD-10	Substance	Cannabis dependence with other cannabis-induced disorder
F12.29	ICD-10	Substance	Cannabis dependence with unspecified cannabis-induced disorder
F12.90	ICD-10	Substance	Cannabis use, unspecified, uncomplicated
F12.920	ICD-10	Substance	Cannabis use, unspecified with intoxication, uncomplicated
F12.921	ICD-10	Substance	Cannabis use, unspecified with intoxication delirium
F12.922	ICD-10	Substance	Cannabis use, unspecified with intoxication with perceptual disturbance
F12.929	ICD-10	Substance	Cannabis use, unspecified with intoxication, unspecified
F12.93	ICD-10	Substance	Cannabis use, unspecified with withdrawal
F12.950	ICD-10	Substance	Cannabis use, unspecified with psychotic disorder with delusions
F12.951	ICD-10	Substance	Cannabis use, unspecified with psychotic disorder with hallucinations
F12.959	ICD-10	Substance	Cannabis use, unspecified with psychotic disorder, unspecified
F12.980	ICD-10	Substance	Cannabis use, unspecified with anxiety disorder
F12.988	ICD-10	Substance	Cannabis use, unspecified with other cannabis-induced disorder
F12.99	ICD-10	Substance	Cannabis use, unspecified with unspecified cannabis-induced disorder
F13.10	ICD-10	Substance	Sedative, hypnotic or anxiolytic abuse, uncomplicated
F13.11	ICD-10	Substance	Sedative, hypnotic or anxiolytic abuse, in remission
F13.120	ICD-10	Substance	Sedative, hypnotic or anxiolytic abuse with intoxication, uncomplicated
F13.121	ICD-10	Substance	Sedative, hypnotic or anxiolytic abuse with intoxication delirium
F13.129	ICD-10	Substance	Sedative, hypnotic or anxiolytic abuse with intoxication, unspecified
F13.130	ICD-10	Substance	Sedative, hypnotic or anxiolytic abuse with withdrawal, uncomplicated
F13.131	ICD-10	Substance	Sedative, hypnotic or anxiolytic abuse with withdrawal delirium
F13.132	ICD-10	Substance	Sedative, hypnotic or anxiolytic abuse with withdrawal with perceptual disturbance
F13.139	ICD-10	Substance	Sedative, hypnotic or anxiolytic abuse with withdrawal, unspecified
F13.14	ICD-10	Substance	Sedative, hypnotic or anxiolytic abuse with sedative, hypnotic or anxiolytic-induced mood disorder

© 2023 Brenner LA et al. *JAMA Network Open.*

F13.150	ICD-10	Substance	Sedative, hypnotic or anxiolytic abuse with sedative, hypnotic or anxiolytic-induced psychotic disorder with delusions
F13.151	ICD-10	Substance	Sedative, hypnotic or anxiolytic abuse with sedative, hypnotic or anxiolytic-induced psychotic disorder with hallucinations
F13.159	ICD-10	Substance	Sedative, hypnotic or anxiolytic abuse with sedative, hypnotic or anxiolytic-induced psychotic disorder, unspecified
F13.180	ICD-10	Substance	Sedative, hypnotic or anxiolytic abuse with sedative, hypnotic or anxiolytic-induced anxiety disorder
F13.181	ICD-10	Substance	Sedative, hypnotic or anxiolytic abuse with sedative, hypnotic or anxiolytic-induced sexual dysfunction
F13.182	ICD-10	Substance	Sedative, hypnotic or anxiolytic abuse with sedative, hypnotic or anxiolytic-induced sleep disorder
F13.188	ICD-10	Substance	Sedative, hypnotic or anxiolytic abuse with other sedative, hypnotic or anxiolytic-induced disorder
F13.19	ICD-10	Substance	Sedative, hypnotic or anxiolytic abuse with unspecified sedative, hypnotic or anxiolytic-induced disorder
F13.20	ICD-10	Substance	Sedative, hypnotic or anxiolytic dependence, uncomplicated
F13.21	ICD-10	Substance	Sedative, hypnotic or anxiolytic dependence, in remission
F13.220	ICD-10	Substance	Sedative, hypnotic or anxiolytic dependence with intoxication, uncomplicated
F13.221	ICD-10	Substance	Sedative, hypnotic or anxiolytic dependence with intoxication delirium
F13.229	ICD-10	Substance	Sedative, hypnotic or anxiolytic dependence with intoxication, unspecified
F13.230	ICD-10	Substance	Sedative, hypnotic or anxiolytic dependence with withdrawal, uncomplicated
F13.231	ICD-10	Substance	Sedative, hypnotic or anxiolytic dependence with withdrawal delirium
F13.232	ICD-10	Substance	Sedative, hypnotic or anxiolytic dependence with withdrawal with perceptual disturbance
F13.239	ICD-10	Substance	Sedative, hypnotic or anxiolytic dependence with withdrawal, unspecified
F13.24	ICD-10	Substance	Sedative, hypnotic or anxiolytic dependence with sedative, hypnotic or anxiolytic-induced mood disorder
F13.250	ICD-10	Substance	Sedative, hypnotic or anxiolytic dependence with sedative, hypnotic or anxiolytic-induced psychotic disorder with delusions
F13.251	ICD-10	Substance	Sedative, hypnotic or anxiolytic dependence with sedative, hypnotic or anxiolytic-induced psychotic disorder with hallucinations
F13.259	ICD-10	Substance	Sedative, hypnotic or anxiolytic dependence with sedative, hypnotic or anxiolytic-induced psychotic disorder, unspecified
F13.26	ICD-10	Substance	Sedative, hypnotic or anxiolytic dependence with sedative, hypnotic or anxiolytic-induced persisting amnestic disorder
F13.27	ICD-10	Substance	Sedative, hypnotic or anxiolytic dependence with sedative, hypnotic or anxiolytic-induced persisting dementia

© 2023 Brenner LA et al. *JAMA Network Open.*

F13.280	ICD-10	Substance	Sedative, hypnotic or anxiolytic dependence with sedative, hypnotic or anxiolytic-induced anxiety disorder
F13.281	ICD-10	Substance	Sedative, hypnotic or anxiolytic dependence with sedative, hypnotic or anxiolytic-induced sexual dysfunction
F13.282	ICD-10	Substance	Sedative, hypnotic or anxiolytic dependence with sedative, hypnotic or anxiolytic-induced sleep disorder
F13.288	ICD-10	Substance	Sedative, hypnotic or anxiolytic dependence with other sedative, hypnotic or anxiolytic-induced disorder
F13.29	ICD-10	Substance	Sedative, hypnotic or anxiolytic dependence with unspecified sedative, hypnotic or anxiolytic-induced disorder
F13.90	ICD-10	Substance	Sedative, hypnotic, or anxiolytic use, unspecified, uncomplicated
F13.920	ICD-10	Substance	Sedative, hypnotic or anxiolytic use, unspecified with intoxication, uncomplicated
F13.921	ICD-10	Substance	Sedative, hypnotic or anxiolytic use, unspecified with intoxication delirium
F13.929	ICD-10	Substance	Sedative, hypnotic or anxiolytic use, unspecified with intoxication, unspecified
F13.930	ICD-10	Substance	Sedative, hypnotic or anxiolytic use, unspecified with withdrawal, uncomplicated
F13.931	ICD-10	Substance	Sedative, hypnotic or anxiolytic use, unspecified with withdrawal delirium
F13.932	ICD-10	Substance	Sedative, hypnotic or anxiolytic use, unspecified with withdrawal with perceptual disturbances
F13.939	ICD-10	Substance	Sedative, hypnotic or anxiolytic use, unspecified with withdrawal, unspecified
F13.94	ICD-10	Substance	Sedative, hypnotic or anxiolytic use, unspecified with sedative, hypnotic or anxiolytic-induced mood disorder
F13.950	ICD-10	Substance	Sedative, hypnotic or anxiolytic use, unspecified with sedative, hypnotic or anxiolytic-induced psychotic disorder with delusions
F13.951	ICD-10	Substance	Sedative, hypnotic or anxiolytic use, unspecified with sedative, hypnotic or anxiolytic-induced psychotic disorder with hallucinations
F13.959	ICD-10	Substance	Sedative, hypnotic or anxiolytic use, unspecified with sedative, hypnotic or anxiolytic-induced psychotic disorder, unspecified
F13.96	ICD-10	Substance	Sedative, hypnotic or anxiolytic use, unspecified with sedative, hypnotic or anxiolytic-induced persisting amnestic disorder
F13.97	ICD-10	Substance	Sedative, hypnotic or anxiolytic use, unspecified with sedative, hypnotic or anxiolytic-induced persisting dementia
F13.980	ICD-10	Substance	Sedative, hypnotic or anxiolytic use, unspecified with sedative, hypnotic or anxiolytic-induced anxiety disorder
F13.981	ICD-10	Substance	Sedative, hypnotic or anxiolytic use, unspecified with sedative, hypnotic or anxiolytic-induced sexual dysfunction
F13.982	ICD-10	Substance	Sedative, hypnotic or anxiolytic use, unspecified with sedative, hypnotic or anxiolytic-induced sleep disorder
F13.988	ICD-10	Substance	Sedative, hypnotic or anxiolytic use, unspecified with other sedative, hypnotic or anxiolytic-induced disorder

© 2023 Brenner LA et al. *JAMA Network Open.*

F13.99	ICD-10	Substance	Sedative, hypnotic or anxiolytic use, unspecified with unspecified sedative, hypnotic or anxiolytic-induced disorder
F14.10	ICD-10	Substance	Cocaine abuse, uncomplicated
F14.11	ICD-10	Substance	Cocaine abuse, in remission
F14.120	ICD-10	Substance	Cocaine abuse with intoxication, uncomplicated
F14.121	ICD-10	Substance	Cocaine abuse with intoxication with delirium
F14.122	ICD-10	Substance	Cocaine abuse with intoxication with perceptual disturbance
F14.129	ICD-10	Substance	Cocaine abuse with intoxication, unspecified
F14.13	ICD-10	Substance	Cocaine abuse, unspecified with withdrawal
F14.14	ICD-10	Substance	Cocaine abuse with cocaine-induced mood disorder
F14.150	ICD-10	Substance	Cocaine abuse with cocaine-induced psychotic disorder with delusions
F14.151	ICD-10	Substance	Cocaine abuse with cocaine-induced psychotic disorder with hallucinations
F14.159	ICD-10	Substance	Cocaine abuse with cocaine-induced psychotic disorder, unspecified
F14.180	ICD-10	Substance	Cocaine abuse with cocaine-induced anxiety disorder
F14.181	ICD-10	Substance	Cocaine abuse with cocaine-induced sexual dysfunction
F14.182	ICD-10	Substance	Cocaine abuse with cocaine-induced sleep disorder
F14.188	ICD-10	Substance	Cocaine abuse with other cocaine-induced disorder
F14.19	ICD-10	Substance	Cocaine abuse with unspecified cocaine-induced disorder
F14.20	ICD-10	Substance	Cocaine dependence, uncomplicated
F14.21	ICD-10	Substance	Cocaine dependence, in remission
F14.220	ICD-10	Substance	Cocaine dependence with intoxication, uncomplicated
F14.221	ICD-10	Substance	Cocaine dependence with intoxication delirium
F14.222	ICD-10	Substance	Cocaine dependence with intoxication with perceptual disturbance
F14.229	ICD-10	Substance	Cocaine dependence with intoxication, unspecified
F14.23	ICD-10	Substance	Cocaine dependence with withdrawal
F14.24	ICD-10	Substance	Cocaine dependence with cocaine-induced mood disorder
F14.250	ICD-10	Substance	Cocaine dependence with cocaine-induced psychotic disorder with delusions
F14.251	ICD-10	Substance	Cocaine dependence with cocaine-induced psychotic disorder with hallucinations
F14.259	ICD-10	Substance	Cocaine dependence with cocaine-induced psychotic disorder, unspecified
F14.280	ICD-10	Substance	Cocaine dependence with cocaine-induced anxiety disorder
F14.281	ICD-10	Substance	Cocaine dependence with cocaine-induced sexual dysfunction
F14.282	ICD-10	Substance	Cocaine dependence with cocaine-induced sleep disorder
F14.288	ICD-10	Substance	Cocaine dependence with other cocaine-induced disorder
F14.29	ICD-10	Substance	Cocaine dependence with unspecified cocaine-induced disorder

© 2023 Brenner LA et al. *JAMA Network Open.*

F14.90	ICD-10	Substance	Cocaine use, unspecified, uncomplicated
F14.920	ICD-10	Substance	Cocaine use, unspecified with intoxication, uncomplicated
F14.921	ICD-10	Substance	Cocaine use, unspecified with intoxication delirium
F14.922	ICD-10	Substance	Cocaine use, unspecified with intoxication with perceptual disturbance
F14.929	ICD-10	Substance	Cocaine use, unspecified with intoxication, unspecified
F14.93	ICD-10	Substance	Cocaine use, unspecified with withdrawal
F14.94	ICD-10	Substance	Cocaine use, unspecified with cocaine-induced mood disorder
F14.950	ICD-10	Substance	Cocaine use, unspecified with cocaine-induced psychotic disorder with delusions
F14.951	ICD-10	Substance	Cocaine use, unspecified with cocaine-induced psychotic disorder with hallucinations
F14.959	ICD-10	Substance	Cocaine use, unspecified with cocaine-induced psychotic disorder, unspecified
F14.980	ICD-10	Substance	Cocaine use, unspecified with cocaine-induced anxiety disorder
F14.981	ICD-10	Substance	Cocaine use, unspecified with cocaine-induced sexual dysfunction
F14.982	ICD-10	Substance	Cocaine use, unspecified with cocaine-induced sleep disorder
F14.988	ICD-10	Substance	Cocaine use, unspecified with other cocaine-induced disorder
F14.99	ICD-10	Substance	Cocaine use, unspecified with unspecified cocaine-induced disorder
F15.10	ICD-10	Substance	Other stimulant abuse, uncomplicated
F15.11	ICD-10	Substance	Other stimulant abuse, in remission
F15.120	ICD-10	Substance	Other stimulant abuse with intoxication, uncomplicated
F15.121	ICD-10	Substance	Other stimulant abuse with intoxication delirium
F15.122	ICD-10	Substance	Other stimulant abuse with intoxication with perceptual disturbance
F15.129	ICD-10	Substance	Other stimulant abuse with intoxication, unspecified
F15.13	ICD-10	Substance	Other stimulant abuse with withdrawal
F15.14	ICD-10	Substance	Other stimulant abuse with stimulant-induced mood disorder
F15.150	ICD-10	Substance	Other stimulant abuse with stimulant-induced psychotic disorder with delusions
F15.151	ICD-10	Substance	Other stimulant abuse with stimulant-induced psychotic disorder with hallucinations
F15.159	ICD-10	Substance	Other stimulant abuse with stimulant-induced psychotic disorder, unspecified
F15.180	ICD-10	Substance	Other stimulant abuse with stimulant-induced anxiety disorder
F15.181	ICD-10	Substance	Other stimulant abuse with stimulant-induced sexual dysfunction
F15.182	ICD-10	Substance	Other stimulant abuse with stimulant-induced sleep disorder
F15.188	ICD-10	Substance	Other stimulant abuse with other stimulant-induced disorder
F15.19	ICD-10	Substance	Other stimulant abuse with unspecified stimulant-induced disorder
F15.20	ICD-10	Substance	Other stimulant dependence, uncomplicated
F15.21	ICD-10	Substance	Other stimulant dependence, in remission
F15.220	ICD-10	Substance	Other stimulant dependence with intoxication, uncomplicated

© 2023 Brenner LA et al. *JAMA Network Open.*

F15.221	ICD-10	Substance	Other stimulant dependence with intoxication delirium
F15.222	ICD-10	Substance	Other stimulant dependence with intoxication with perceptual disturbance
F15.229	ICD-10	Substance	Other stimulant dependence with intoxication, unspecified
F15.23	ICD-10	Substance	Other stimulant dependence with withdrawal
F15.24	ICD-10	Substance	Other stimulant dependence with stimulant-induced mood disorder
F15.250	ICD-10	Substance	Other stimulant dependence with stimulant-induced psychotic disorder with delusions
F15.251	ICD-10	Substance	Other stimulant dependence with stimulant-induced psychotic disorder with hallucinations
F15.259	ICD-10	Substance	Other stimulant dependence with stimulant-induced psychotic disorder, unspecified
F15.280	ICD-10	Substance	Other stimulant dependence with stimulant-induced anxiety disorder
F15.281	ICD-10	Substance	Other stimulant dependence with stimulant-induced sexual dysfunction
F15.282	ICD-10	Substance	Other stimulant dependence with stimulant-induced sleep disorder
F15.288	ICD-10	Substance	Other stimulant dependence with other stimulant-induced disorder
F15.29	ICD-10	Substance	Other stimulant dependence with unspecified stimulant-induced disorder
F15.90	ICD-10	Substance	Other stimulant use, unspecified, uncomplicated
F15.920	ICD-10	Substance	Other stimulant use, unspecified with intoxication, uncomplicated
F15.921	ICD-10	Substance	Other stimulant use, unspecified with intoxication delirium
F15.922	ICD-10	Substance	Other stimulant use, unspecified with intoxication with perceptual disturbance
F15.929	ICD-10	Substance	Other stimulant use, unspecified with intoxication, unspecified
F15.93	ICD-10	Substance	Other stimulant use, unspecified with withdrawal
F15.94	ICD-10	Substance	Other stimulant use, unspecified with stimulant-induced mood disorder
F15.950	ICD-10	Substance	Other stimulant use, unspecified with stimulant-induced psychotic disorder with delusions
F15.951	ICD-10	Substance	Other stimulant use, unspecified with stimulant-induced psychotic disorder with hallucinations
F15.959	ICD-10	Substance	Other stimulant use, unspecified with stimulant-induced psychotic disorder, unspecified
F15.980	ICD-10	Substance	Other stimulant use, unspecified with stimulant-induced anxiety disorder
F15.981	ICD-10	Substance	Other stimulant use, unspecified with stimulant-induced sexual dysfunction
F15.982	ICD-10	Substance	Other stimulant use, unspecified with stimulant-induced sleep disorder
F15.988	ICD-10	Substance	Other stimulant use, unspecified with other stimulant-induced disorder
F15.99	ICD-10	Substance	Other stimulant use, unspecified with unspecified stimulant-induced disorder
F16.10	ICD-10	Substance	Hallucinogen abuse, uncomplicated
F16.11	ICD-10	Substance	Hallucinogen abuse, in remission
F16.120	ICD-10	Substance	Hallucinogen abuse with intoxication, uncomplicated
F16.121	ICD-10	Substance	Hallucinogen abuse with intoxication with delirium
F16.122	ICD-10	Substance	Hallucinogen abuse with intoxication with perceptual disturbance
F16.129	ICD-10	Substance	Hallucinogen abuse with intoxication, unspecified

© 2023 Brenner LA et al. *JAMA Network Open.*

F16.14	ICD-10	Substance	Hallucinogen abuse with hallucinogen-induced mood disorder
F16.150	ICD-10	Substance	Hallucinogen abuse with hallucinogen-induced psychotic disorder with delusions
F16.151	ICD-10	Substance	Hallucinogen abuse with hallucinogen-induced psychotic disorder with hallucinations
F16.159	ICD-10	Substance	Hallucinogen abuse with hallucinogen-induced psychotic disorder, unspecified
F16.180	ICD-10	Substance	Hallucinogen abuse with hallucinogen-induced anxiety disorder
F16.183	ICD-10	Substance	Hallucinogen abuse with hallucinogen persisting perception disorder (flashbacks)
F16.188	ICD-10	Substance	Hallucinogen abuse with other hallucinogen-induced disorder
F16.19	ICD-10	Substance	Hallucinogen abuse with unspecified hallucinogen-induced disorder
F16.20	ICD-10	Substance	Hallucinogen dependence, uncomplicated
F16.21	ICD-10	Substance	Hallucinogen dependence, in remission
F16.220	ICD-10	Substance	Hallucinogen dependence with intoxication, uncomplicated
F16.221	ICD-10	Substance	Hallucinogen dependence with intoxication with delirium
F16.229	ICD-10	Substance	Hallucinogen dependence with intoxication, unspecified
F16.24	ICD-10	Substance	Hallucinogen dependence with hallucinogen-induced mood disorder
F16.250	ICD-10	Substance	Hallucinogen dependence with hallucinogen-induced psychotic disorder with delusions
F16.251	ICD-10	Substance	Hallucinogen dependence with hallucinogen-induced psychotic disorder with hallucinations
F16.259	ICD-10	Substance	Hallucinogen dependence with hallucinogen-induced psychotic disorder, unspecified
F16.280	ICD-10	Substance	Hallucinogen dependence with hallucinogen-induced anxiety disorder
F16.283	ICD-10	Substance	Hallucinogen dependence with hallucinogen persisting perception disorder (flashbacks)
F16.288	ICD-10	Substance	Hallucinogen dependence with other hallucinogen-induced disorder
F16.29	ICD-10	Substance	Hallucinogen dependence with unspecified hallucinogen-induced disorder
F16.90	ICD-10	Substance	Hallucinogen use, unspecified, uncomplicated
F16.920	ICD-10	Substance	Hallucinogen use, unspecified with intoxication, uncomplicated
F16.921	ICD-10	Substance	Hallucinogen use, unspecified with intoxication with delirium
F16.929	ICD-10	Substance	Hallucinogen use, unspecified with intoxication, unspecified
F16.94	ICD-10	Substance	Hallucinogen use, unspecified with hallucinogen-induced mood disorder
F16.950	ICD-10	Substance	Hallucinogen use, unspecified with hallucinogen-induced psychotic disorder with delusions
F16.951	ICD-10	Substance	Hallucinogen use, unspecified with hallucinogen-induced psychotic disorder with hallucinations
F16.959	ICD-10	Substance	Hallucinogen use, unspecified with hallucinogen-induced psychotic disorder, unspecified
F16.980	ICD-10	Substance	Hallucinogen use, unspecified with hallucinogen-induced anxiety disorder
F16.983	ICD-10	Substance	Hallucinogen use, unspecified with hallucinogen persisting perception disorder (flashbacks)
F16.988	ICD-10	Substance	Hallucinogen use, unspecified with other hallucinogen-induced disorder
F16.99	ICD-10	Substance	Hallucinogen use, unspecified with unspecified hallucinogen-induced disorder
F18.10	ICD-10	Substance	Inhalant abuse, uncomplicated

© 2023 Brenner LA et al. *JAMA Network Open.*

F18.11	ICD-10	Substance	Inhalant abuse, in remission
F18.120	ICD-10	Substance	Inhalant abuse with intoxication, uncomplicated
F18.121	ICD-10	Substance	Inhalant abuse with intoxication delirium
F18.129	ICD-10	Substance	Inhalant abuse with intoxication, unspecified
F18.14	ICD-10	Substance	Inhalant abuse with inhalant-induced mood disorder
F18.150	ICD-10	Substance	Inhalant abuse with inhalant-induced psychotic disorder with delusions
F18.151	ICD-10	Substance	Inhalant abuse with inhalant-induced psychotic disorder with hallucinations
F18.159	ICD-10	Substance	Inhalant abuse with inhalant-induced psychotic disorder, unspecified
F18.17	ICD-10	Substance	Inhalant abuse with inhalant-induced dementia
F18.180	ICD-10	Substance	Inhalant abuse with inhalant-induced anxiety disorder
F18.188	ICD-10	Substance	Inhalant abuse with other inhalant-induced disorder
F18.19	ICD-10	Substance	Inhalant abuse with unspecified inhalant-induced disorder
F18.20	ICD-10	Substance	Inhalant dependence, uncomplicated
F18.21	ICD-10	Substance	Inhalant dependence, in remission
F18.220	ICD-10	Substance	Inhalant dependence with intoxication, uncomplicated
F18.221	ICD-10	Substance	Inhalant dependence with intoxication delirium
F18.229	ICD-10	Substance	Inhalant dependence with intoxication, unspecified
F18.24	ICD-10	Substance	Inhalant dependence with inhalant-induced mood disorder
F18.250	ICD-10	Substance	Inhalant dependence with inhalant-induced psychotic disorder with delusions
F18.251	ICD-10	Substance	Inhalant dependence with inhalant-induced psychotic disorder with hallucinations
F18.259	ICD-10	Substance	Inhalant dependence with inhalant-induced psychotic disorder, unspecified
F18.27	ICD-10	Substance	Inhalant dependence with inhalant-induced dementia
F18.280	ICD-10	Substance	Inhalant dependence with inhalant-induced anxiety disorder
F18.288	ICD-10	Substance	Inhalant dependence with other inhalant-induced disorder
F18.29	ICD-10	Substance	Inhalant dependence with unspecified inhalant-induced disorder
F18.90	ICD-10	Substance	Inhalant use, unspecified, uncomplicated
F18.920	ICD-10	Substance	Inhalant use, unspecified with intoxication, uncomplicated
F18.921	ICD-10	Substance	Inhalant use, unspecified with intoxication with delirium
F18.929	ICD-10	Substance	Inhalant use, unspecified with intoxication, unspecified
F18.94	ICD-10	Substance	Inhalant use, unspecified with inhalant-induced mood disorder
F18.950	ICD-10	Substance	Inhalant use, unspecified with inhalant-induced psychotic disorder with delusions
F18.951	ICD-10	Substance	Inhalant use, unspecified with inhalant-induced psychotic disorder with hallucinations
F18.959	ICD-10	Substance	Inhalant use, unspecified with inhalant-induced psychotic disorder, unspecified
F18.97	ICD-10	Substance	Inhalant use, unspecified with inhalant-induced persisting dementia

© 2023 Brenner LA et al. *JAMA Network Open.*

F18.980	ICD-10	Substance	Inhalant use, unspecified with inhalant-induced anxiety disorder
F18.988	ICD-10	Substance	Inhalant use, unspecified with other inhalant-induced disorder
F18.99	ICD-10	Substance	Inhalant use, unspecified with unspecified inhalant-induced disorder
F19.10	ICD-10	Substance	Other psychoactive substance abuse, uncomplicated
F19.11	ICD-10	Substance	Other psychoactive substance abuse, in remission
F19.120	ICD-10	Substance	Other psychoactive substance abuse with intoxication, uncomplicated
F19.121	ICD-10	Substance	Other psychoactive substance abuse with intoxication delirium
F19.122	ICD-10	Substance	Other psychoactive substance abuse with intoxication with perceptual disturbances
F19.129	ICD-10	Substance	Other psychoactive substance abuse with intoxication, unspecified
F19.130	ICD-10	Substance	Other psychoactive substance abuse with withdrawal, uncomplicated
F19.131	ICD-10	Substance	Other psychoactive substance abuse with withdrawal delirium
F19.132	ICD-10	Substance	Other psychoactive substance abuse with withdrawal with perceptual disturbance
F19.139	ICD-10	Substance	Other psychoactive substance abuse with withdrawal, unspecified
F19.14	ICD-10	Substance	Other psychoactive substance abuse with psychoactive substance-induced mood disorder
F19.150	ICD-10	Substance	Other psychoactive substance abuse with psychoactive substance-induced psychotic disorder with delusions
F19.151	ICD-10	Substance	Other psychoactive substance abuse with psychoactive substance-induced psychotic disorder with hallucinations
F19.159	ICD-10	Substance	Other psychoactive substance abuse with psychoactive substance-induced psychotic disorder, unspecified
F19.16	ICD-10	Substance	Other psychoactive substance abuse with psychoactive substance-induced persisting amnestic disorder
F19.17	ICD-10	Substance	Other psychoactive substance abuse with psychoactive substance-induced persisting dementia
F19.180	ICD-10	Substance	Other psychoactive substance abuse with psychoactive substance-induced anxiety disorder
F19.181	ICD-10	Substance	Other psychoactive substance abuse with psychoactive substance-induced sexual dysfunction
F19.182	ICD-10	Substance	Other psychoactive substance abuse with psychoactive substance-induced sleep disorder
F19.188	ICD-10	Substance	Other psychoactive substance abuse with other psychoactive substance-induced disorder
F19.19	ICD-10	Substance	Other psychoactive substance abuse with unspecified psychoactive substance-induced disorder
F19.20	ICD-10	Substance	Other psychoactive substance dependence, uncomplicated
F19.21	ICD-10	Substance	Other psychoactive substance dependence, in remission
F19.220	ICD-10	Substance	Other psychoactive substance dependence with intoxication, uncomplicated
F19.221	ICD-10	Substance	Other psychoactive substance dependence with intoxication delirium
F19.222	ICD-10	Substance	Other psychoactive substance dependence with intoxication with perceptual disturbance
F19.229	ICD-10	Substance	Other psychoactive substance dependence with intoxication, unspecified
F19.230	ICD-10	Substance	Other psychoactive substance dependence with withdrawal, uncomplicated

© 2023 Brenner LA et al. *JAMA Network Open.*

F19.231	ICD-10	Substance	Other psychoactive substance dependence with withdrawal delirium
F19.232	ICD-10	Substance	Other psychoactive substance dependence with withdrawal with perceptual disturbance
F19.239	ICD-10	Substance	Other psychoactive substance dependence with withdrawal, unspecified
F19.24	ICD-10	Substance	Other psychoactive substance dependence with psychoactive substance-induced mood disorder
F19.250	ICD-10	Substance	Other psychoactive substance dependence with psychoactive substance-induced psychotic disorder with delusions
F19.251	ICD-10	Substance	Other psychoactive substance dependence with psychoactive substance-induced psychotic disorder with hallucinations
F19.259	ICD-10	Substance	Other psychoactive substance dependence with psychoactive substance-induced psychotic disorder, unspecified
F19.26	ICD-10	Substance	Other psychoactive substance dependence with psychoactive substance-induced persisting amnestic disorder
F19.27	ICD-10	Substance	Other psychoactive substance dependence with psychoactive substance-induced persisting dementia
F19.280	ICD-10	Substance	Other psychoactive substance dependence with psychoactive substance-induced anxiety disorder
F19.281	ICD-10	Substance	Other psychoactive substance dependence with psychoactive substance-induced sexual dysfunction
F19.282	ICD-10	Substance	Other psychoactive substance dependence with psychoactive substance-induced sleep disorder
F19.288	ICD-10	Substance	Other psychoactive substance dependence with other psychoactive substance-induced disorder
F19.29	ICD-10	Substance	Other psychoactive substance dependence with unspecified psychoactive substance-induced disorder
F19.90	ICD-10	Substance	Other psychoactive substance use, unspecified, uncomplicated
F19.920	ICD-10	Substance	Other psychoactive substance use, unspecified with intoxication, uncomplicated
F19.921	ICD-10	Substance	Other psychoactive substance use, unspecified with intoxication with delirium
F19.922	ICD-10	Substance	Other psychoactive substance use, unspecified with intoxication with perceptual disturbance
F19.929	ICD-10	Substance	Other psychoactive substance use, unspecified with intoxication, unspecified
F19.930	ICD-10	Substance	Other psychoactive substance use, unspecified with withdrawal, uncomplicated
F19.931	ICD-10	Substance	Other psychoactive substance use, unspecified with withdrawal delirium
F19.932	ICD-10	Substance	Other psychoactive substance use, unspecified with withdrawal with perceptual disturbance
F19.939	ICD-10	Substance	Other psychoactive substance use, unspecified with withdrawal, unspecified
F19.94	ICD-10	Substance	Other psychoactive substance use, unspecified with psychoactive substance-induced mood disorder
F19.950	ICD-10	Substance	Other psychoactive substance use, unspecified with psychoactive substance-induced psychotic disorder with delusions
F19.951	ICD-10	Substance	Other psychoactive substance use, unspecified with psychoactive substance-induced psychotic disorder with hallucinations

© 2023 Brenner LA et al. *JAMA Network Open.*

F19.959	ICD-10	Substance	Other psychoactive substance use, unspecified with psychoactive substance-induced psychotic disorder, unspecified
F19.96	ICD-10	Substance	Other psychoactive substance use, unspecified with psychoactive substance-induced persisting amnestic disorder
F19.97	ICD-10	Substance	Other psychoactive substance use, unspecified with psychoactive substance-induced persisting dementia
F19.980	ICD-10	Substance	Other psychoactive substance use, unspecified with psychoactive substance-induced anxiety disorder
F19.981	ICD-10	Substance	Other psychoactive substance use, unspecified with psychoactive substance-induced sexual dysfunction
F19.982	ICD-10	Substance	Other psychoactive substance use, unspecified with psychoactive substance-induced sleep disorder
F19.988	ICD-10	Substance	Other psychoactive substance use, unspecified with other psychoactive substance-induced disorder
F19.99	ICD-10	Substance	Other psychoactive substance use, unspecified with unspecified psychoactive substance-induced disorder

© 2023 Brenner LA et al. *JAMA Network Open.*
