# Supplementary material for: Associations of Military-Related Traumatic Brain Injury With New-Onset Mental Health Conditions and Suicide Risk
Source: JAMA Netw Open. 2023 Jul 31;6(7):e2326296. doi: 10.1001/jamanetworkopen.2023.26296 (PMC10391302; doi:10.1001/jamanetworkopen.2023.26296)
Supplement: Supplement 2. — Data Sharing Statement [file jamanetwopen-e2326296-s002.pdf]

Data Sharing Statement

Brenner. Associations of Military-Related Traumatic Brain Injury With New-Onset Mental Health Conditions and Suicide Risk. *JAMA Netw Open*. Published July 31, 2023. doi:10.1001/jamanetworkopen.2023.26296

Data
Data available: No

Additional Information
Explanation for why data not available: VA and DOD have strict policies regarding data sharing.
